# Supplementary material for: Formaldehyde reacts with N-terminal proline residues to give bicyclic aminals
Source: Commun Chem. 2023 Jan 13;6:12. doi: 10.1038/s42004-022-00801-5 (PMC9839752; doi:10.1038/s42004-022-00801-5)
Supplement: Supplementary file 2 — Supplemental Information [file 42004_2022_801_MOESM2_ESM.pdf]

Supplementary Information

Formaldehyde Reacts With N-Terminal Proline Residues to Give Bicyclic Aminals

Tobias John[a], Elisabete Pires[a], Svenja S. Hester[b], Eidarus Salah[a], Richard J. Hopkinson[c], and Christopher J. Schofield[a]

[a]Chemistry Research Laboratory, 12 Mansfield Road, Oxford, OX1 3TA, United Kingdom. [b]Nuffield Department of Medicine, Target Discovery Institute, University of Oxford, Oxford, United Kingdom. [c]Leicester Institute for Structural and Chemical Biology and School of Chemistry, University of Leicester, Henry Wellcome Building, Lancaster Road, Leicester, LE1 7RH, United Kingdom.

Reagents

Paraformaldehyde, DCDO, ^{13}C-labelled formaldehyde ($H^{13}CHO$), acetaldehyde, NaOD, DCl, tricine, 1-Boc-*L*-azetidine-2-carboxylic acid, *N*-methylmorpholine, *L*-glutathione (reduced form), 1-Boc-*L*-proline-2-carboxylic acid, 1-Boc-*L*-piperidine-2-carboxylic acid, 1-Boc-*L*-alanine-2-carboxylic acid, iodoacetamide acid, acetaldehyde and 1,3-cyclohexanedione were from Sigma-Aldrich. Other chemicals used were H-*L*-Glu(OtBu)-OtBu*HCl (Iris Biotech), dithiothreitol (Apollo Scientific), *L*-cysteine (Chem-Impex), EDC hydrochloride (Fluorochem), diazolidinyl urea (MP Biomedicals), imidazolidinyl urea (Alfa Aesar), cycloheximide (Cayman Chemical, CAY-14126-1), *trans*-β-nitrostyrene (Acros Organics 386530250).

Characterisation Methods

NMR spectra (HSQC, HMBC, COSY, ^{1}H, ^{13}C) were obtained using either Bruker Avance AV700, AV600, AV500, or AV400 spectrometers. To reference spectra to the solvent peak, the following shifts were used: δ_H = D_2O 4.79 ppm; δ_H = D_6-DMSO 2.5 ppm, δ_C = D_6-DMSO 39.52 ppm; δ_H = $CDCl_3$ 7.26 ppm, δ_C = $CDCl_3$ 77.16 ppm; δ_H MeOD = 3.31 ppm, δ_C = MeOD 49 ppm. For reporting multiplicities, the following abbreviations are used: m (multiplet), q (quartet), t (triplet), d (doublet), dd (doublet of doublets), s (singlet). For ^{1}H-^{13}C-HSQC and ^{1}H-^{13}C-HMBC spectra, colour codes are as follows: red HSQC signals denote CH or CH_3, blue HSQC signals denote CH_2 observed coupling, and green signals denote HMBC observed coupling. A Waters LCT Premier ESI mass spectrometer was used to obtain high resolution electrospray ionisation mass spectra (HRMS). An LC/MS Agilent Technologies 1200 series instrument was used to obtain other electrospray ionisation mass spectra.

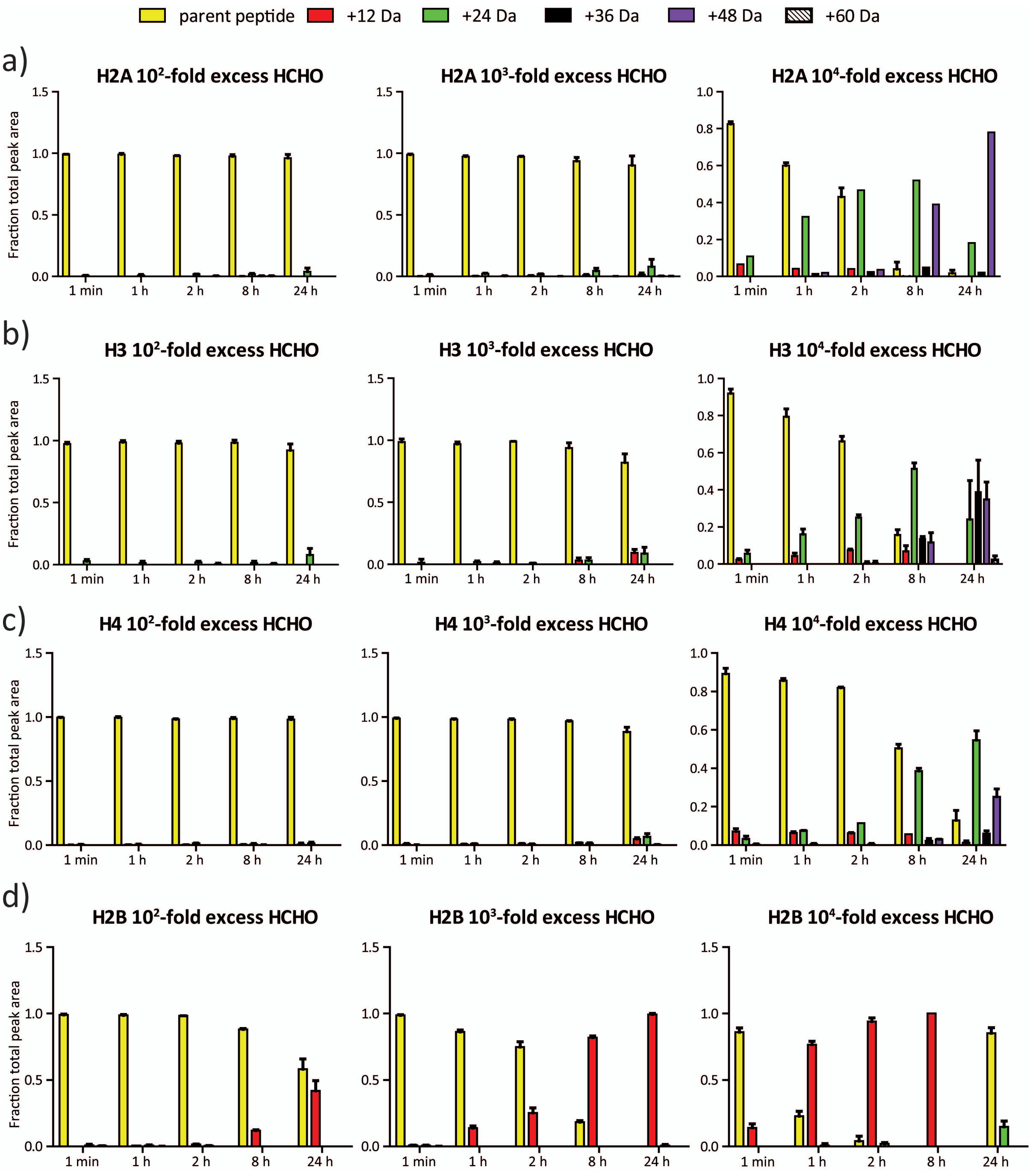

Figure S1 | Reaction of histone peptides with formaldehyde (HCHO). Different concentrations of HCHO (10^2- to 10^4-fold excess) were reacted with the N-terminal 15mer peptides from H2A (a), H3 (b), H4 (c), and H2B (d) (pH 7.4 in 50 mM potassium phosphate, ambient temperature) with reaction monitoring by MALDI MS. Errors: standard deviation of the mean (n=3, technical repeats). No adduct formation was observed for any of the peptides with a 10-fold excess or equimolar amounts of HCHO (data not shown). H2A 1-15 (NH_2-SGRGKQGGKARAKAK-NH_2); H3 1-15 (NH_2-ARTKQTARKSTGGKA-NH_2); H4 1-15 (NH_2-SGRGKGGKGLGKGGA-NH_2); H2B 1-15 (NH-PEPAKSAPAPKKGSK-NH_2).

H2B 10^2-fold excess HCHO, 37 °C

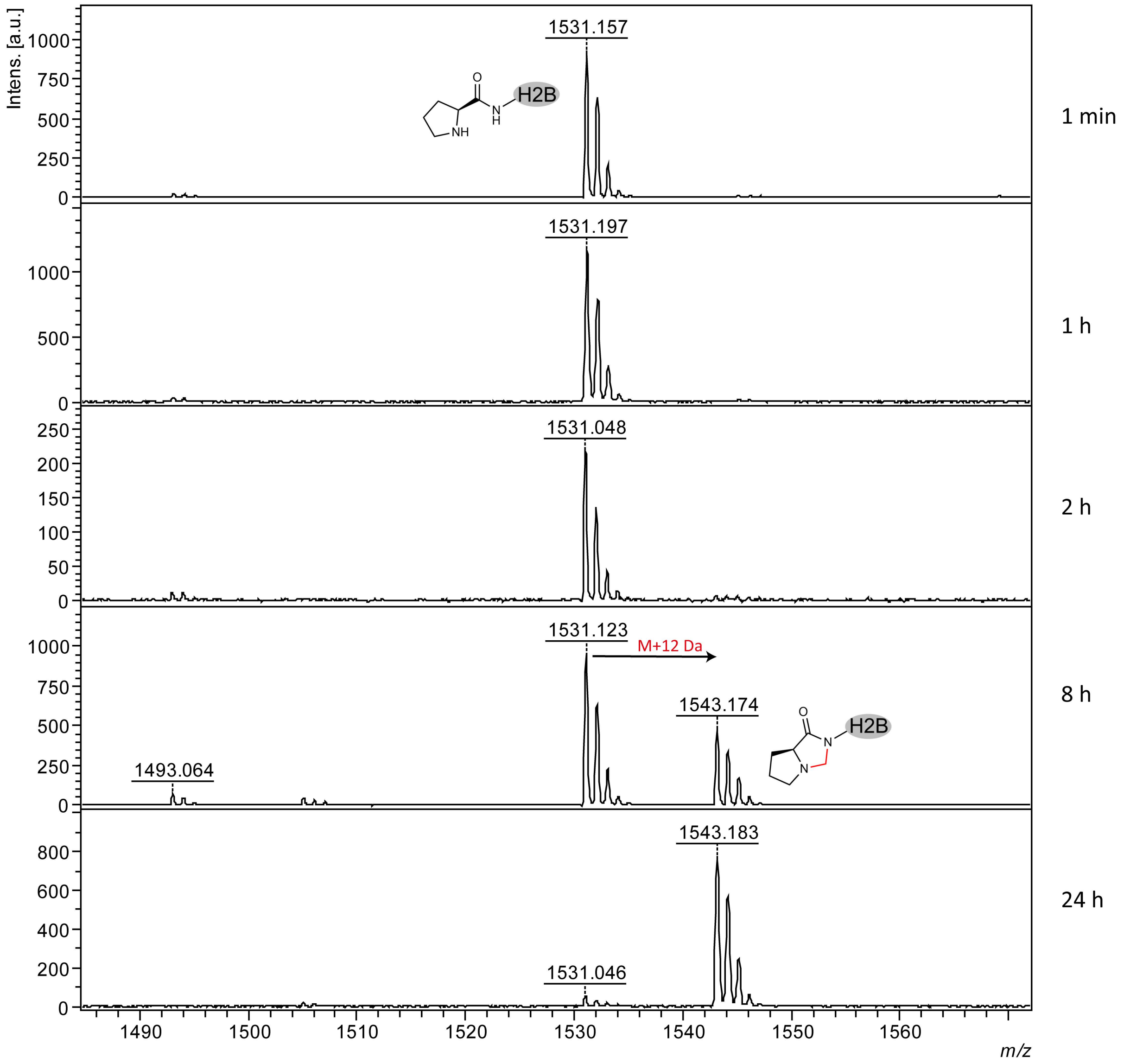

Figure S2 | Mass spectrometric data obtained from the reaction of HCHO with a H2B 15 residue peptide. The reaction mixture (2.5 µM peptide, 250 µM HCHO, 50 mM potassium phosphate at pH 7.4 and 37 °C) was analysed directly after addition of HCHO (1 min), after 1 h, 2 h, 8 h and 24 h by MALDI MS. Peaks are consistent with: parent peptide $[M+H]^+$: *m/z* 1493; parent peptide $[M+K]^+$: *m/z* 1531; +12 Da HCHO adduct $[M+K]^+$: *m/z* 1543. Peak areas were used to generate the plot in **Figure 1c**. For all other plots based on MALDI MS, data processing was performed similarly. H2B 1-15 (NH-PEPAKSAPAPKKGSK-NH_2).

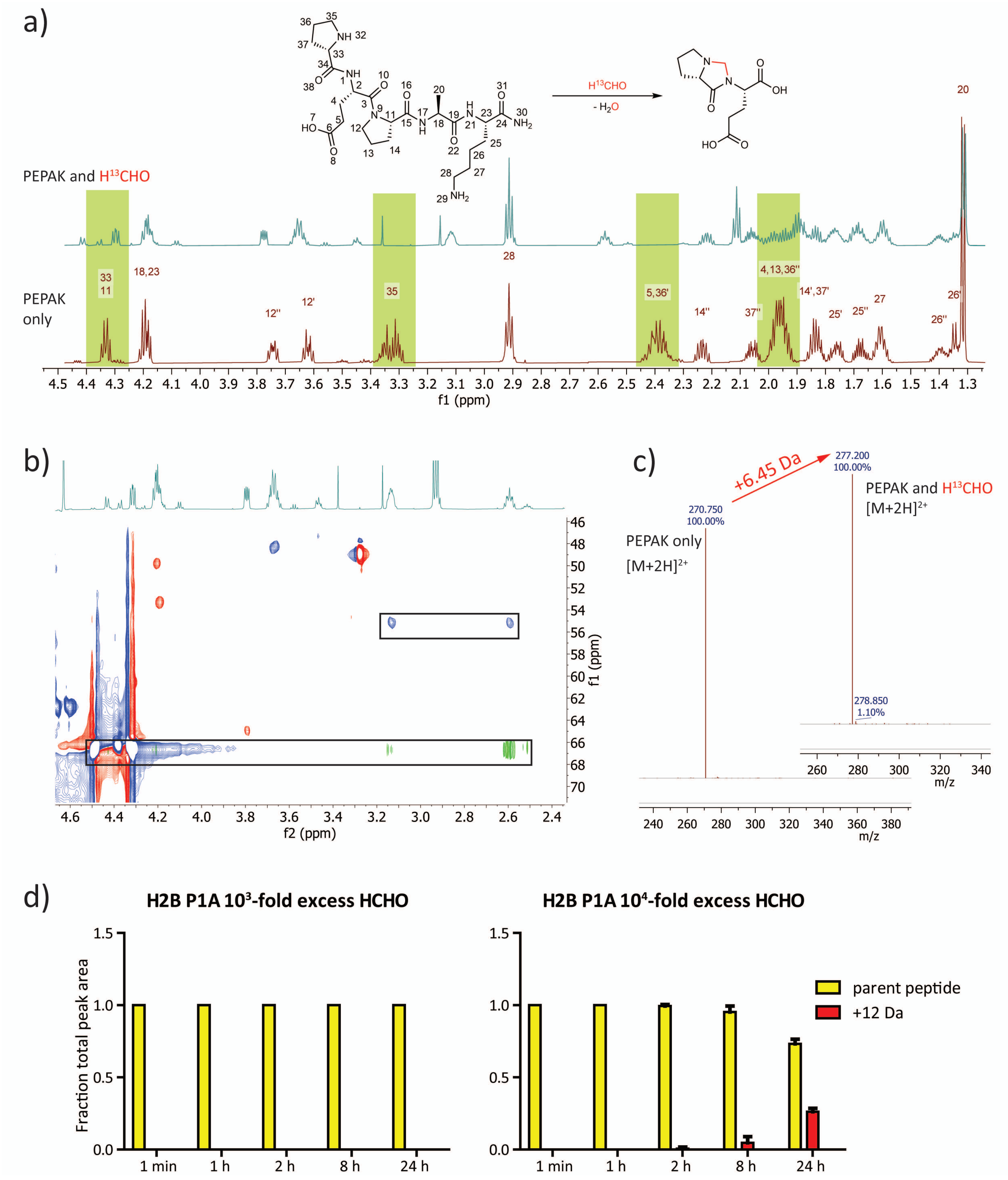

Figure S3 | The N-terminal proline of H2B reacts with HCHO. **a)** ^{1}H NMR (700 MHz) of the H2B pentapeptide (H2B 1-5 NH-PEPAK-NH_2) before (bottom panel) and after addition of $H^{13}CHO$ (top panel, prepared *in situ*). Differences are highlighted with green shading. **b)** Overlay of 2D ^{1}H-^{13}C HSQC and 2D ^{1}H-^{13}C HMBC spectra of the reaction of the H2B pentapeptide with $H^{13}CHO$. Signals of interest are highlighted with a black rectangle. **c)** LC/MS spectra of the H2B pentapeptide before and after reaction with $H^{13}CHO$ (*in situ*), showing a mass increase of 12.9 Da, consistent with methylene bridge formation. **d)** The H2B P1A 15mer was reacted with a 10^3- and 10^4-fold excess of HCHO (pH 7.4, ambient temperature). The reaction was monitored over 24 h by MALDI MS. Errors: standard deviation of the mean (n=3, technical repeats). H2B P1A 1-15 (NH_2-AEPAKSAPAPKKGSK-NH_2).

a)

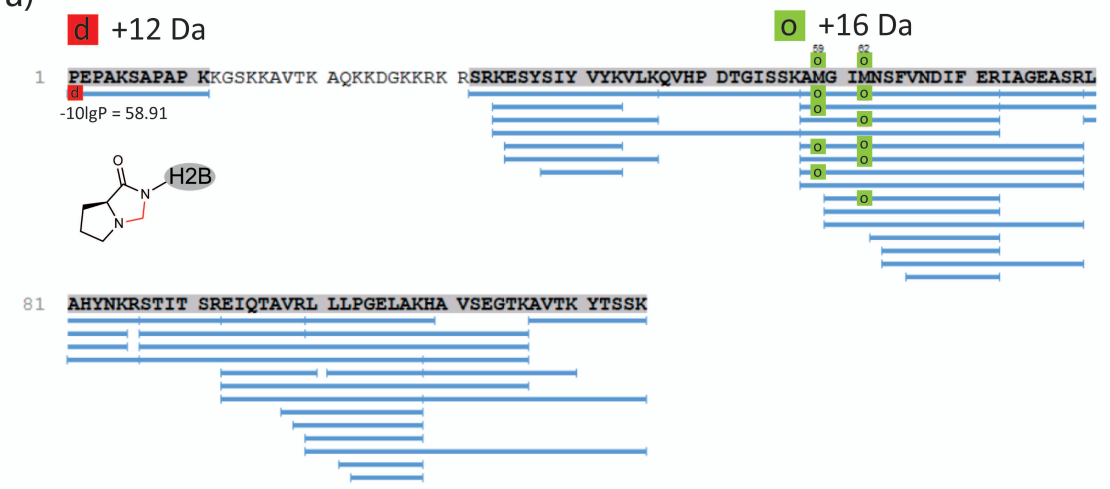

b)

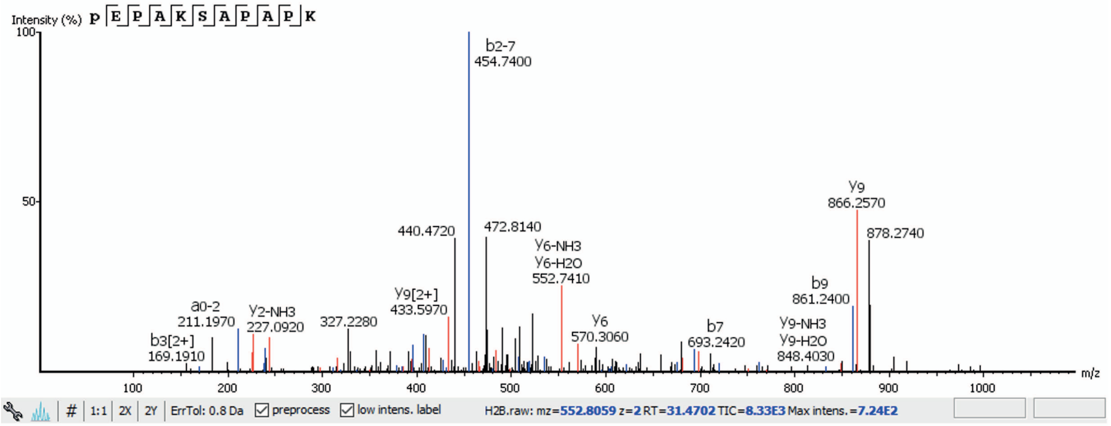

#	Immonium	b	b-H2O	b-NH3	b (2+)	Seq	y	y-H2O	y-NH3	y (2+)	#
1	82.0666	110.0616	92.0510	93.0346	55.5308	P(+12.00)					11
2	102.0554	239.1000	221.0936	222.0772	120.0521	E	995.5519	977.5414	978.5250	498.2470	10
3	70.0656	335.9930	318.7880	318.7880	169.1910	P	866.2570	848.4030	849.1510	433.5970	9
4	44.0499	407.2060	389.1835	390.1671	204.0970	A	769.5180	750.8710	752.4296	385.0760	8
5	101.1078	534.8470	517.5970	518.7880	268.1445	K	698.3970	680.4089	681.3250	350.1150	7
6	60.0449	621.9390	603.7810	605.3640	311.1960	S	570.3060	552.7410	552.7410	285.6623	6
7	44.0499	693.2420	675.4030	676.3311	347.1790	A	483.3150	465.1620	466.8920	242.1463	5
8	70.0656	790.4109	772.4003	773.3839	395.1170	P	412.6580	394.0410	395.1170	206.6277	4
9	44.0499	861.2400	843.4374	844.4210	431.4160	A	315.1970	297.2080	298.1757	158.1013	3
10	70.0656	958.5007	940.4902	941.4738	479.9740	P	244.1240	226.0210	227.0920	122.5828	2
11	101.1078					K	147.1128	129.1022	130.0858	74.0564	1

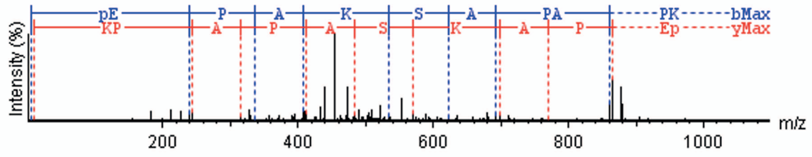

Figure S4 | Reaction of recombinant H2B with HCHO. Recombinant H2B was reacted with a 100-fold excess of HCHO for 24 hours at ambient temperature, then subjected to trypsin digestion, followed by LTQ Orbitrap Elite LC-MS/MS analysis. **a)** Results summary showing evidence that the N-terminal proline bears a +12 Da shift with a –10lgP>20. **b)** Raw data highlighting relevant b ions (blue) bearing the N-terminal +12 Da shift (e.g. the b_2-type peptide ion ProGlu: *m/z* 239.10).

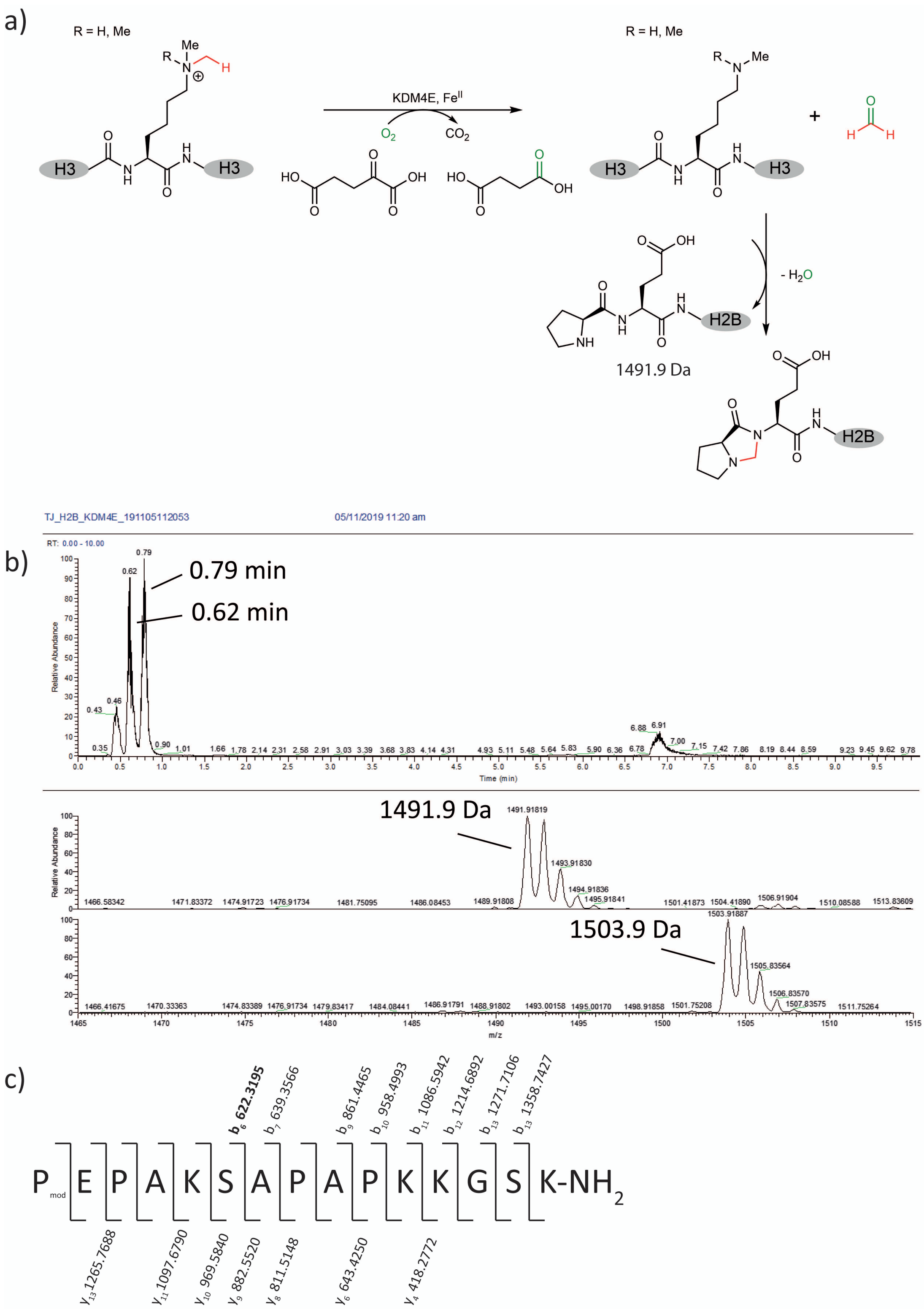

Figure S5 | H2B reacts with *in situ*-generated HCHO. a) Demethylation of H3K9Me$_3$ is catalysed by KDM4E, resulting in formation of HCHO. HCHO reacts with the N-terminus of the H2B 15mer peptide. **b)** (Bottom) LC/MS results after 24 hours incubation of KDM4E with H3K9Me$_3$ which enzymatically generates HCHO. (Top) Elution profile showing retention time (RT) of unreacted H2B 15mer at 0.62 min (*m/z* 1491.9, unreacted H2B 15mer) and the modified H2B at 0.79 min (*m/z* 1503.9, resembling M+12 Da). **c)** MS/MS results summary showing that the observed +12 Da shift occurs on the N-terminus of the H2B 15mer peptide (b$_6$ in bold). H3K9Me$_3$ 1-21 (NH$_2$-ARTKQTARK(Me$_3$)STGGKAPRKQLA-NH$_2$).

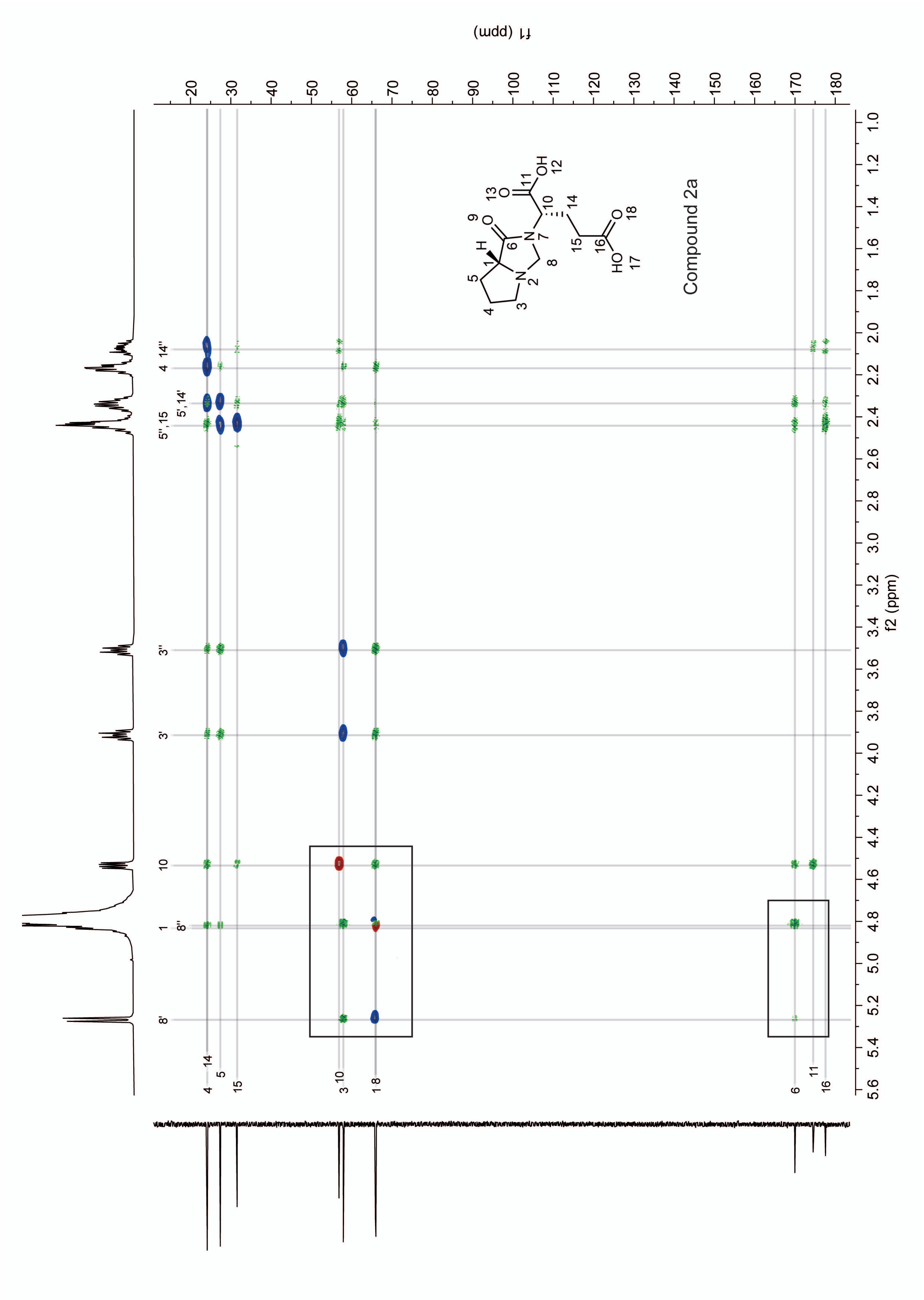

Figure S6 | Overlay of 2D ^{1}H-^{13}C HSQC and 2D ^{1}H-^{13}C HMBC spectra for HPLC-purified ProGlu-derived 2a (600 MHz). Important interactions are highlighted in rectangles.

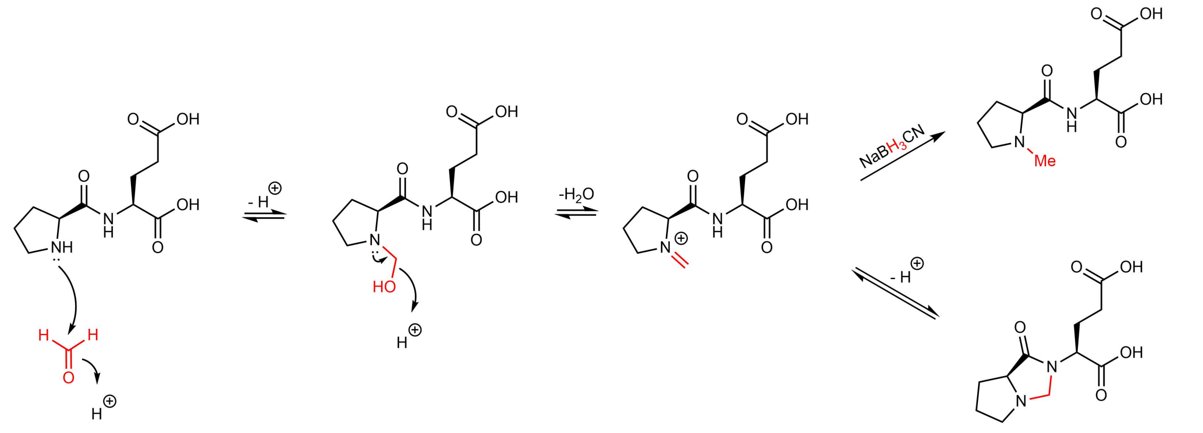

Figure S7 | Outline potential mechanism for methylene bridge formation to give a bicyclic adduct. Nucleophilic attack of the N-terminal nitrogen on HCHO results in hemiaminal and subsequent iminium ion formation. A methylene bridge is formed via overall 5-endo-trig type cyclisation (lower path), resulting in **2a**. The iminium species was reduced with $NaBH_3CN$ to yield an *N*-methylated proline residue (upper path).

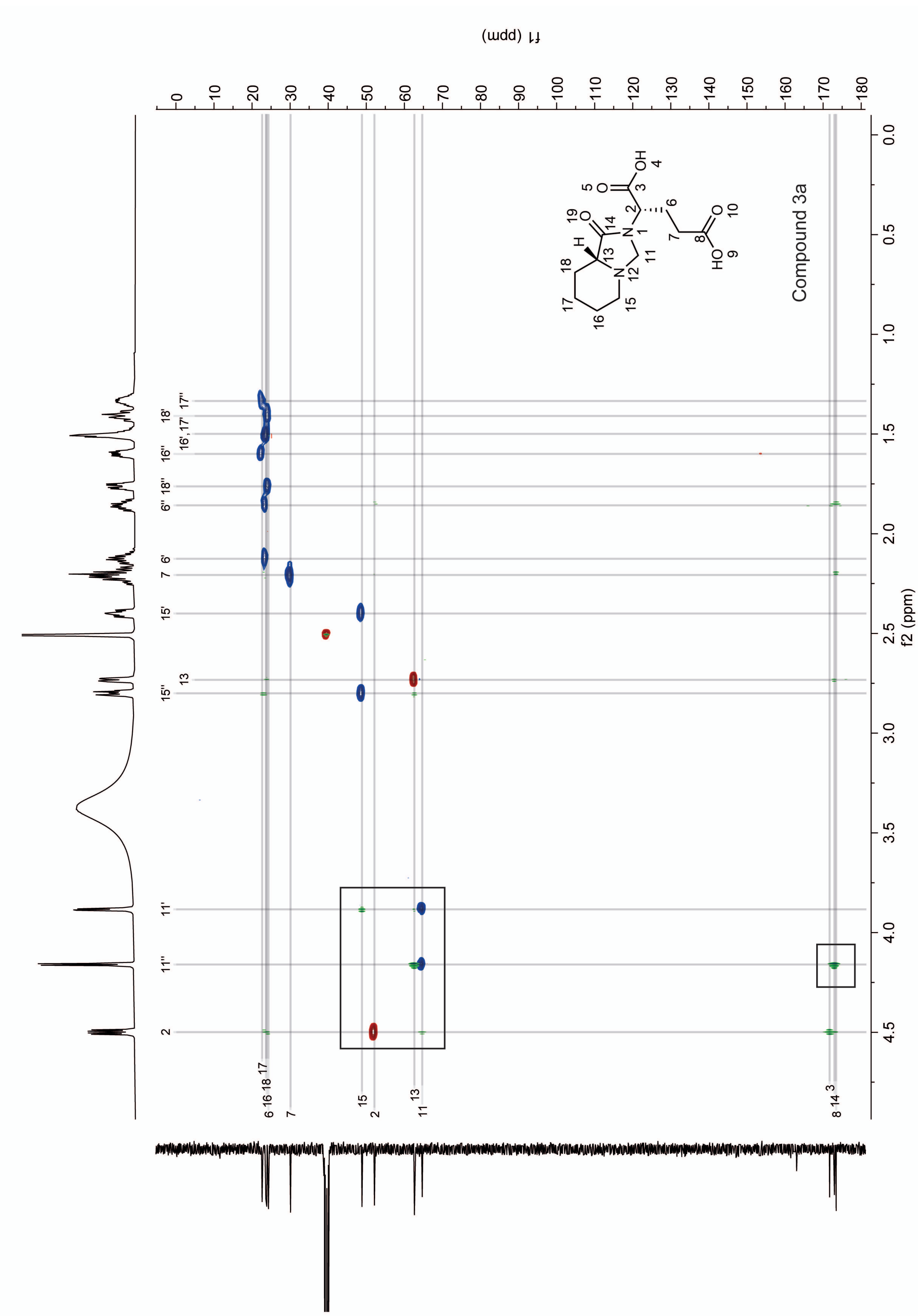

Figure S8 | Overlay of 2D ^{1}H-^{13}C HSQC and 2D ^{1}H-^{13}C HMBC spectra for HPLC-purified PipGlu-derived 3a (700 MHz). Important interactions are highlighted in rectangles. The x-axis shows the ^{1}H spectrum and the y-axis shows the ^{13}C spectrum.

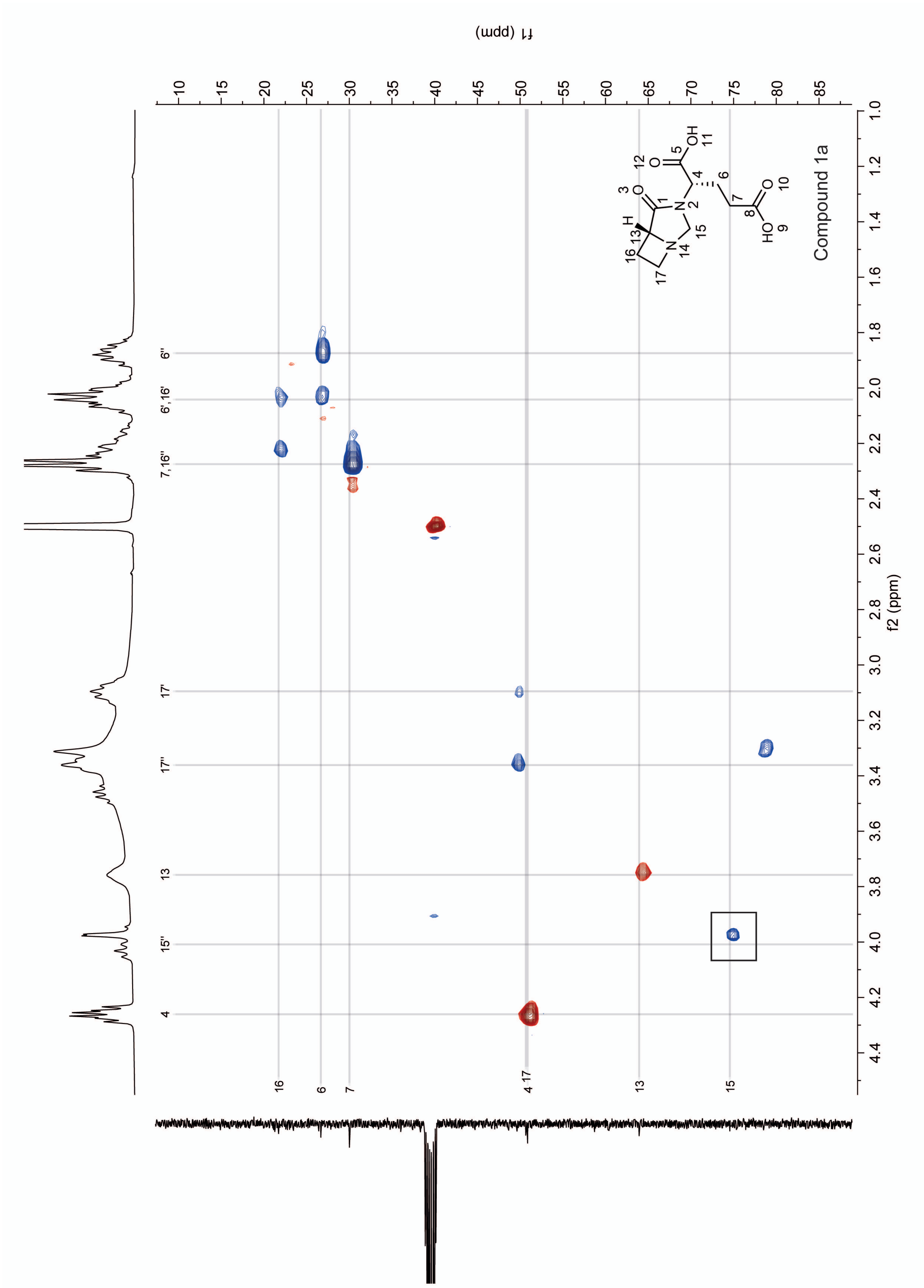

Figure S9 | 2D ^{1}H-^{13}C HSQC spectrum for HPLC-purified AzeGlu-derived 1a (400 MHz). Note the peak at δ_H 3.98 and δ_C 74.6 ppm was tentatively assigned to a methylene group in the bridged bicycle (**1a**); this signal was not observed when the sample was measured again at a later time-point (6 days later, see **Figure S10**), indicating that the methylene group is relatively unstable in the putative 4,5-bicyclic ring system.

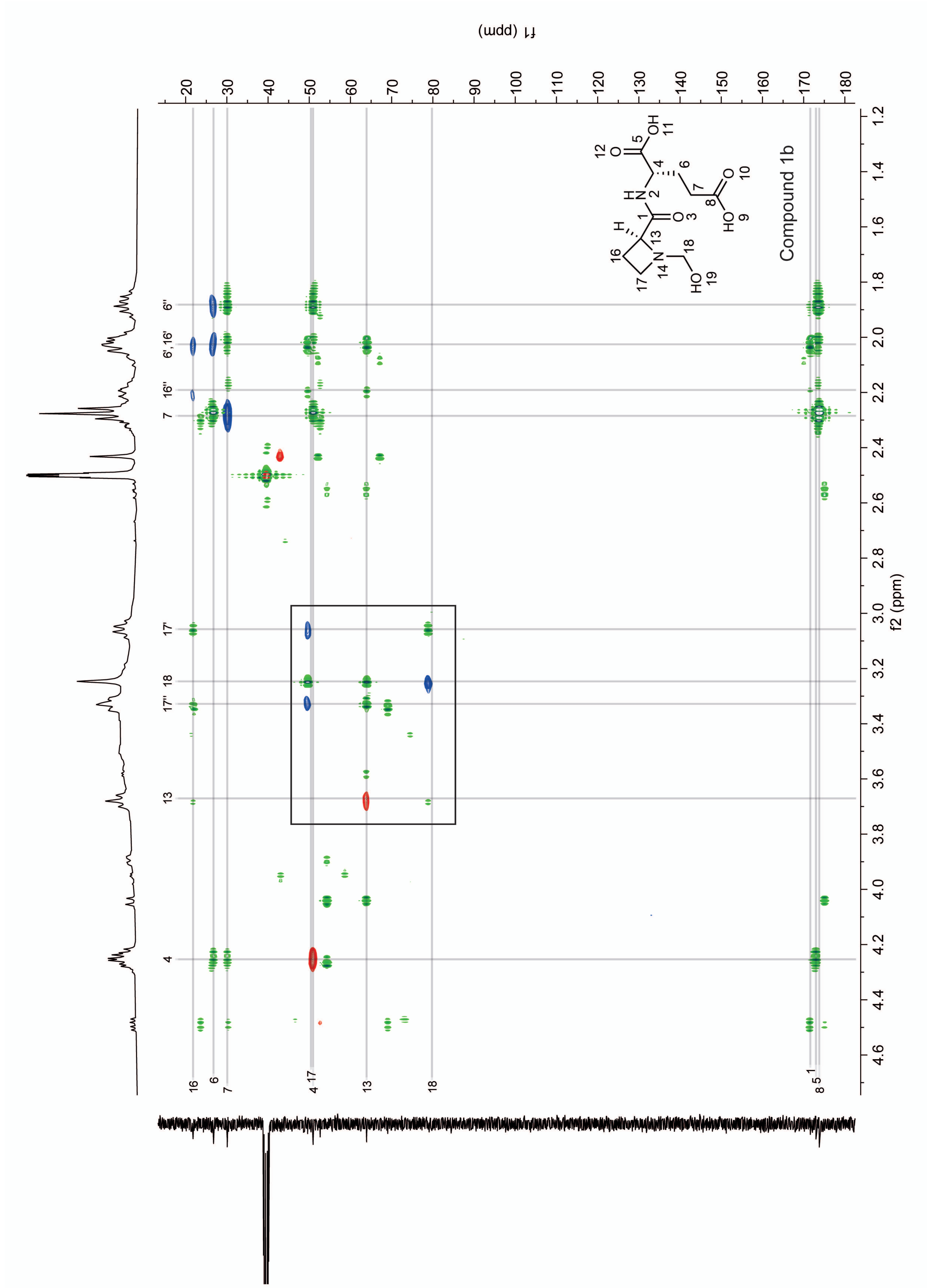

Figure S10 | Overlay of 2D ^{1}H-^{13}C HSQC and 2D ^{1}H-^{13}C HMBC spectra for HPLC-purified AzeGlu-derived 1b after 6 days (400 MHz). Note that the signals at δ_H 3.98 and δ_C 74.6 ppm from **Figure S9** are no longer observed and new signals (C18) at δ_H 3.25 and δ_C 79.8 ppm appear, which are tentatively assigned to a hemiaminal (**1b**). These signals are also present in **Figure S9** at low levels. Important interactions are highlighted in the rectangle.

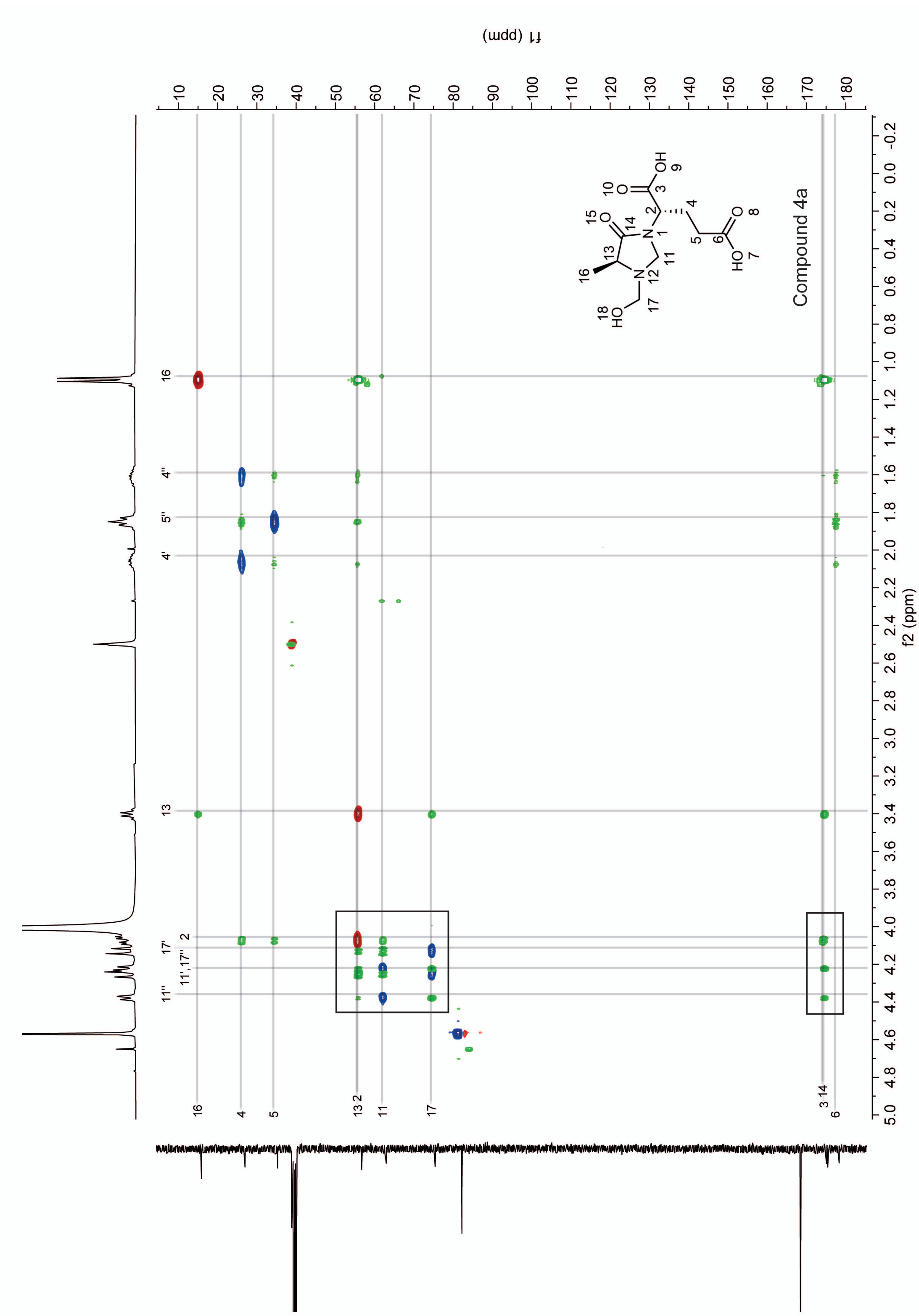

Figure S11 | Overlay of 2D ^{1}H-^{13}C HSQC and 2D ^{1}H-^{13}C HMBC spectra for HPLC-purified AlaGlu-derived 4a (400 MHz). The formation of a product (**4a**) with two methylene groups is observed on reaction of **4** with HCHO. One of these methylene bridges (C17) is assigned to a hemiaminal (δ_C 75.4, δ_H 4.13 and δ_H 4.24 ppm); the other methylene bridge (C11) is assigned to an aminal (δ_C 63.0, δ_H 4.38 and δ_H 4.24 ppm). Important interactions are highlighted in the rectangles.

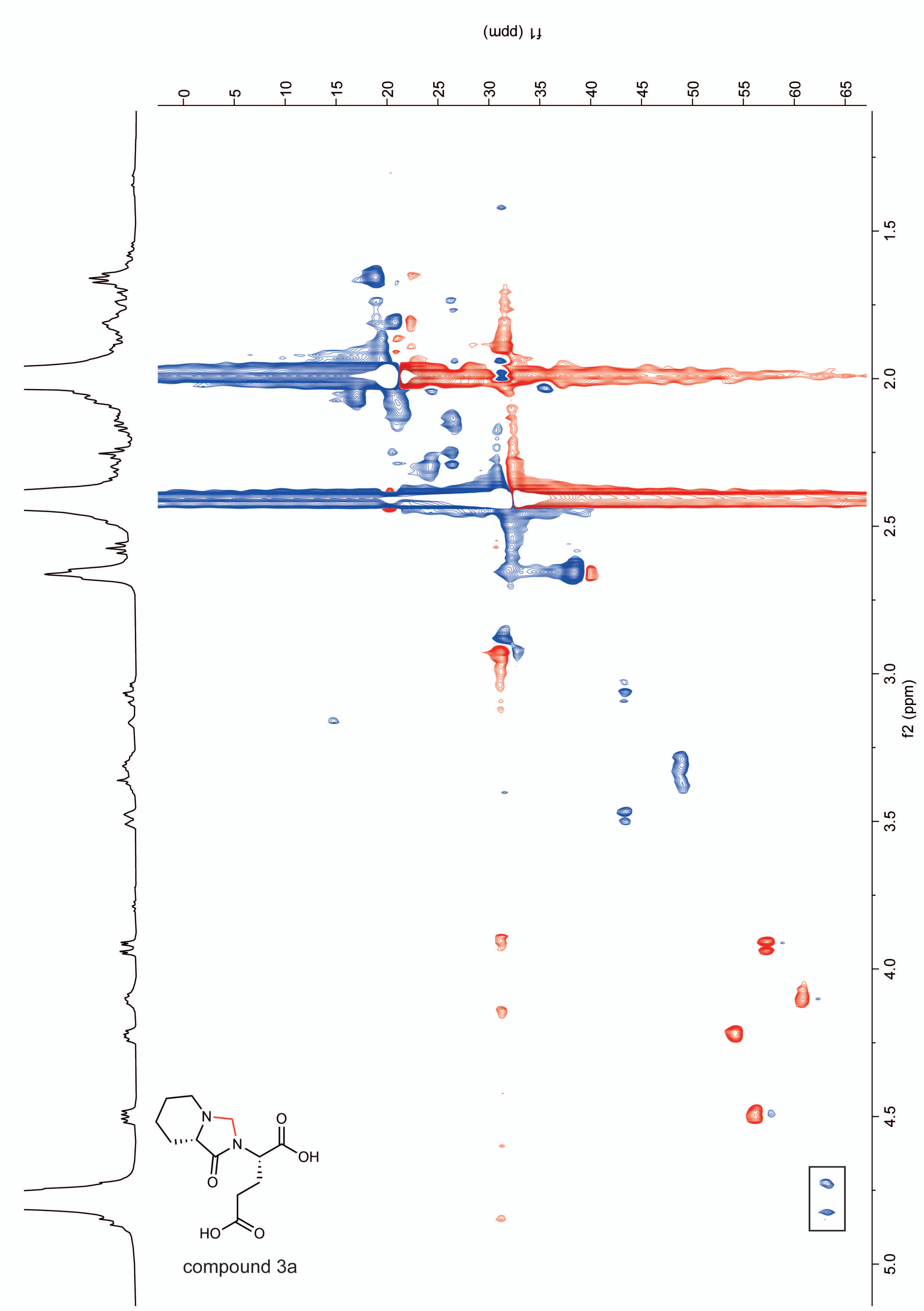

Figure S12 | 2D ^{1}H-^{13}C HSQC spectrum showing the result of the addition of a 40-fold excess of 1,3-cyclohexanedione to a solution of PipGlu-derived 3a (400 MHz). 3 was reacted with a 10-fold excess HCHO overnight to give **3a**, followed by addition of a 40-fold excess of 1,3-cyclohexanedione. The methylene bridge marked in a rectangle corresponds to the methylene bridge of HPLC-purified **3a** at δ_C 64.7 ppm (**Figure S8**), suggesting that the 6,5-bicyclic adduct **3a** is relatively stable.

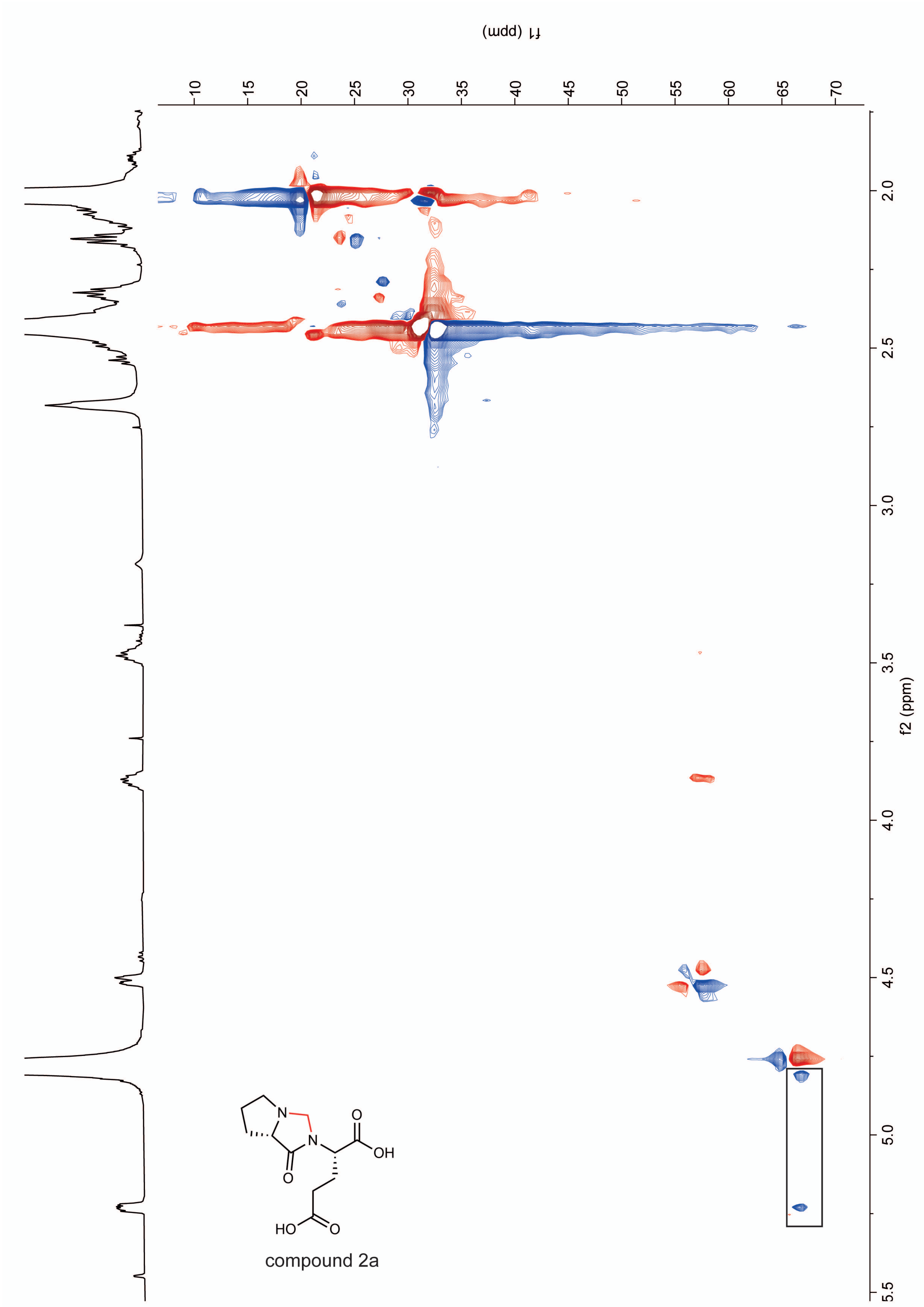

Figure S13 | 2D ^{1}H-^{13}C HSQC spectrum showing the result of the addition of a 40-fold excess of 1,3-cyclohexanedione to a solution of ProGlu-derived 2a (600 MHz). 2 was reacted with a 10-fold excess HCHO overnight to give **2a**, followed by addition of a 40-fold excess of 1,3-cyclohexanedione. The methylene bridge marked in a rectangle corresponds to the methylene bridge of HPLC purified **2a** at δ_C 66.7 ppm (**Figure S6**), suggesting that the 5,5-bicyclic adduct **2a** is relatively stable.

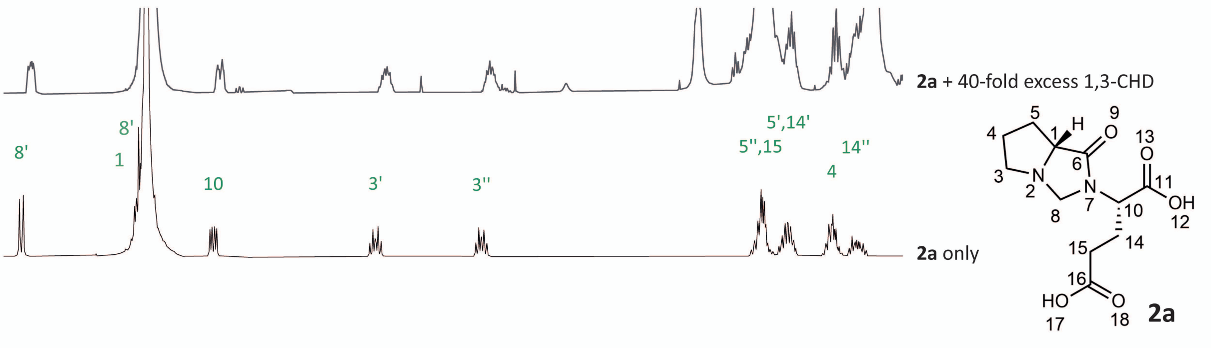

Figure S14 | | Overlay of HPLC-purified 2a and 2a in the presence of excess 1,3-cyclohexanedione. ^{1}H NMR (600 MHz) showing HPLC purified **2a** as shown in **Figure S6** and **2a** in the presence of a 40-fold excess of 1,3-cyclohexanedione (1,3-CHD) as shown in **Figure S13**.

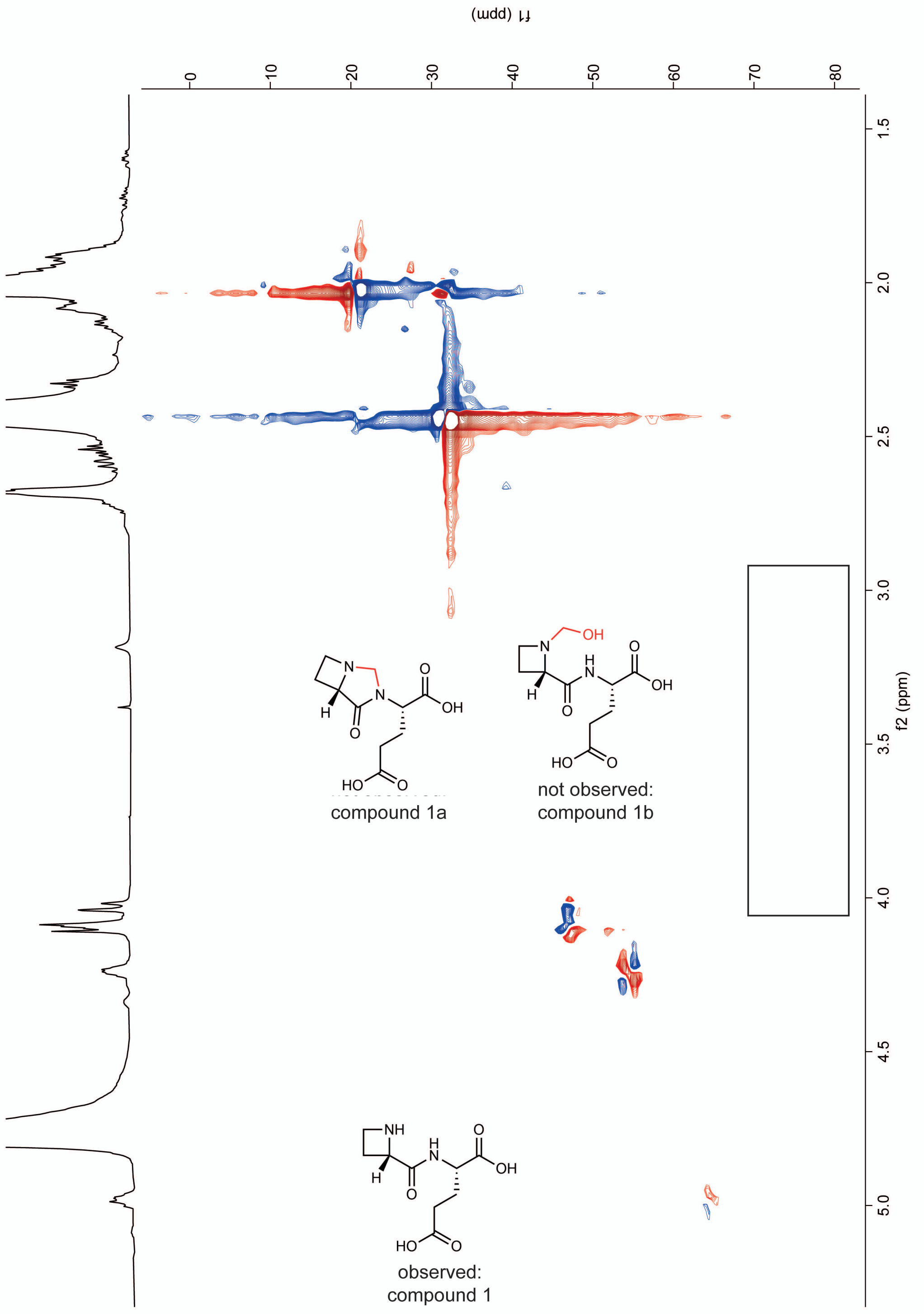

Figure S15 | 2D ^{1}H-^{13}C HSQC spectrum showing the result of the addition of a 40-fold excess of 1,3-cyclohexanedione to a solution of AzeGlu-derived 1a/b (600 MHz). 1 was reacted with a 10-fold excess HCHO overnight (resulting in formation of **1a** or **1b**), followed by addition of a 40-fold excess of 1,3-cyclohexanedione. The aminal or hemiaminal methylene bridges of **1a/b** are expected to appear between δ_C 75 and δ_C 80 ppm (marked in a rectangle in **Figure S9** and **Figure S10**); no signal was detected, suggesting the putative 4,5-bicyclic adduct **1a** is relatively unstable.

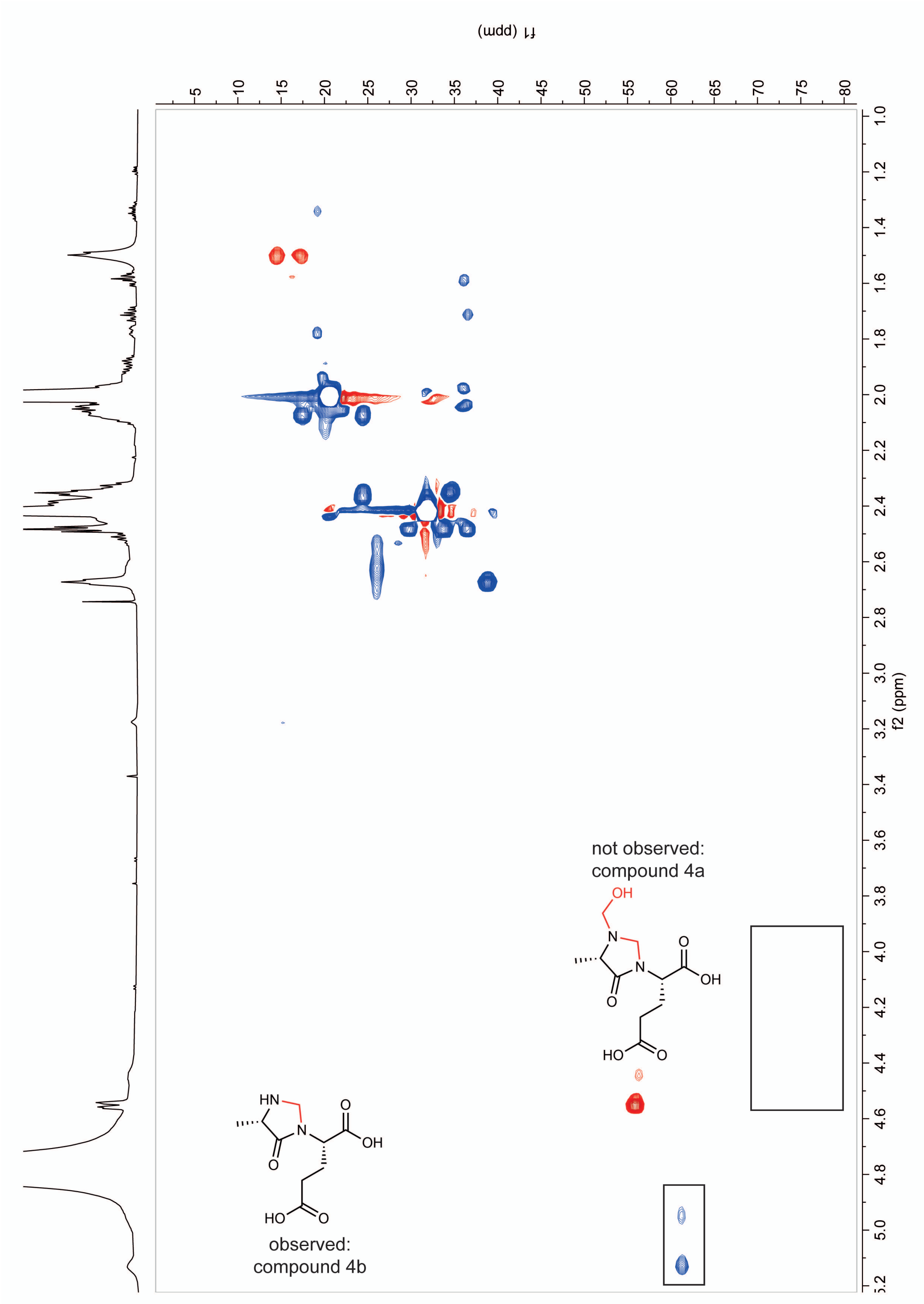

Figure S16 | 2D ^{1}H-^{13}C HSQC spectrum showing the result of the addition of a 40-fold excess of 1,3-cyclohexanedione to a solution of AlaGlu-derived 4a (700 MHz). 4 was reacted with a 10-fold excess of HCHO for 3 weeks to enable formation of **4a**, followed by addition of a 40-fold excess of 1,3-cyclohexanedione. The hemiaminal methylene bridge of **4a** is expected to appear at δ_C 75.4 ppm; no signal was detected in this area (empty rectangle), suggesting the hemiaminal is relatively unstable. However, the aminal bridge of **4b** (marked in a rectangle) corresponds to the aminal bridge of HPLC purified **4a** at δ_C 63.0 ppm (**Figure S11**). This observation suggests the HCHO-derived aminal, but not the hemiaminal methylene bridge of **4a**, is stable in the presence of excess 1,3-cyclohexandione, resulting in formation of **4b**.

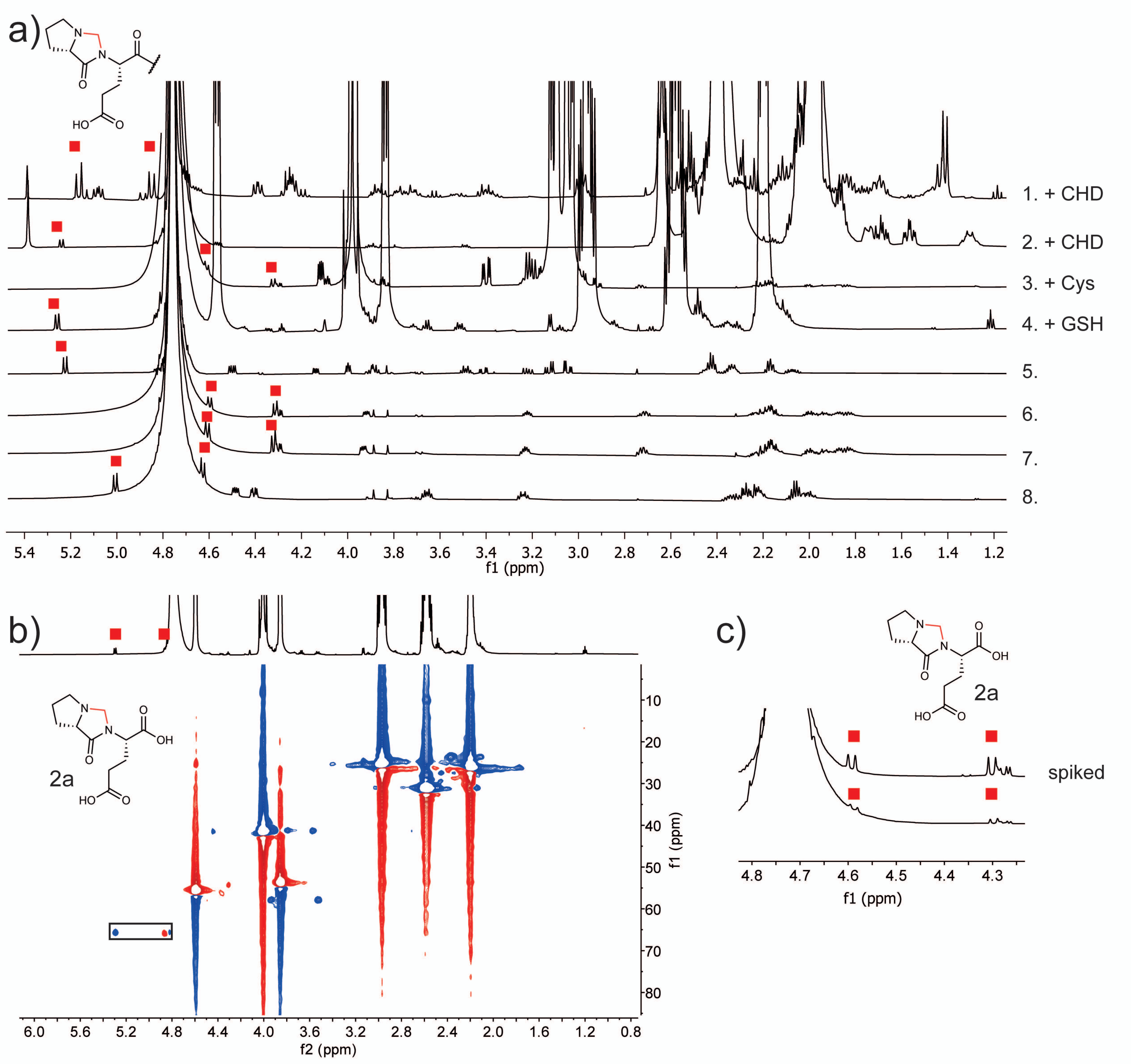

Figure S17 | Evidence that the methylene bridge between the proline nitrogen and the amide bond nitrogen is relatively stable in the presence of HCHO scavengers. a) ^{1}H NMR of the HPLC-isolated NH-PEPAK-NH_2 HCHO adduct (1., 400 MHz) or HPLC-isolated **2a** (2.-8.) in the presence of a 40-fold excess of competing nucleophiles (1,3-cyclohexanedione (1.+2.), cysteine (3.), and GSH (4.), 600 MHz), at 75 °C with 1 equivalent of cysteine (5., 600 MHz) or at different pH values (pD 8.9 (6.), pD 7.8 (7.), pD 6.3 (8.), 700 MHz). **b)** All results in (a) were supported by 2D ^{1}H-^{13}C HSQC measurements (the methylene bridge is marked in a rectangle), exemplified by **2a** in the presence of a 40-fold excess of GSH (600 MHz). **c)** Competition reactions in (a) were spiked with HPLC-purified **2a** to ensure correct assignment, here exemplified by spiking the mixture of **2a** in the presence of a 40-fold excess of cysteine (lower panel) with HPLC-purified **2a** (upper panel, 600 MHz). The methylene bridge signals of the **2a**/PEPAK HCHO adduct are marked in red throughout the figure.

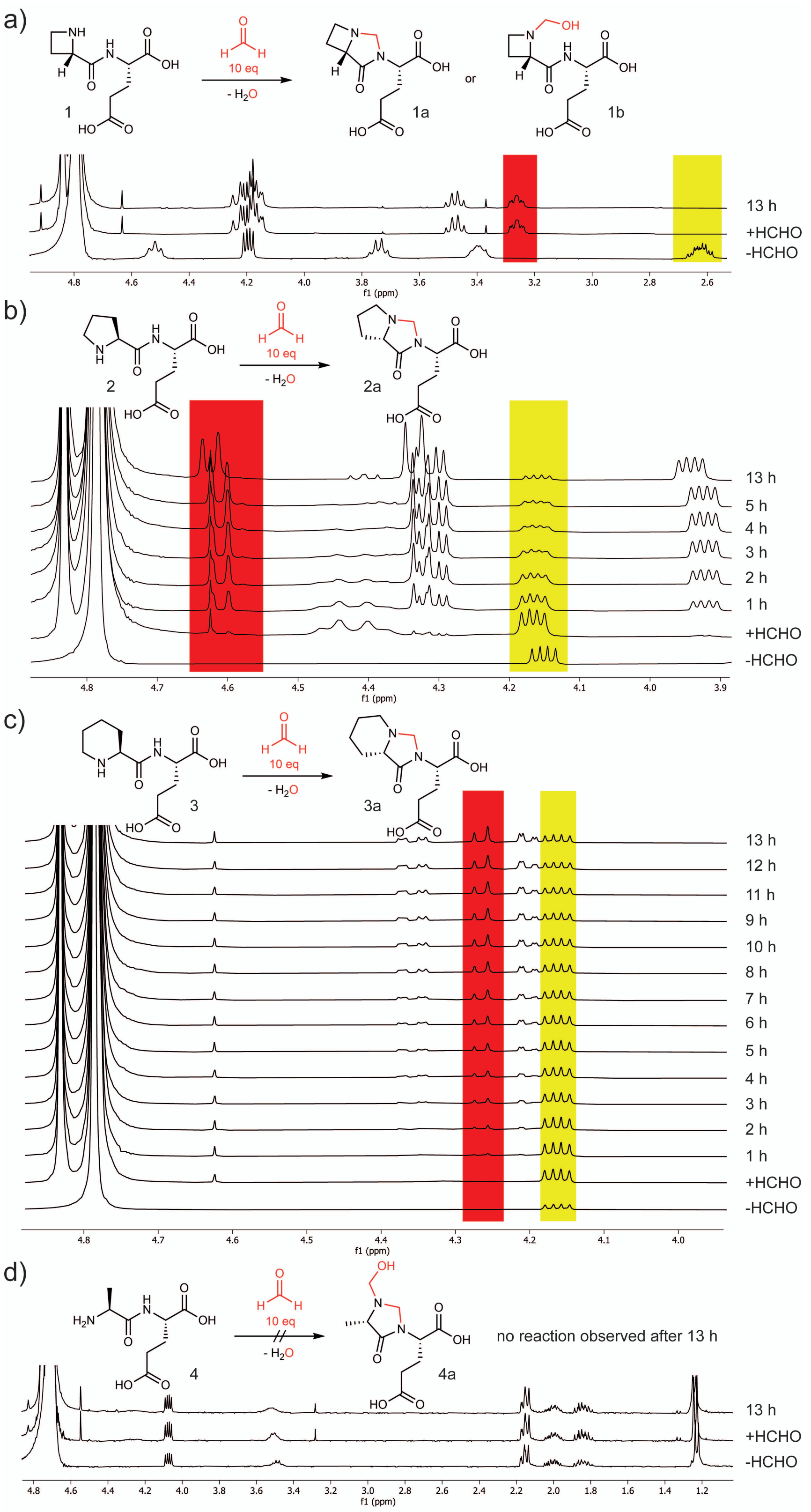

Figure S18 | ^{1}H NMR (400 MHz) time-course analyses of the reaction of dipeptides with HCHO. a-d) A 10-fold excess of HCHO (pD 10) was used. **1** reacts quickly, showing full conversion after addition of HCHO (a). **4** is extremely slow to react under these conditions (d). The first panel shows the dipeptide only (-HCHO). The second panel shows the spectrum taken directly after addition of HCHO (+HCHO). The yellow rectangle shows peaks used for quantification of starting material. The red rectangle shows peaks used for quantification of products. The expected products are shown above each time-course. The data were used to generate the plot in **Figure 3c**.

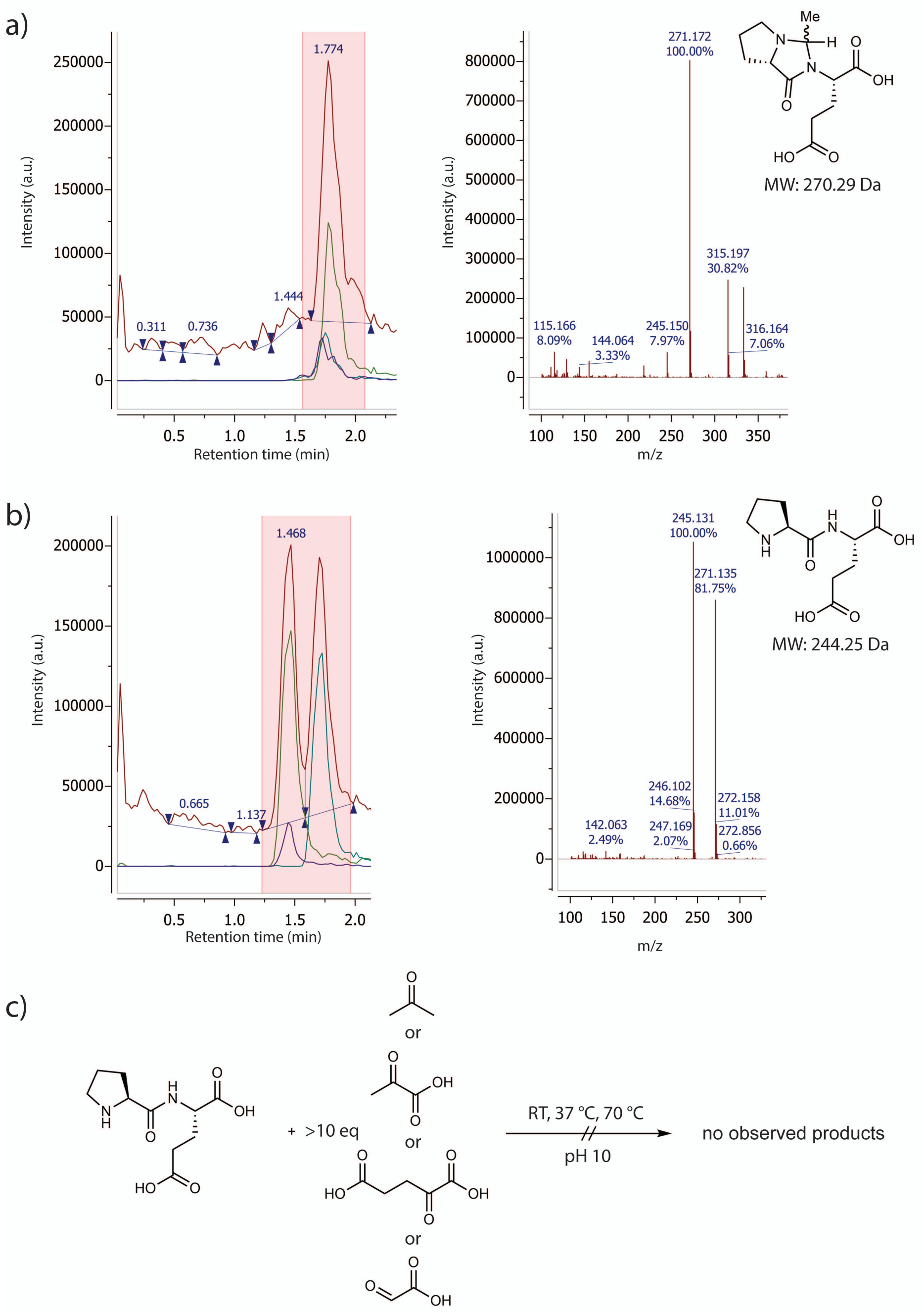

Figure S19 | Reactions of the H2B dipeptide with biologically relevant carbonyl compounds. a) LC/MS trace of the HPLC fraction containing the product generated by the reaction of a 100-fold excess acetaldehyde with **2** at 37 °C (left graph) showing full conversion of **2** to form an acetaldehyde adduct (*m/z*=271, right graph). **b)** LC/MS trace (left graph) upon lyophilisation reveals that the adduct is unstable, partially re-forming the starting material (**2**, m/z=245, right graph). **c)** Excess acetone, pyruvate, 2OG or glyoxylic acid were added to **2** at different temperatures (ambient temperature, 37 °C, and 70 °C), but no conversion was observed under the tested conditions, as determined by LC/MS (data not shown). This observation suggests these carbonyl compounds do not react efficiently with N-terminal proline residues, at least to give stable adducts.

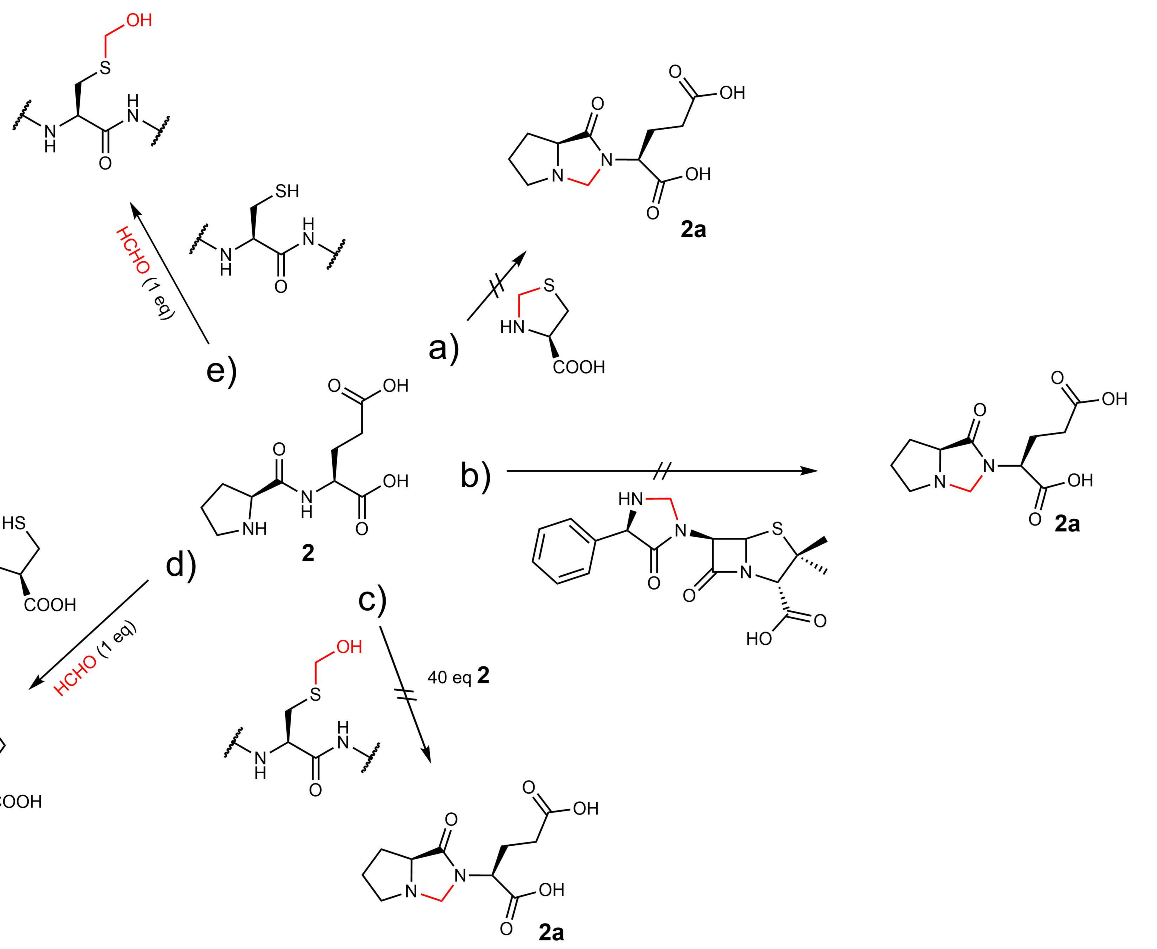

Figure S20 | Thiol groups apparently react faster with HCHO than compound 2. a+b) Equimolar amounts of **2** do not remove HCHO-derived methylene bridges from thioproline (a) or metampicillin (b) (see **Figure S21**). **c)** A 40-fold excess of **2** does not remove the HCHO-derived methylene bridge of *S*-(hydroxymethyl)glutathione (HMG) (see **Figure S21**). **d+e)** Cysteine (d) and glutathione (e) react with HCHO to form thioproline and HMG, respectively, in the presence of **2** (see **Figure S22**).

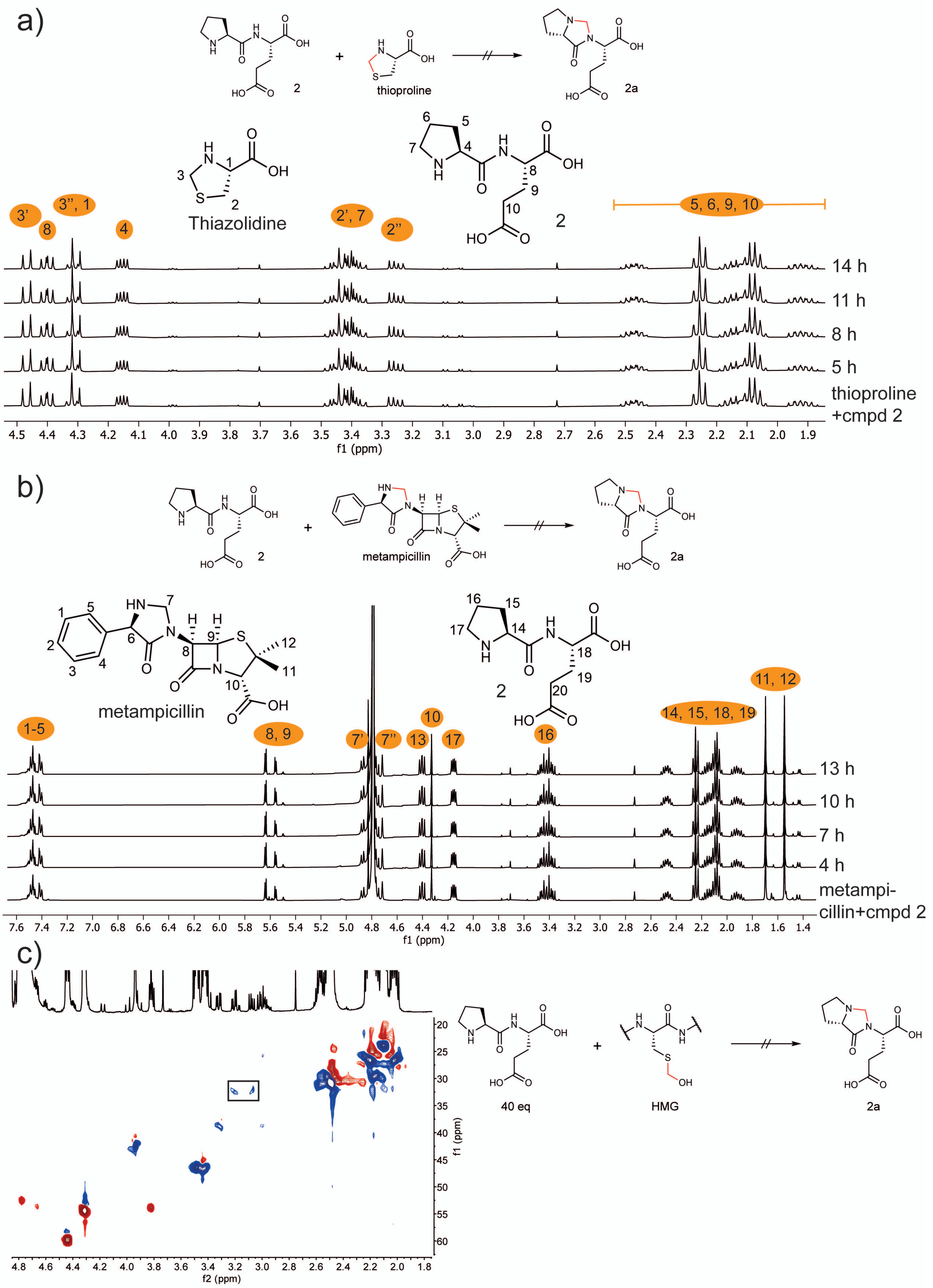

Figure S21 | Compound 2 does not react with molecules bearing HCHO-derived methylene bridges. a+b) HCHO was reacted with cysteine (a) or ampicillin (b) to generate thioproline and metampicillin *in situ* which were then each incubated with 1 equivalent of **2**; the reactions were monitored by ^{1}H NMR (400 MHz) showing the HCHO-derived methylene bridges of thioproline (a) and metampicillin (b) are not removed by addition of **2**. **c)** HCHO was reacted with glutathione (GSH) to generate *S*-(hydroxymethyl)glutathione (HMG) *in situ*, to which 40 equivalents of **2** were added, followed by monitoring the reaction by 2D ^{1}H-^{13}C HSQC (600 MHz). The HCHO-derived methylene bridge on HMG (marked with a rectangle) is not removed by excess **2** under the tested conditions.

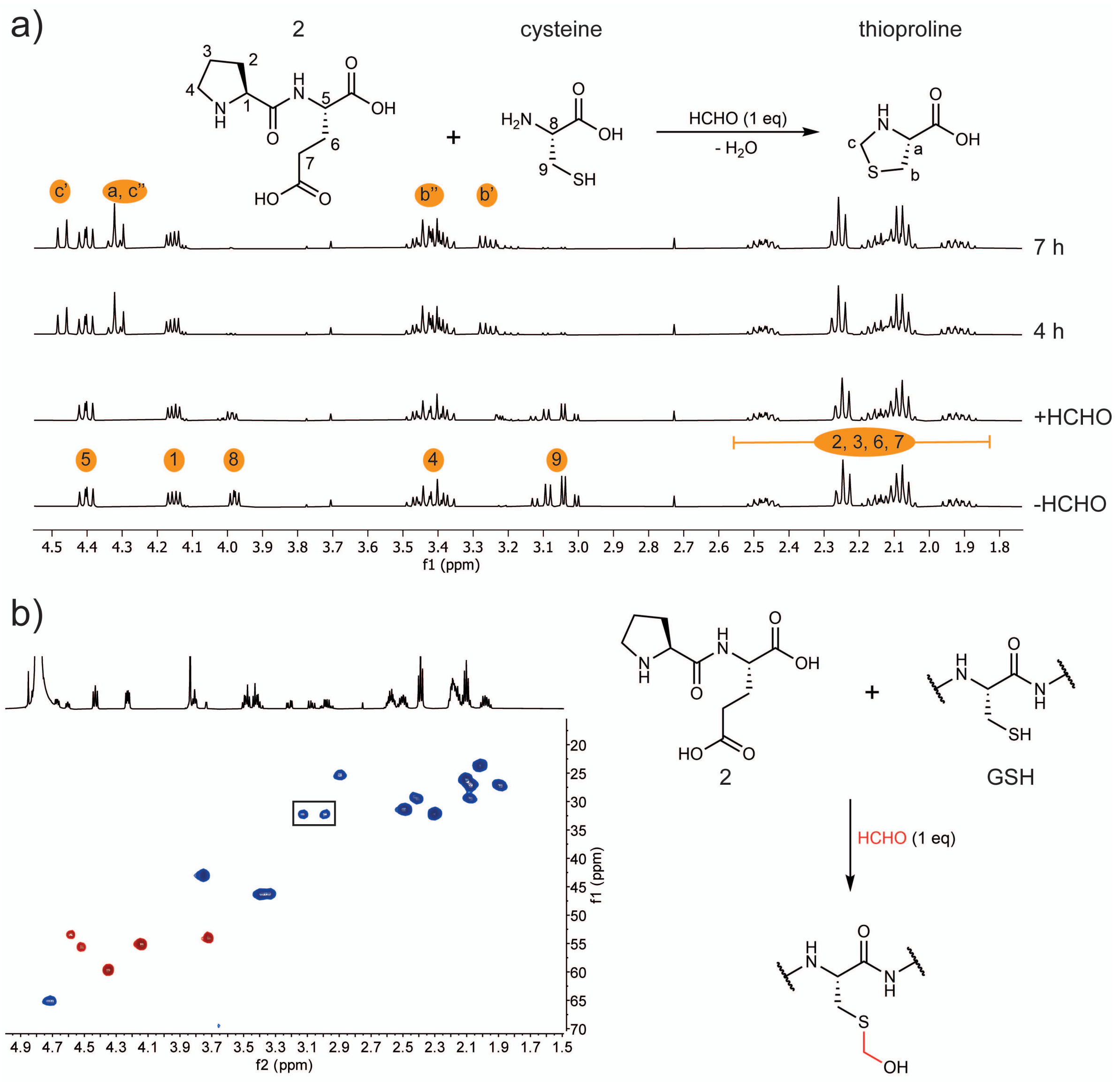

Figure S22 | Cysteine-derived thiol groups react more readily with HCHO than compound 2. **a)** 1 equivalent of HCHO was added to cysteine and **2**. Monitoring of the reaction over 7 h by ^{1}H NMR (400 MHz) shows cysteine reacts with HCHO to form thioproline. **b)** 2D ^{1}H-^{13}C HSQC (600 MHz) analysis of the reaction of GSH and **2** in the presence of 1 equivalent HCHO after 10 h. The rectangle shows the HCHO-derived hydroxymethyl adduct on *S*-(hydroxymethyl)glutathione (HMG).[1]

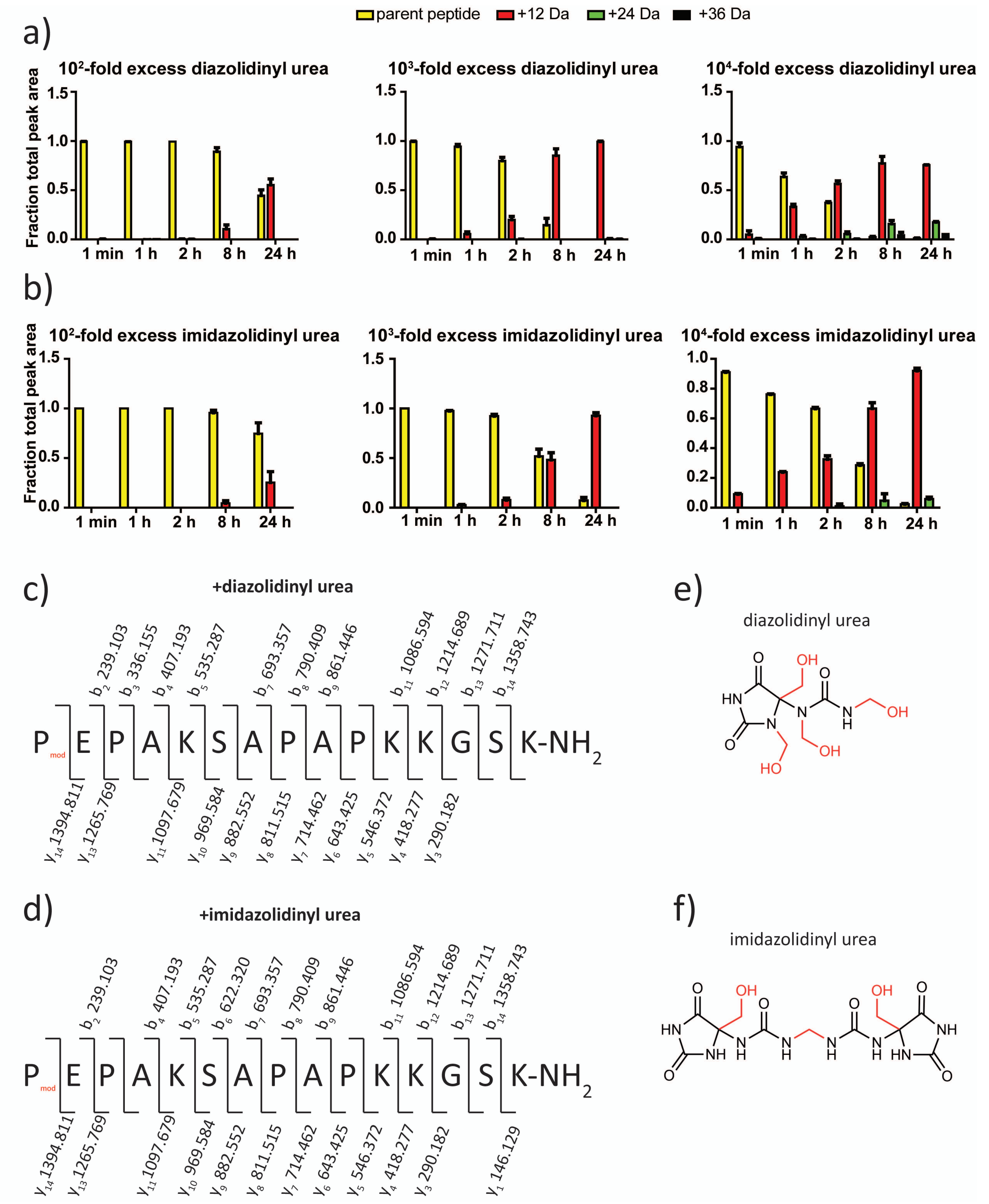

Figure S23 | The HCHO donors diazolidinyl urea and imidazolidinyl urea result in formation of an N-terminal HCHO adduct with the H2B 15mer. **a+b)** Diazolidinyl urea (a) or imidazolidinyl urea (b) were reacted (10- to 10^4-fold excess) with the H2B 15mer peptide (pH 7.4, ambient temperature). Reactions were monitored by MALDI MS over 24 h. Errors: SD of the mean (n=3, technical repeats). **c+d)** MALDI MS/MS results summary based on reaction of H2B 15mer after treatment with 1000-fold excess diazolidinyl urea (c) or imidazolidinyl urea (d) for 12 h. **e)** Structure of diazolidinyl urea as revised by Lehmann et al.[2] **f)** Structure of imidazolidinyl urea as revised by Lehmann et al.[2]

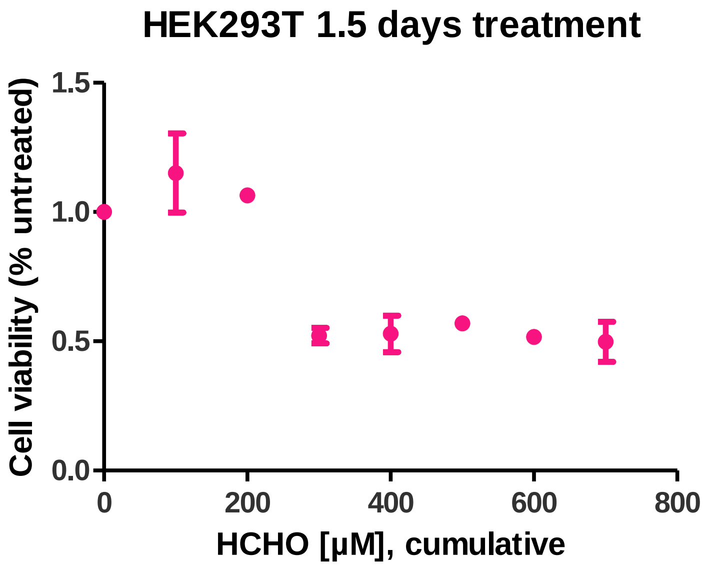

Figure S24 | HEK293T cell viability with two cumulative HCHO treatments. 0.01x10^6 cells were seeded in a 96-well plate. After the cells were attached (>8 h), they were treated with a first dose of HCHO, followed by a second dose of HCHO 24 h after that. The second dose was 1.5-fold higher than the first dose. Thus, for example, the data point at 200 µM HCHO means 80 µM HCHO, followed by 120 µM HCHO. After 14 h, viable cells were estimated using the CellTiter 96® AQueous One Solution Reagent (Promega, G358C). Errors: SD of the mean (n=3, technical repeats).

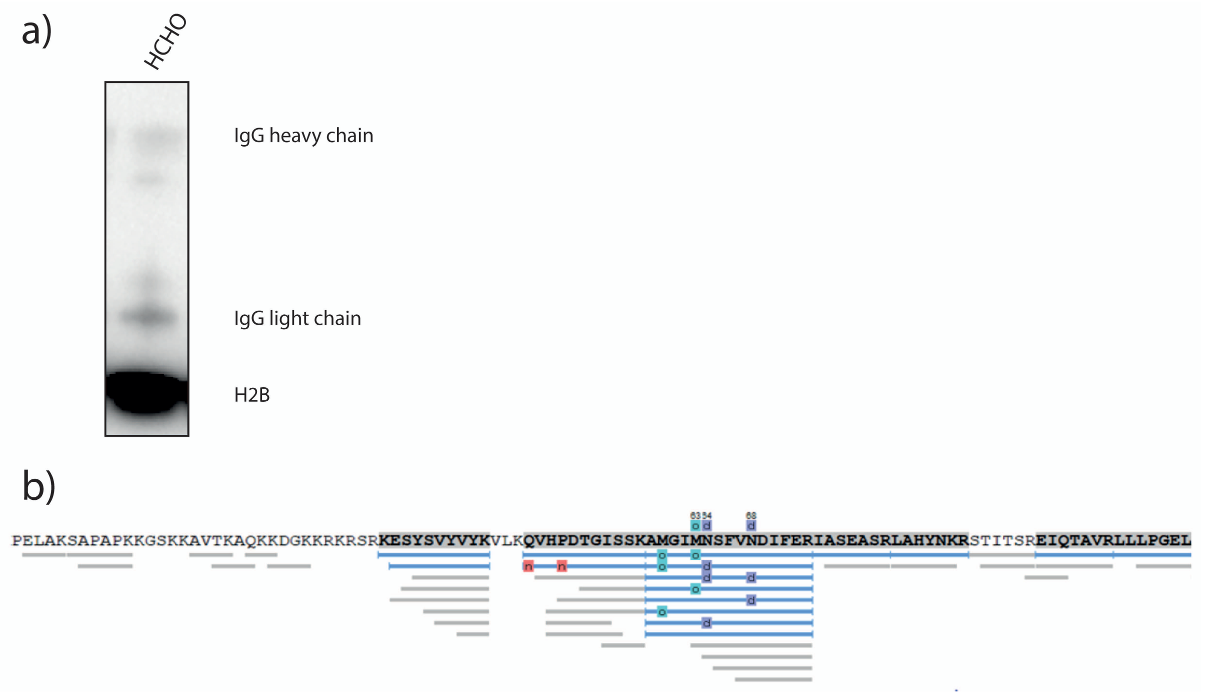

Figure S25| MS/MS analyses of immunoprecipitated H2B. a) HEK293T cells were treated with 100 µM HCHO for 16 h, followed by addition of 300 µM HCHO before being lysed 4 h after that. Histone H2B protein was then immunoprecipitated from the lysate and the presence of H2B was confirmed by western blot analysis. **b)** Tryptic peptides of immunoprecipitated H2B were subjected to LTQ Orbitrap Elite LC-MS/MS analysis. Coverage was not obtained for the N-terminal peptide fragments.

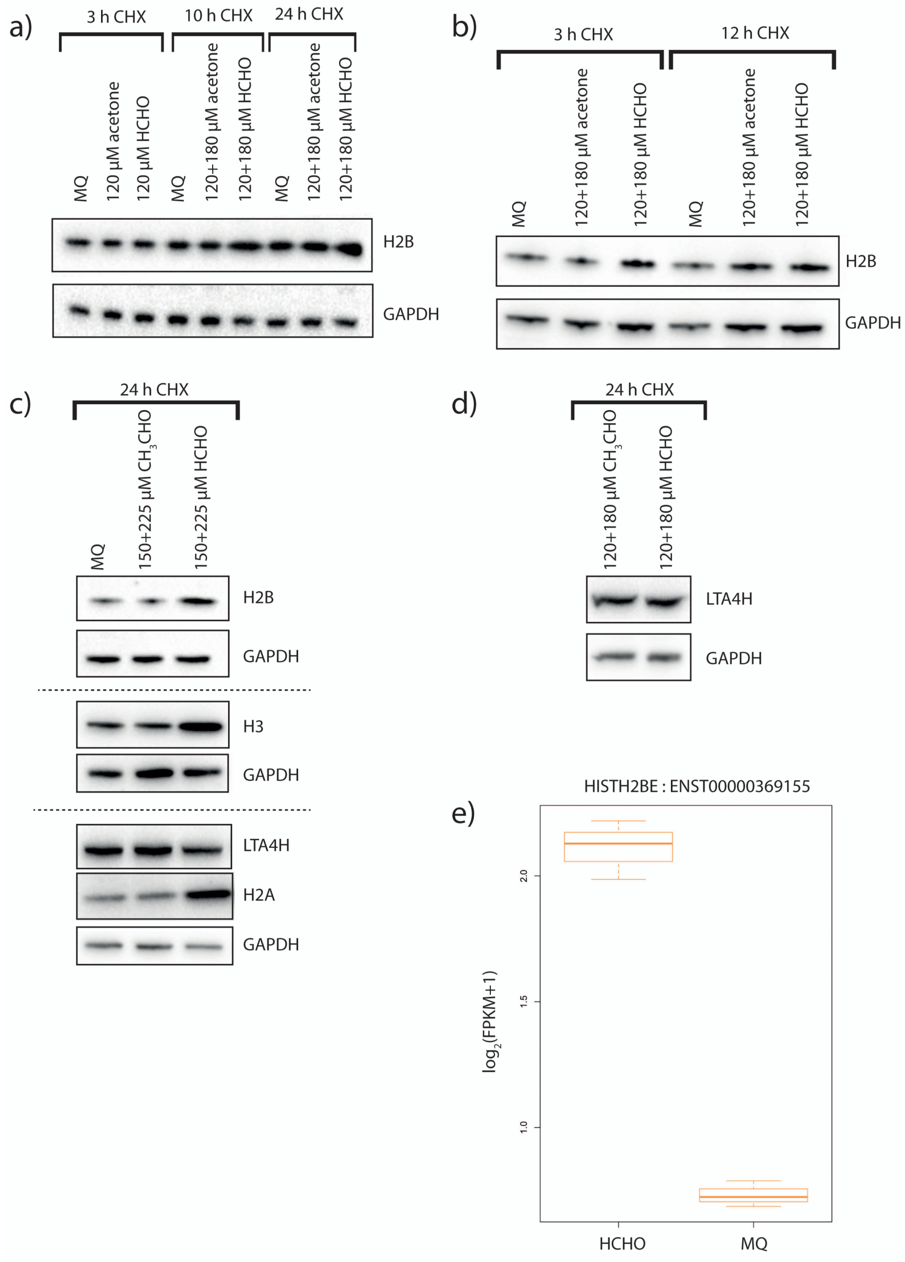

Figure S26 | Stability of proteins with an N-terminal proline in the presence of HCHO and RNA-Seq analyses. **a)** HEK293T cells were treated with 120 µM HCHO (or acetone, or MQ water control; 100 mM stock solutions of carbonyl compounds in MQ water) for 14 h. The cells were then treated with 100 µM cycloheximide (CHX; from a 100 mM DMSO stock solution) and were harvested after 3 h. Additionally, HEK293T cells were treated with 120 µM HCHO (or acetone, or MQ water control) for 14 h, followed by addition of CHX. 7.5 h after the CHX treatment, a further 180 µM of the carbonyl compound was added. The cells were harvested 10 h and 24 h after CHX addition. **b)** HEK293T cells were treated with MQ water/acetone/HCHO overnight, then treated with both the next dose of MQ water/acetone/HCHO and CHX; the cells were then harvested 3 h or 12 h after the second dose. **c+d)** HEK293T cells were treated with MQ water/acetone/HCHO overnight and later treated with both the next dose of MQ water/acetone/HCHO and with CHX; cells were harvested 24 h after the second dose. The final concentration of CHX was 100 µM in all experiments. **e)** H2B transcript levels in HEK293T cells treated with HCHO (150+225 µM) or untreated cells. Errors: SD of the mean (n=3).

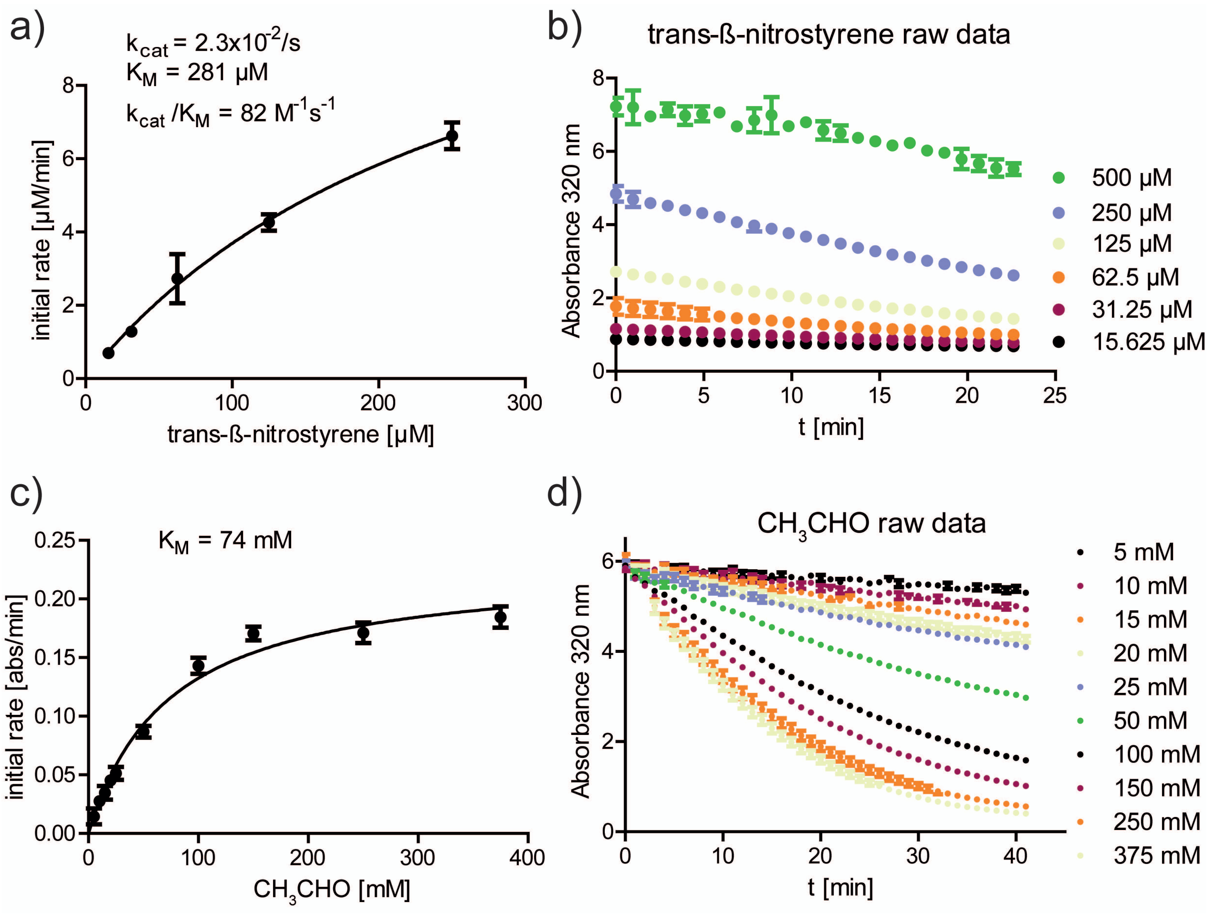

Figure S27 | Kinetic parameters of 4-OT. a) K_M for *trans*-β-nitrostyrene: 281 µM ± 42. **b)** Data used to determine the *trans*-β-nitrostyrene K_M. **c)** K_M for acetaldehyde (CH_3CHO): 74 mM ± 4.8. **d)** Data used to determine the CH_3CHO apparent K_M.

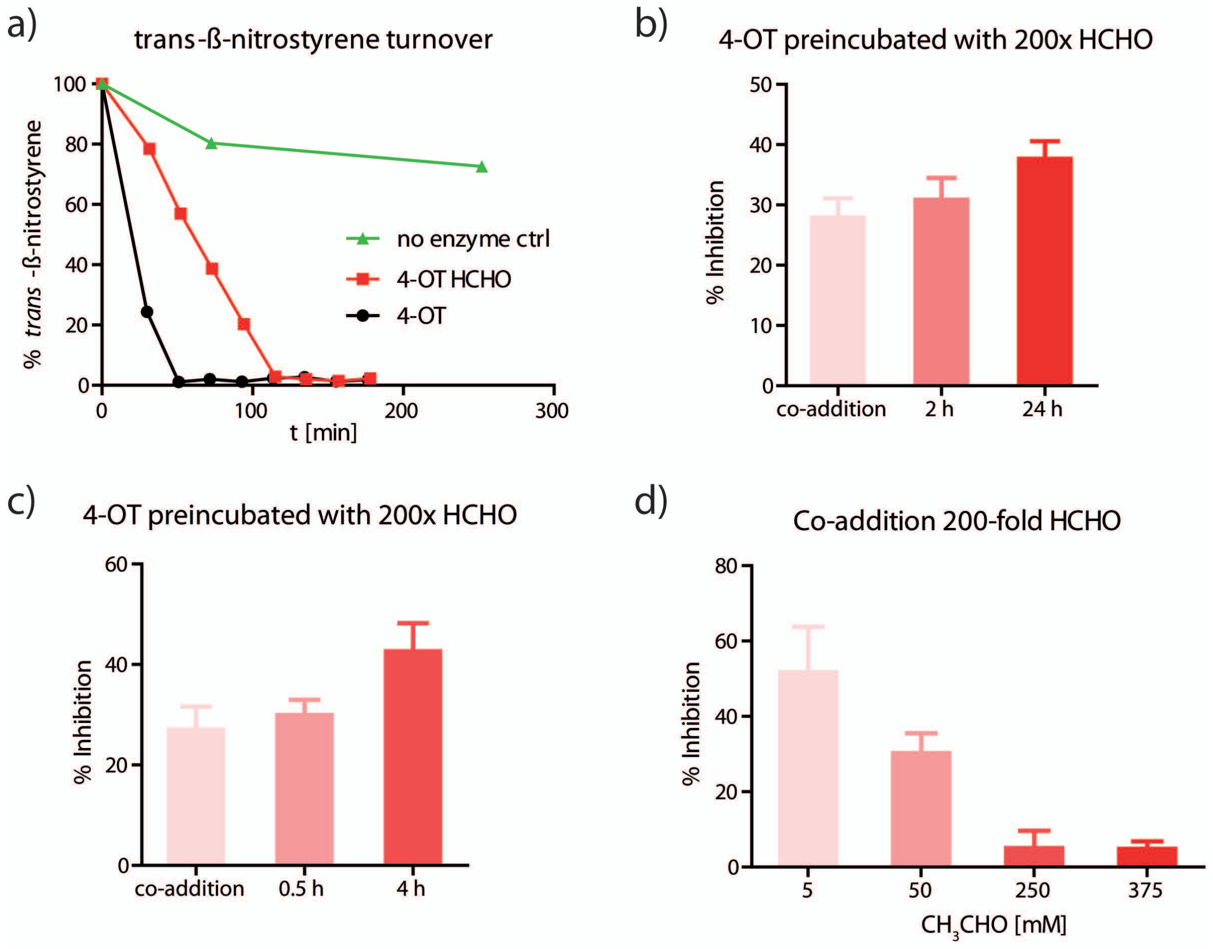

Figure S28 | HCHO inhibits 4-OT catalysis *in vitro*. Independent replicate of **Figure 4b+c**, employing a 200-fold excess of HCHO (or buffer control) for 17 h and 37 °C. Note non-enzymatic depletion of *trans*-β-nitrostyrene (no enzyme control) was observed. ^{1}H NMR (700 MHz) signals highlighted with a blue and green asterisk in **Figure 4b+c** were used to generate the graph. **b+c)** Results of an absorbance assay monitoring the depletion of *trans*-β-nitrostyrene at 320 nm (pH 7.4 (b), pH 7.8 (c)), showing a mild increase in inhibition correlates with prolonged pre-incubation time with HCHO. Errors: SD of the mean (n=2). **d)** A 200-fold molar excess of HCHO was added to 4-OT, immediately followed (no pre-incubation with HCHO) by addition of the indicated amounts of CH_3CHO and 200 µM *trans*-β-nitrostyrene at pH 7.4.

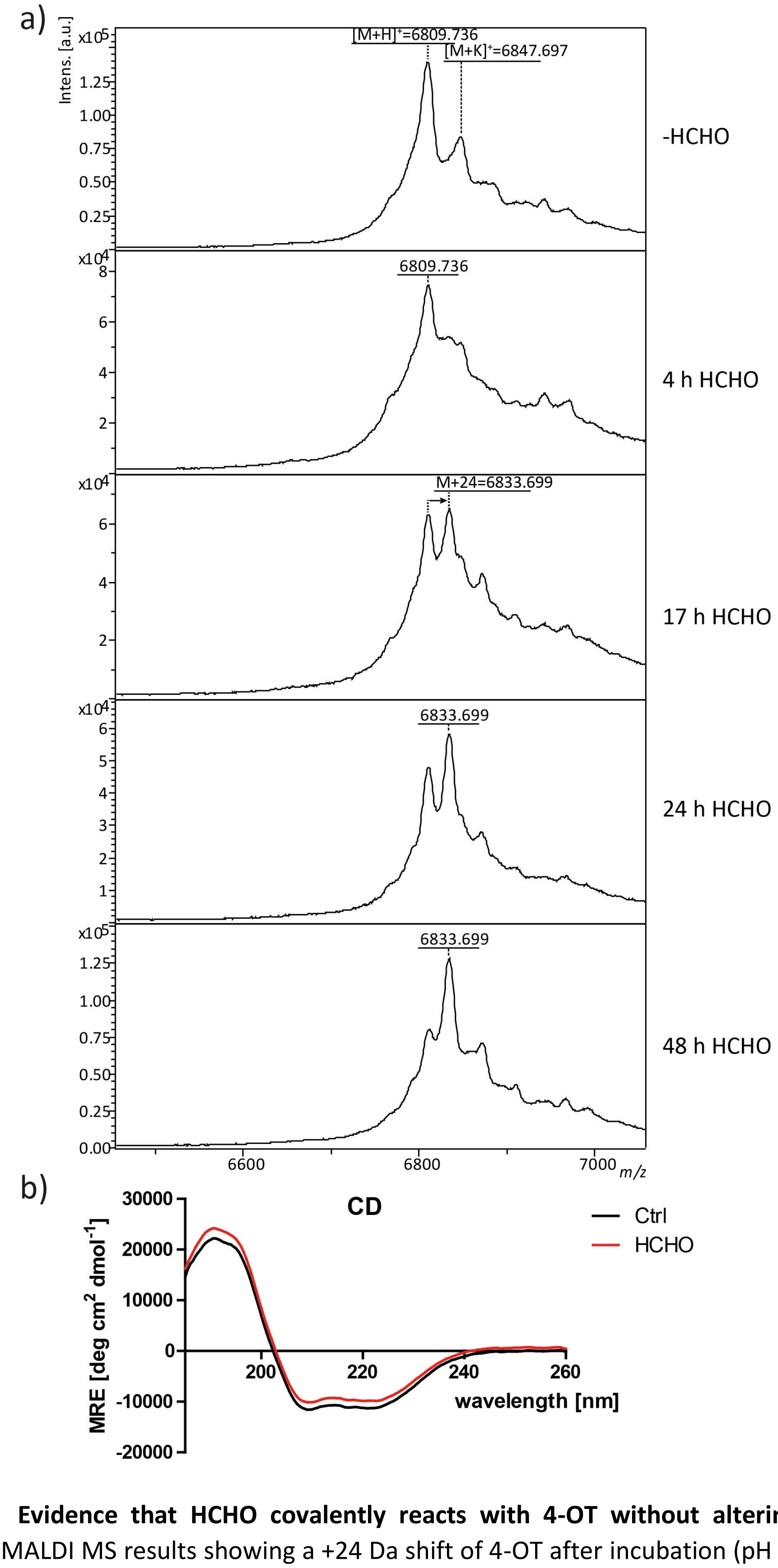

Figure S29 | Evidence that HCHO covalently reacts with 4-OT without altering its secondary structure. a) MALDI MS results showing a +24 Da shift of 4-OT after incubation (pH 7.8, 37 °C) with a 200-fold excess of HCHO. Similar results were obtained at pH 7.4 (data not shown). **b)** Circular dichroism spectra of 4-OT pre-incubated with 200-fold excess HCHO for 48 h (37 °C), compared with 4-OT treated with a buffer only control.

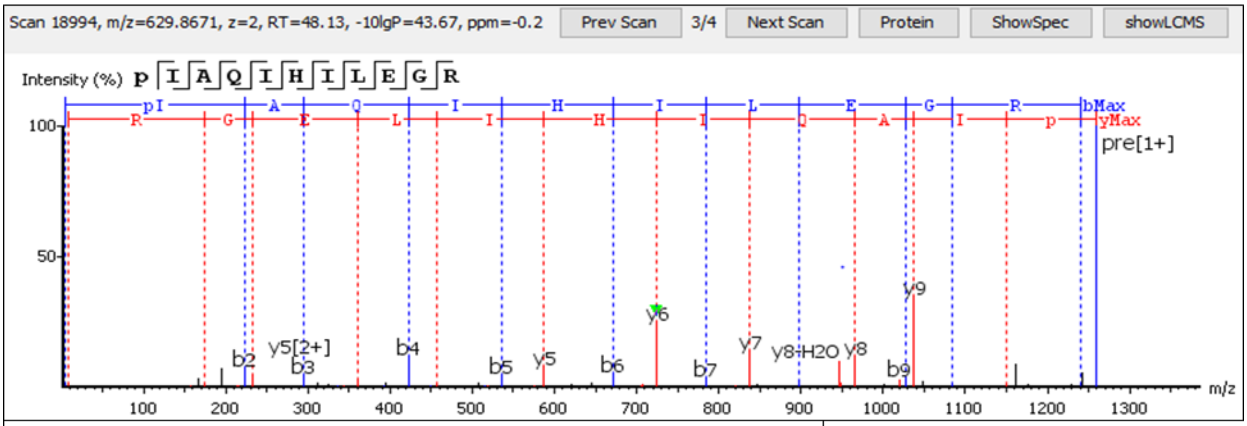

Ion Match | Survey

#	b	b-H2O	b-NH3	b (2+)	Seq	y	y-H2O	y-NH3	y (2+)	#
1	110.06	92.05	93.03	55.53	P(+12.00)					11
2	223.14	205.13	206.12	112.07	I	1149.67	1131.66	1132.65	575.33	10
3	294.18	276.17	277.15	147.59	A	1036.59	1018.58	1019.56	518.80	9
4	422.24	404.23	405.21	211.62	Q	965.55	947.54	948.53	483.28	8
5	535.32	517.31	518.30	268.16	I	837.49	819.48	820.46	419.25	7
6	672.38	654.37	655.36	336.70	H	724.41	706.41	707.38	362.71	6
7	785.47	767.46	768.44	393.24	I	587.35	569.34	570.32	294.18	5
8	898.55	880.54	881.52	449.78	L	474.27	456.26	457.24	237.63	4
9	1027.59	1009.58	1010.57	514.30	E	361.18	343.17	344.16	181.09	3
10	1084.61	1066.60	1067.59	542.81	G	232.14	214.15	215.11	116.57	2
11					R	175.12	157.11	158.09	88.06	1

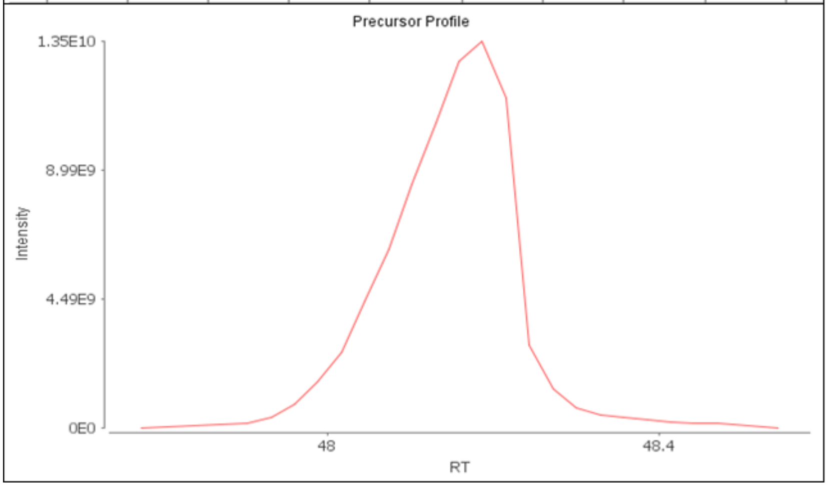

Figure S30| Evidence that recombinant 4-OT reacts with HCHO to form an N-terminal +12 Da adduct. 4-OT was incubated with HCHO (200-fold excess, 37 °C, 16 h in 20 mM potassium phosphate, pH 7.4) before being subjected to trypsin digestion and LUMOS LC-MS/MS analyses. Relevant b ions (blue) bearing the N-terminal +12 Da shift indicate the presence of the HCHO derived modification on the N-terminal proline of 4-OT (e.g. the b_2-type peptide ion Pro-Ile: *m/z* 223.14).

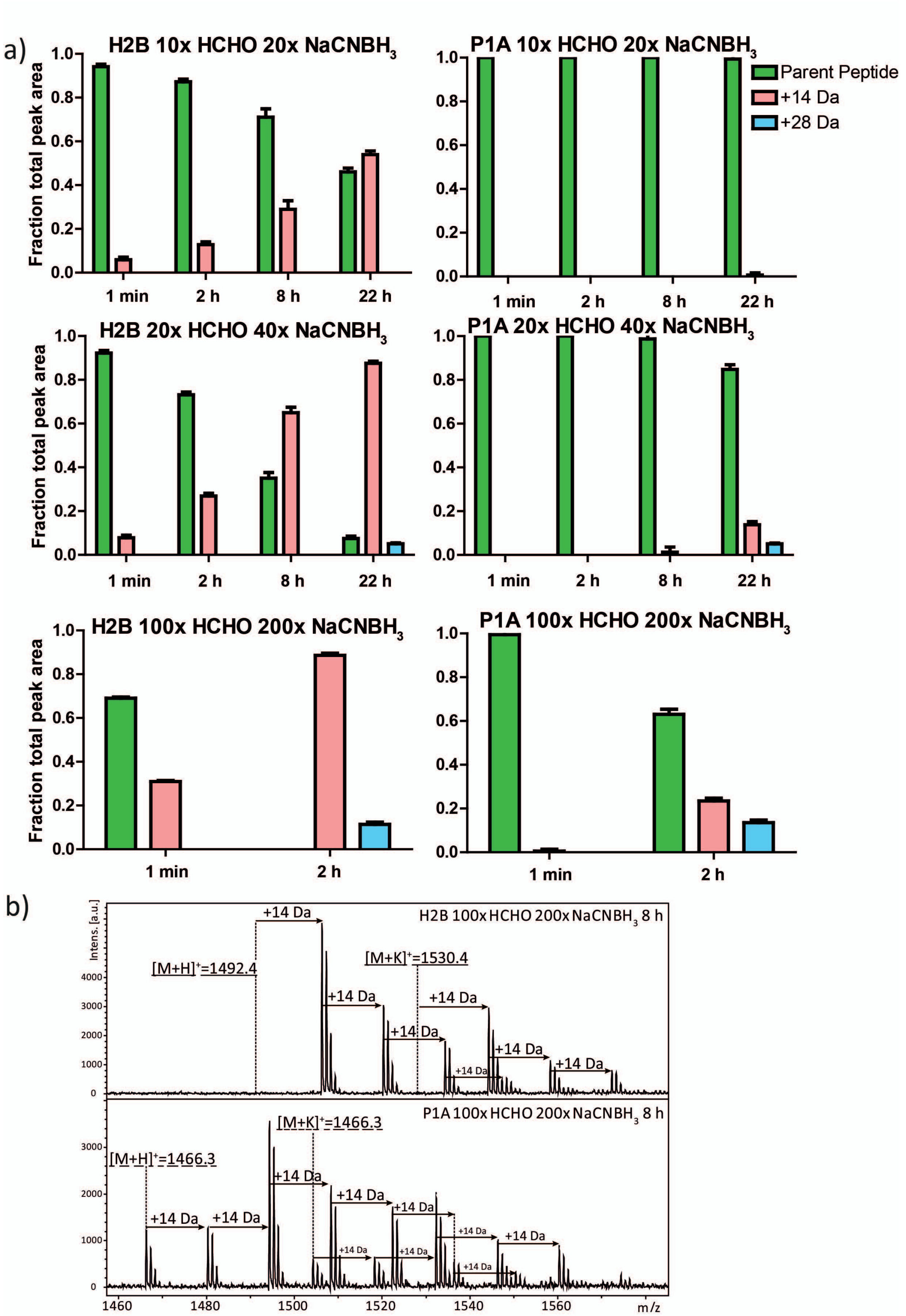

Figure S31 | Reductive methylation of the N-terminal proline of H2B. a) The H2B 15mer peptide or its P1A analogue were reacted with the indicated excesses of HCHO and $NaCNBH_3$ in 20 mM potassium phosphate, pH 7.4, ambient temperature. The reaction was monitored by MALDI MS. Errors: SD of the mean (n=3, technical repeats). **b)** Reaction of H2B 15mer or its P1A analogue using a 100-fold excess HCHO and 200-fold excess $NaCNBH_3$ (relative to peptide) results in abundant multiple +14 Da adducts after 8 h.

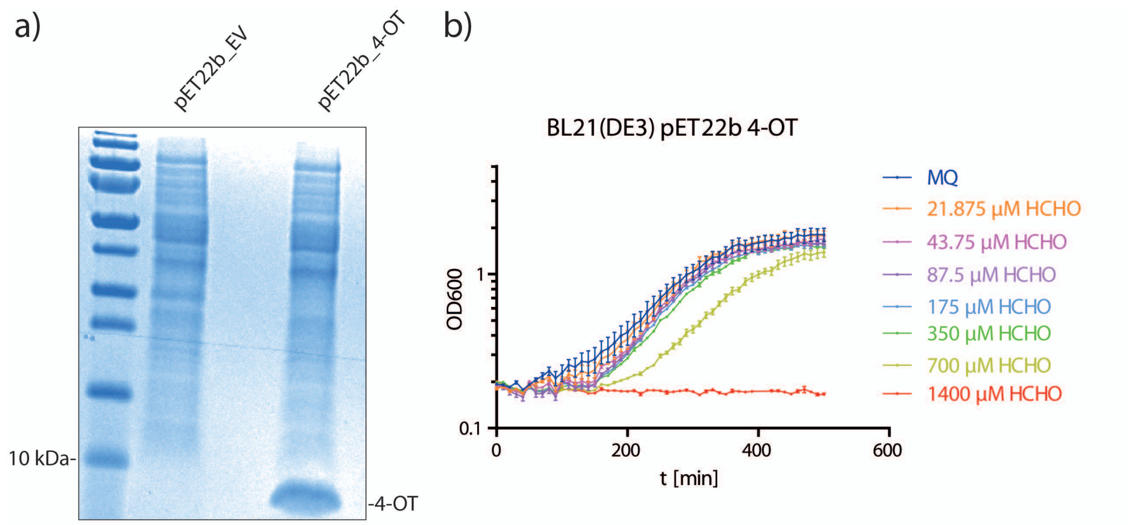

Figure S32 | Recombinant 4-OT production and the effect of HCHO on BL21(DE3) cell growth. a) SDS-PAGE gel of whole cell lysates (**Figure 4d+e**) confirming the overexpression of 4-OT at the beginning of the whole-cell assay. **b)** Growth curve showing the effects of HCHO on BL21(DE3) cell growth. Errors: SEM (n=3 technical repeats).

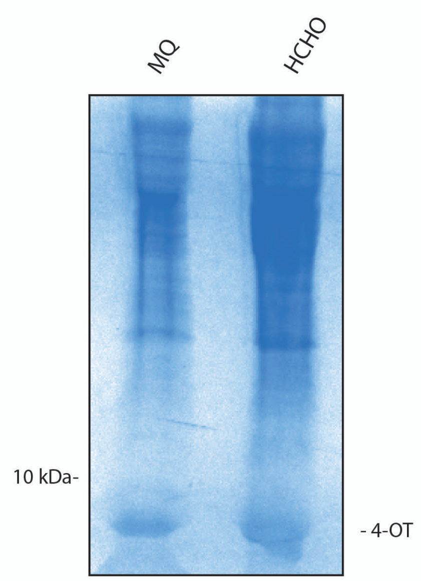

Figure S33 | In-gel trypsin digestion of 4-OT. Following re-inoculation using an overnight culture, 4-OT was biosynthesised for 24 h in BL21(DE3) (37 °C and shaking at 200 rpm, no IPTG added). After 24 h, 700 µM HCHO or MQ water were added and incubated (270 min, 37 °C and shaking at 200 rpm). Cells were centrifuged, sonicated and the supernatants containing the proteins were resolved by tricine SDS-PAGE. The bands corresponding to 4-OT were excised and subjected to trypsin digestion.

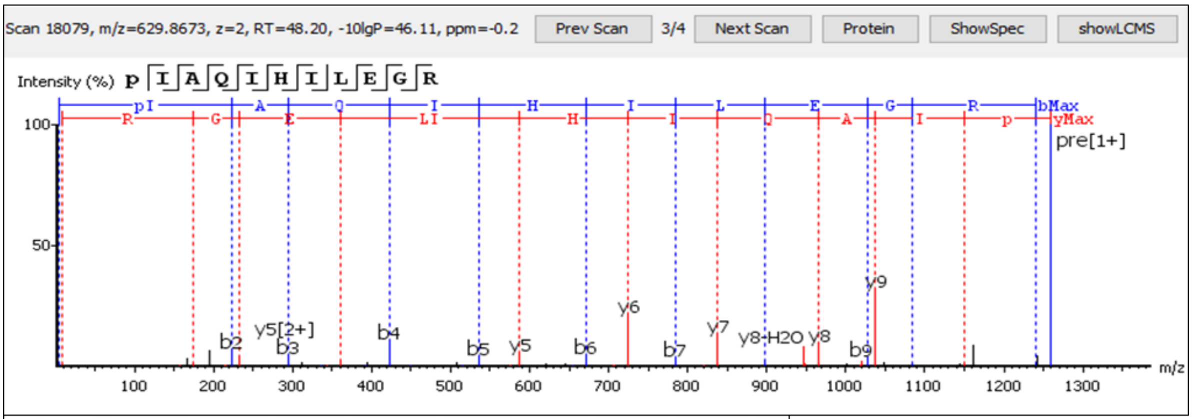

#	b	b-H2O	b-NH3	b (2+)	Seq	y	y-H2O	y-NH3	y (2+)	#
1	110.06	92.05	93.03	55.53	P(+12.00)					11
2	223.14	205.13	206.12	112.07	I	1149.67	1131.66	1132.65	575.33	10
3	294.18	276.17	277.15	147.59	A	1036.59	1018.57	1019.56	518.79	9
4	422.24	404.23	405.21	211.62	Q	965.55	947.53	948.53	483.28	8
5	535.32	517.31	518.30	268.16	I	837.49	819.48	820.47	419.25	7
6	672.38	654.37	655.36	336.70	H	724.41	706.40	707.39	362.71	6
7	785.47	767.46	768.44	393.24	I	587.35	569.34	570.32	294.18	5
8	898.55	880.54	881.52	449.78	L	474.27	456.26	457.24	237.63	4
9	1027.59	1009.58	1010.57	514.30	E	361.18	343.17	344.16	181.09	3
10	1084.61	1066.60	1067.59	542.81	G	232.14	214.15	215.11	116.57	2
11					R	175.12	157.11	158.09	88.06	1

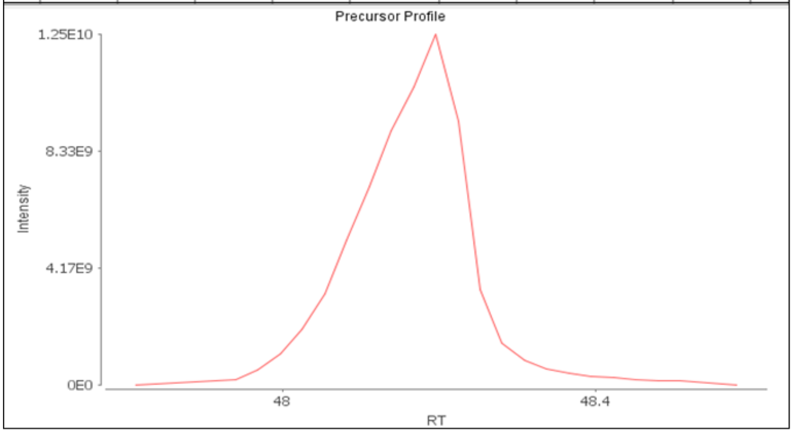

Figure S34 | Evidence that HCHO reacts with the N-terminal proline of 4-OT in *E. coli* cells. *E. coli* cells producing recombinant 4-OT were treated for 4.5 h with 700 µM HCHO. Proteins were resolved by tricine SDS-PAGE (**Figure S33**), bands corresponding to 4-OT were excised and subjected to trypsin digestion. The spectra obtained by LUMOS LC-MS/MS support the presence of an N-terminal +12 Da adduct. Relevant b ions (blue) bearing the N-terminal +12 Da shift indicate the presence of the HCHO derived modification on the N-terminal proline of 4-OT (e.g. the b_2-type peptide ion Pro-Ile: *m/z* 223.14).

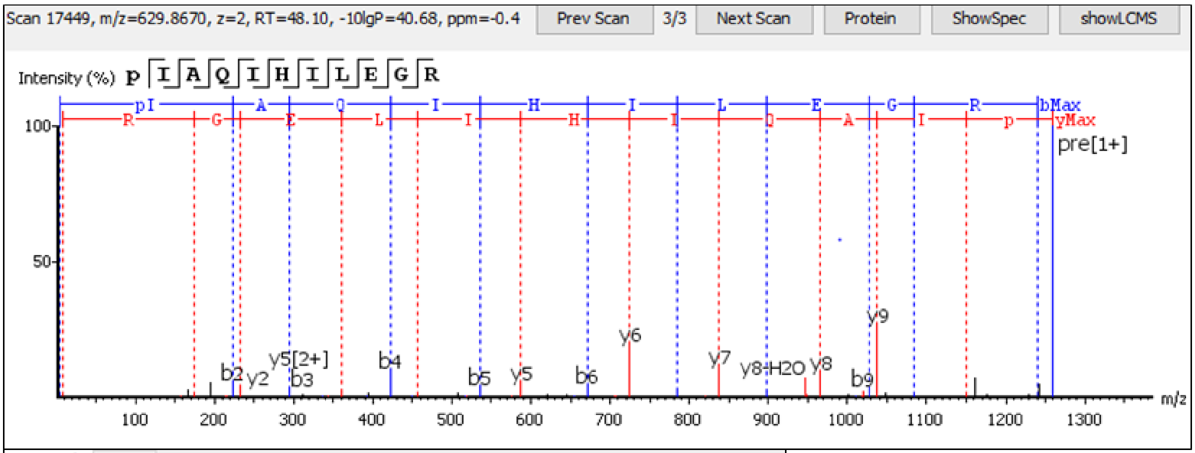

Ion Match | Survey

#	b	b-H2O	b-NH3	b (2+)	Seq	y	y-H2O	y-NH3	y (2+)	#
1	110.06	92.05	93.03	55.53	P(+12.00)					11
2	223.14	205.13	206.12	112.07	I	1149.67	1131.66	1132.65	575.33	10
3	294.18	276.17	277.15	147.59	A	1036.59	1018.57	1019.56	518.80	9
4	422.24	404.23	405.21	211.62	Q	965.55	947.54	948.53	483.28	8
5	535.32	517.31	518.30	268.16	I	837.49	819.48	820.47	419.25	7
6	672.38	654.37	655.36	336.70	H	724.41	706.40	707.38	362.71	6
7	785.47	767.46	768.44	393.24	I	587.35	569.34	570.32	294.18	5
8	898.55	880.54	881.52	449.78	L	474.27	456.26	457.24	237.63	4
9	1027.59	1009.58	1010.56	514.30	E	361.18	343.17	344.16	181.09	3
10	1084.61	1066.60	1067.59	542.81	G	232.14	214.16	215.11	116.57	2
11					R	175.12	157.11	158.09	88.06	1

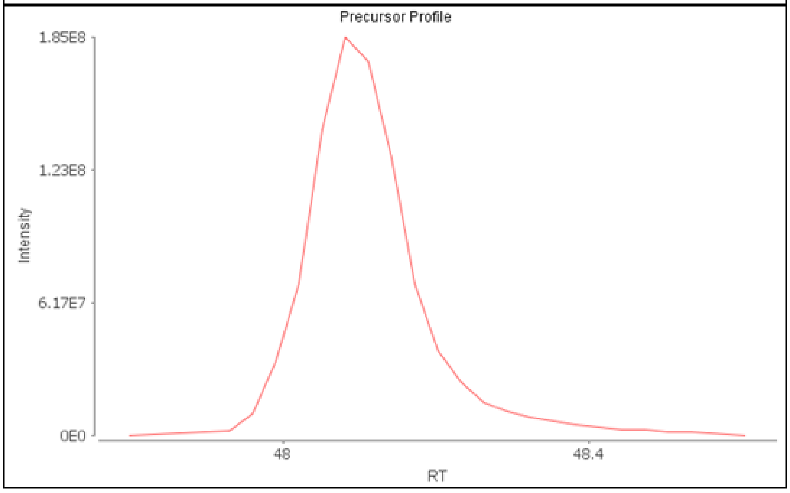

Figure S35 | Control experiment for the reaction of 4-OT with HCHO in cells. *E. coli* cells overexpressing 4-OT were treated for 4.5 h with MQ water control. Proteins were resolved by tricine SDS-PAGE (**Figure S33**); bands corresponding to 4-OT were excised, subjected to trypsin digestion and analysed by LUMOS LC-MS/MS. The intensity of the precursor profile shown is about 70 times less compared to the spectrum shown in **Figure S34**, indicating the +12 Da adduct is likely not present in this control sample.

Supplementary Methods

Solid-Phase Peptide Synthesis

Peptides were synthesised on a 0.1 mmol scale as C-terminal amides using a rink amide-MBHA resin (100–200 mesh, 0.6–0.8 mmol/g loading, AGTC Bioproducts) using a LibertyBlue microwave peptide synthesiser (CEM) as described.[3] *N*-α-Fmoc-protected amino acids (CS Bio, Novabiochem, Sigma-Aldrich, TCI, Alfa Aesar, Merck or AGTC Bioproducts) and, if required, acid-labile protecting groups were employed. Coupling and deprotection steps used the instrument's standard methods and were microwave assisted.

Amino acid solutions (0.2 M), dissolved in DMF (peptide synthesis grade, AGTC Bioproducts), were prepared. *N*,*N*'-Diisopropylcarbodiimide (TCI Europe) was used for coupling with Oxyma Pure. For Fmoc deprotection, 20% (v/v) piperidine in DMF was used. Upon synthesis of the peptide, including the final deprotection step, the resin was washed three times with CH_2Cl_2 and dried in air. Acid-labile protecting groups were removed and cleavage from the resin was conducted using 5 mL of a deprotection mixture (1,3-dimethoxybenzene (2.5%), triisopropylsilane (2.5%), Milli-Q (MQ) water (2.5%) in CF_3CO_2H (92.5%), v/v) for 4 h at ambient temperature. Upon filtration of the resulting mixture, the peptide was precipitated with ice-cold Et_2O (45 mL). The solid was pelleted (4255 g, 10 min, 4.0 °C) and the liquid was decanted and the solid dried in air. Before purification, peptides were dissolved in H_2O and lyophilised to remove CF_3CO_2H. Peptides were dissolved in H_2O and, if necessary, with acetonitrile (MeCN) to aid solubility. Upon filtration (0.45 µm), HPLC purification was conducted using a Shimadzu HPLC purification system (composed of DGU-20A, 2 LC-20AR, CBM-20A, SPD-20A, and FRC-10A units) equipped with a NX-C18 LC column (250 × 21.2 mm, 110 Å; Phenomenex Gemini). The mobile phase employed a gradient of MeCN + 0.1% (v/v) formic acid in H_2O + 0.1% (v/v) formic acid. Upon HPLC purification, MALDI MS or LC/MS were used to analyse fractions. Fractions containing the product were pooled according to purity and lyophilised. The purified peptide was dissolved in H_2O and its concentration was determined using quantitative ^{1}H NMR spectroscopy[4] (700 MHz): 16 µL of the D_2O peptide solution were added to 143 µL D_2O and 1 µL of 1 mg/mL 3-(trimethylsilyl)propionic-2,2,3,3-d_4 acid sodium salt (TSP; Apollo Scientific). The peptide concentration was calculated using the following equation: $M_x/M_y = (I_x/I_y) \times (N_y/N_x)$, with M_x/M_y representing the molar ratio of TSP (compound x) and the peptide (compound y), I_x/I_y the signal intensities corresponding to the methyl protons of TSP and the peptide (typically averaged over the Ala, Val, Thr, and Leu residues using Global Spectrum Deconvolution), and N_y/N_x the number of nuclei responsible for the intensity. The following N-terminal peptides were made as C-terminal amides, corresponding to the human sequences: H2B 1-15 (NH-PEPAKSAPAPKKGSK-NH_2); H2B P1A 1-15 (NH_2-AEPAKSAPAPKKGSK-NH_2); H2B 1-5 (NH-PEPAK-NH_2); H2A 1-15 (NH_2-SGRGKQGGKARAKAK-NH_2); H3 1-15 (NH_2-ARTKQTARKSTGGKA-NH_2); H3K9Me$_3$ 1-21 (NH_2-ARTKQTARK(Me_3)STGGKAPRKQLA-NH_2); H4 1-15 (NH_2-SGRGKGGKGLGKGGA-NH_2).

Dipeptide Synthesis

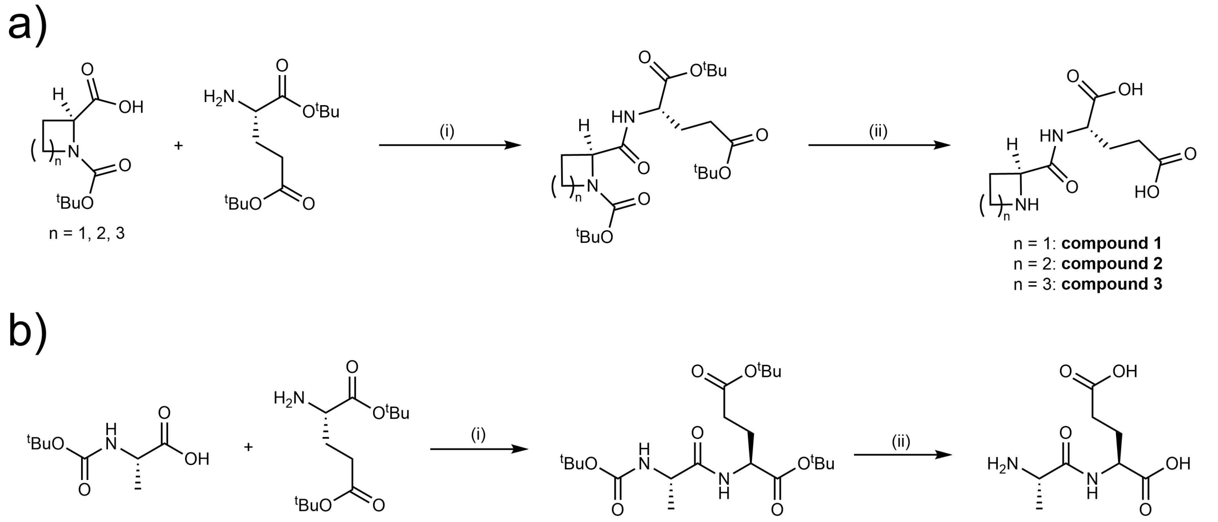

Figure S36 | Scheme for synthesis of dipeptides. a) Scheme for the synthesis of peptides containing an N-terminal 4-, 5-, or 6-membered ring (**1**, **2**, **3**). **b)** Scheme for the synthesis of the AlaGlu dipeptide (**4**). (i) 1-Ethyl-3-(3′-dimethylaminopropyl)carbodiimide (EDC), *N*-methylmorpholine, CH_2Cl_2, 0 °C to ambient temperature, 2 h ambient temperature. (ii) 50 equivalents of CF_3CO_2H in CH_2Cl_2.

a) Boc- and tert-butyl-protected Dipeptides

L-Glutamic acid di-tert-butyl ester hydrochloride (1.24 mmol) and the Boc-protected amino acid (Boc-*L*-alanine, Boc-*L*-proline, Boc-*L*-azetidine, Boc-*L*-piperidine; 1.24 mmol) were dissolved in 10 mL of CH_2Cl_2 at 0 °C. *N*-Methylmorpholine (1.24 mmol) and 1-ethyl-3-carbodiimide hydrochloride (1.24 mmol) were added and the mixture was allowed to warm to ambient temperature, then stirred for 1 h. Upon completion of the reaction, the mixture was quenched with 1 M HCl (aq) to pH 7, the organic layer was separated and washed with 1 M NaCl, dried over $MgSO_4$ and the solvent removed. Flash column chromatography (0-20% MeOH in CH_2Cl_2 (v/v) over 15 CV in a 10 g SiO_2 column) afforded the target compound (291 mg, 51%, 0.64 mmol for the *L*-Pro-*L*-Glu derivative; 213 mg, 36%, 0.45 mmol for the *L*-piperidine-*L*-Glu derivative; 300 mg, 56%, 0.7 mmol for the *L*-Ala-*L*-Glu derivative; 312 mg, 57%, 0.71 mmol for the *L*-azetidine- *L*-Glu derivative).

Boc-*L*-Ala-*L*-Glu(di-tBu-ester)

^{1}H NMR (400 MHz, $CDCl_3$) δ_H = 6.72 (d, *J*=7.9, 1H, 2), 5.16 – 4.99 (m, 1H, 22), 4.47 – 4.39 (m, 1H, 4), 4.21 – 4.06 (m, 1H, 21), 2.36 – 2.14 (m, 2H, 7), 2.15 – 2.00 (m, 1H, 6''), 1.98 – 1.80 (m, 1H, 6'), 1.44 (s, 9H, 27, 28, 29), 1.42 (s, 9H, 11, 12, 13), 1.41 (s, 9H, 18, 19, 20), 1.33 (d, *J*=7.1, 3H, 23); ^{13}C NMR (101 MHz, $CDCl_3$) δ_C = 172.5 (C1), 172.3 (C8), 170.9 (C5), 155.5 (C24), 82.4 (C17), 80.8 (C10), 80.1 (C26), 53.5 (C4), 52.3 (C21), 31.5 (C7), 28.4 (C18, C19, C20), 28.2 (C27, C28, C29), 28.1 (C11, C12, C13), 27.7 (C6), 18.5 (C23); ESI-MS, $[M+H]^+$: 431.28 (calculated), 453.20 (observed, obtained as Na^+ adduct); HRMS (ESI+) $(M+H)^+$: 431.27518 (calculated for $C_{21}H_{39}N_2O_7$), 431.27505 (observed).

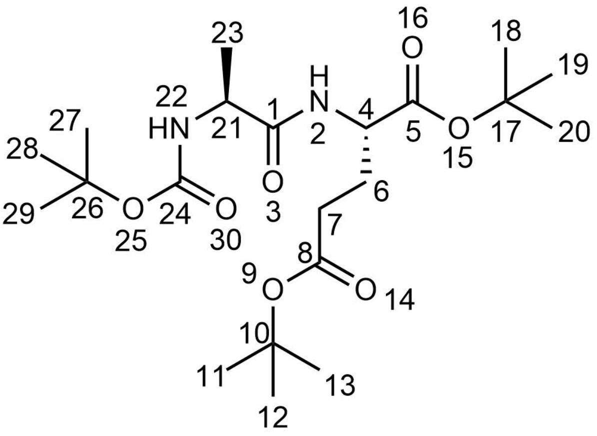

Boc-*L*-Pro-*L*-Glu(di-tBu-ester)

^{1}H NMR (400 MHz, $CDCl_3$) δ_H = 6.66 (s, 1H, 2), 4.43 (s, 1H, 4), 4.23 (d, *J*=29.8, 1H, 21), 3.40 (d, *J*=55.4, 2H, 24), 2.35 – 2.14 (m, 3H, 7, 22''), 2.14 – 2.02 (m, 1H, 6''), 1.92 – 1.80 (m, 2H, 6', 22', 23), 1.45 (s, 18H, 11, 12, 13, 29, 30, 31), 1.42 (s, 9H, 18, 19, 20); ^{13}C NMR (101 MHz, $CDCl_3$) δ_C = 172.2 (C1, C5, C8), 171.0 (C26), 82.3 (C17), 80.8 (C28), 77.4 (C10), 60.1 (C21), 52.0 (C4), 47.1 (C24), 31.5 (C7), 31.2 (C22), 28.4 (C11, C12, C13), 28.2 (C18, C19, C20), 28.1 (C29, C30, C31), 24.8 (C6), 23.9 (C23); ESI-MS, $[M+H]^+$: 457.29 (calculated), 479.20 (observed, obtained as Na^+ adduct); HRMS (ESI+) $(M+H)^+$: 457.29083 (calculated for $C_{23}H_{41}N_2O_7$), 457.29023 (observed).

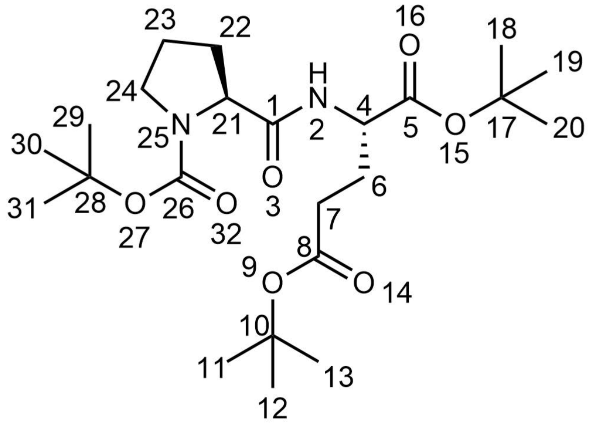

Boc-*L*-Piperidine-*L*-Glu(di-tBu-ester)

^{1}H NMR (500 MHz, $CDCl_3$) δ_H = 6.66 (d, *J*=66.1, 1H, 15), 4.73 (d, *J*=35.6, 1H, 4), 4.45 (d, *J*=7.2, 1H, 17), 4.06 (d, *J*=82.7, 1H, 2'), 2.81 (s, 1H, 2''), 2.36 – 2.23 (m, 1H, 26), 2.20 (dd, *J*=16.1, 5.7, 1H), 2.16 – 2.05 (m, 1H, 25''), 1.93 – 1.81 (m, 1H, 25'), 1.68 – 1.50 (m, 4H, 1, 5'', 6''), 1.48 (s, 8H, 10, 11, 12), 1.46 (s, 2H, 6'), 1.45 (s, 8H, 31, 32, 33), 1.44 (s, 1H, 5'), 1.43 (s, 9H, 21, 22, 23); ^{13}C NMR (126 MHz, $CDCl_3$) δ_C = 172.1 (C18), 171.2 (C14), 170.9 (C27), 156.1 (C7), 82.4 (C20), 80.8 (C30), 80.7 (C9), 54.5 (C4), 52.3 (C17), 42.7 (C2), 31.7 (C26), 28.5 (C31, C32, C33), 28.2 (C21, C22, C23), 28.1 (C10, C11, C12), 27.7 (C25), 25.6 (C5), 25.0 (C1), 20.6 (C6); ESI-MS, $[M+H]^+$: 470.3 (calculated), 493.2 (observed, obtained as Na^+ adduct); HRMS (ESI+) $(M+H)^+$: 471.30648 (calculated for $C_{24}H_{43}N_2O_7$), 471.30673 (observed).

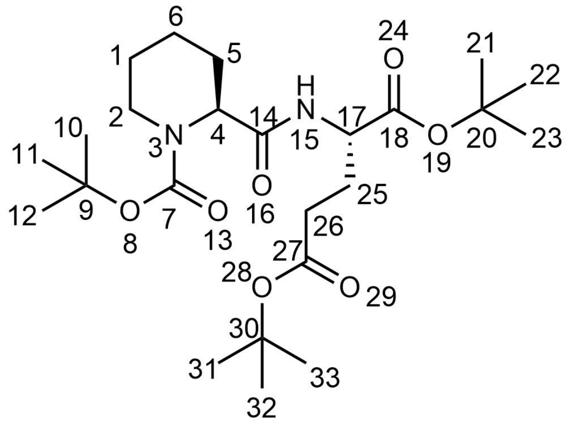

Boc-*L*-Azetidine-*L*-Glu(di-tBu-ester)

^{1}H NMR (500 MHz, $CDCl_3$) δ_H = 4.62 (t, *J*=7.9, 1H, 13), 4.47 (td, *J*=7.9, 5.1, 1H, 3), 3.90 (q, *J*=8.3, 1H, 15''), 3.80 (td, *J*=8.3, 6.3, 1H, 15'), 2.41 (s, 2H, 14), 2.34 – 2.20 (m, 2H, 9), 2.18 – 2.07 (m, 1H, 4''), 1.97 – 1.87 (m, 1H, 4'), 1.46 (s, 9H, 18, 19, 20), 1.45 (s, 9H, 22, 23, 24), 1.43 (s, 9H, 28, 29, 30); ^{13}C NMR (126 MHz, $CDCl_3$) δ_C = 172.1 (C1), 171.5 (C5), 170.7 (C10), 157.2 (C25), 82.2 (C17), 81.1 (C21), 80.7 (C27), 62.3 (C13), 52.0 (C3), 47.1 (C15), 31.5 (C9), 28.5 (C14), 28.3 (C18, C19, C20), 28.2 (C28, C29, C30), 28.1 (C22, C23, C24), 27.9 (C4); ESI-MS, $[M+H]^+$: 443.28 (calculated), 465.2 (observed, obtained as Na^+ adduct); HRMS (ESI+) $(M+H)^+$: 443.27518 (calculated for $C_{22}H_{39}N_2O_7$), 443.27518 (observed).

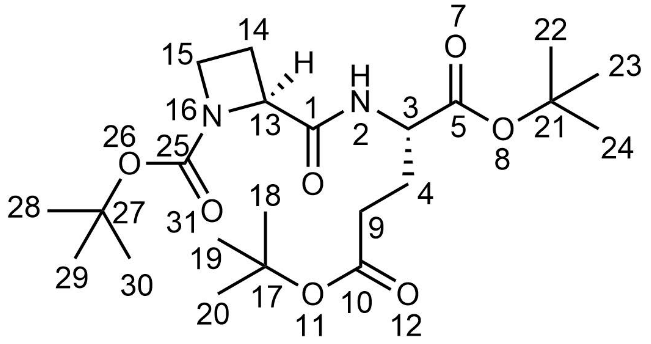

b) Deprotection of Dipeptides

The deprotection procedure was adapted from that of Polavckova et al.[5] The protected dipeptide was dissolved in CH_2Cl_2 and a 50-fold molar excess of CF_3CO_2H was added. The mixture was stirred at ambient temperature for 2 h. Upon completion of the reaction, the mixture was evaporated to dryness and the oily residue was evaporated three times with toluene. The oily residue was extracted with H_2O/ethyl acetate and the organic phase was washed with 1 M HCl. The aqueous layer was concentrated and purified by HPLC using a ACE5 C18 column, 100 x 21.2 mm employing a 2% isocratic elution at 2% MeCN in H_2O, 0.1% formic acid (v/v) over 12 min to afford the target compounds (118 mg, 76%, 0.48 mmol for *L*-proline-*L*-Glu (**2**); 133 mg, 81%, 0.58 mmol for *L*-azetidine-*L*-Glu (**1**); 102 mg, 88%, 0.39 mmol for *L*-piperidine-*L*-Glu (**3**); 116 mg, 76%, 0.53 mmol for *L*-alanine-*L*-Glu (**4**)).

***L*-Ala-*L*-Glu (compound 4)**

^{1}H NMR (400 MHz, MeOD) δ_H = 4.53 – 4.43 (m, 1H, 4), 3.99 (q, *J*=7.1, 1H, 13), 2.46 (dt, *J*=14.0, 7.5, 2H, 7), 2.29 – 2.16 (m, 1H, 6'), 2.05 – 1.90 (m, 1H, 6''), 1.54 (dd, *J*=7.1, 1.0, 3H, 15); ^{13}C NMR (101 MHz, MeOD) δ_C = 176.3 (C8), 174.8 (C5), 171.2 (C1), 53.0 (C4), 50.1 (C13), 31.0 (C7), 27.7 (C6), 17.5 (C15); ESI-MS, $[M+H]^+$: 219.10 (calculated), 219.15 (observed); HRMS (ESI+) $(M+H)^+$: 219.09755 (calculated for $C_8H_{15}N_2O_5$), 219.09781 (observed).

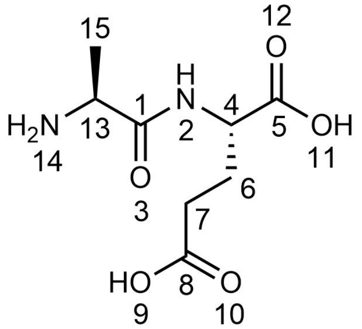

***L*-Piperidine-*L*-Glu (compound 3)**

^{1}H NMR (400 MHz, MeOD) δ_H = 4.49 (dd, *J*=9.2, 5.0, 1H, 10), 3.83 (dd, *J*=11.6, 3.3, 1H, 4), 3.44 – 3.36 (m, 1H, 2''), 3.08 – 2.96 (m, 1H, 2'), 2.43 (dd, *J*=8.0, 6.9, 2H, 15), 2.33 – 2.13 (m, 2H, 5', 14''), 2.06 – 1.83 (m, 3H, 1'', 6'', 14'), 1.81 – 1.59 (m, 3H, 1', 5'', 6'); ^{13}C NMR (101 MHz, MeOD) δ_C = 176.3 C(16), 174.3 (C11), 170.2 (C7), 59.0 (C4), 53.0 (C10), 44.9 C(2), 31.1 (C15), 28.5 (C5), 27.7 (C14), 23.0 (C1), 22.8 (C6); ESI-MS, $[M+H]^+$: 259.13 (calculated), 259.15 (observed); HRMS (ESI+) $(M+H)^+$: 259.12885 (calculated for $C_{11}H_{19}N_2O_5$), 259.12887 (observed).

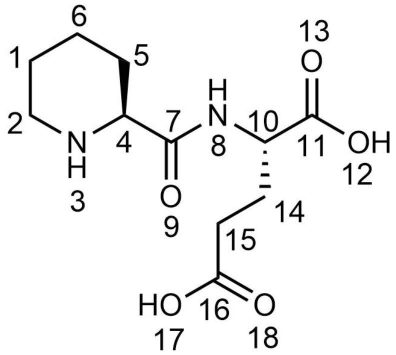

***L*-Pro-*L*-Glu (compound 2)**

^{1}H NMR (400 MHz, MeOD) δ_H = 4.48 (dd, *J*=9.2, 5.0, 1H, 4), 4.36 (dd, *J*=8.6, 6.3, 1H, 13), 3.47 – 3.33 (m, 2H, 16), 2.52 – 2.38 (m, 3H, 7, 14'), 2.30 – 2.17 (m, 1H, 6'), 2.16 – 1.91 (m, 4H, 6'', 14'', 15); ^{13}C NMR (101 MHz, MeOD) δ_C = 176.2 (C8), 174.3 (C5), 170.0 (C1), 61.0 (C13), 53.3 (C4), 47.5 (C16), 31.1 (C7), 30.9 (C14), 27.6 (C6), 24.9 (C15); ESI-MS, $[M+H]^+$: 245.11 (calculated), 245.00 (observed); HRMS (ESI+) $(M+H)^+$: 245.11320 (calculated for $C_{10}H_{17}N_2O_5$), 245.11315 (observed).

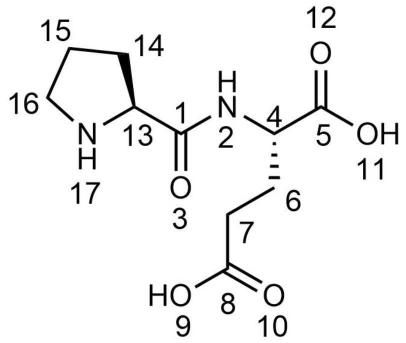

***L*-Azetidine-*L*-Glu (compound 1)**

^{1}H NMR (400 MHz, MeOD) δ_H = 5.04 (t, *J*=8.4, 1H, 4), 4.51 (dd, *J*=9.1, 5.0, 1H, 8), 4.12 (q, *J*=9.2, 1H, 2'), 4.02 – 3.92 (m, 1H, 2''), 2.94 – 2.77 (m, 1H, 1'), 2.69 – 2.51 (m, 1H, 1''), 2.43 (t, *J*=7.5, 2H, 11), 2.32 – 2.13 (m, 1H, 10'), 2.03 – 1.89 (m, 1H, 10''); ^{13}C NMR (101 MHz, MeOD) δ_C = 174.6 (C12), 173.1 (C9), 169.2 (C5), 59.9 (C4), 53.3 (C8), 45.2 (C2), 30.9 (C11), 27.5 (C10), 24.6 (C1); ESI-MS, $[M+H]^+$: 231.10 (calculated), 231.15 (observed); HRMS (ESI+) $(M+H)^+$: 231.09755 (calculated for $C_9H_{15}N_2O_5$), 231.09762 (observed).

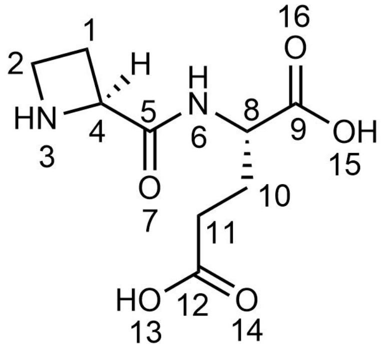

c) Dipeptide Reactions with HCHO

The deprotected dipeptide was then used to perform reactions with HCHO (12 h, ambient temperature, H_2O). In general, to obtain a HCHO solution, paraformaldehyde was resuspended in a microwave vial in the respective solvent (here H_2O) and heated at 95 °C for 3 h in a Biotage Initiator+ under stirring to obtain a colourless solution. 10-fold molar excess of aqueous HCHO (410 µmol) was added to the dipeptide (41 µmol) and NaOD was added to obtain a reaction mixture at pH 10 (pH 12 for **compound 4**). The solution was neutralized after 12 h and subjected to HPLC purification (isocratic elution, 2% MeCN in H_2O, 0.1% formic acid (v/v), 10 min, column ACE5 C18, 100 x 21.2 mm). The product was concentrated to give a colourless solid. Yields were 3 mg, 28.5%, 11.7 µmol for *L*-proline-*L*-Glu-HCHO-adduct; 7 mg, 63%, 25.9 µmol for *L*-piperidine-*L*-Glu-HCHO-adduct; 6.5 mg, 26%, 21.1 µmol for *L*-alanine-*L*-Glu-HCHO adduct; 5 mg for *L*-azetidine-*L*-Glu-HCHO-adduct which was present as ~50% hemiaminal (9.6 µmol, ~23% yield), and ~50% aminal (10.3 µmol, ~25% yield), the latter of which was observed to slowly degrade.

_L_-Alanine-_L_-Glu-HCHO adduct (compound 4a)

^{1}H NMR (400 MHz, DMSO) δ_H = 4.42 – 4.36 (m, 1H, 11''), 4.28 – 4.20 (m, 2H, 11', 17''), 4.13 (d, *J*=10.8, 1H, 17'), 4.07 (dd, *J*=11.5, 4.2, 1H, 2), 3.40 (q, *J*=6.8, 1H, 13), 2.12 – 1.98 (m, 1H, 4'), 1.90 – 1.80 (m, 2H, 5''), 1.67 – 1.55 (m, 1H, 4''), 1.10 (d, *J*=6.9, 3H, 16); ^{13}C NMR (101 MHz, DMSO) δ_C = 178.4 (C6), 175.4 (C3), 175.1 (C14), 75.4 (C17), 63.0 (C11), 56.70 (C13), 56.5 (C2), 35.3 (C5), 27.0 (C4), 15.9 (C16); ESI-MS, $[M+H]^+$: 261.10 (calculated) 231.10 (calculated for aminal-methylene bridge only, without hemiaminal), 231.10 (observed; hemiaminal group not stable under LC/MS conditions); HRMS (ESI-) $(M-H)^-$: 229.0830 (calculated for $C_9H_{13}N_2O_5^-$), 229.0829 (observed); hemiaminal not stable under HRMS conditions. Note a few drops of D_2O were added to the compound to aid solubility in DMSO for NMR analysis.

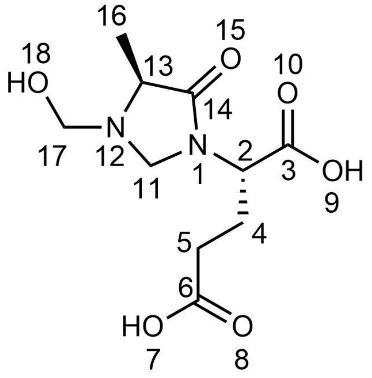

_L_-Piperidine-_L_-Glu-HCHO adduct (compound 3a)

^{1}H NMR (700 MHz, DMSO) δ_H = 4.49 (dd, *J*=11.0, 4.8, 1H, 2), 4.15 (d, *J*=5.6, 1H, 11''), 3.88 (dd, *J*=5.6, 2.0, 1H, 11'), 2.85 – 2.76 (m, 1H, 15''), 2.74 – 2.69 (m, 1H, 13), 2.42 – 2.35 (m, 1H, 15'), 2.27 – 2.15 (m, 2H, 7), 2.14 – 2.06 (m, 1H, 6'), 1.90 – 1.80 (m, 1H, 6''), 1.79 – 1.73 (m, 1H, 18''), 1.64 – 1.56 (m, 1H, 16''), 1.55 – 1.45 (m, 2H, 16', 17'), 1.44 – 1.36 (m, 1H, 18'), 1.36 – 1.28 (m, 1H, 17''); ^{13}C NMR (101 MHz, DMSO) δ_C = 173.4 (C8), 173.0 (C14), 171.7 (C3), 64.7 (C11), 62.6 (C13), 52.1 (C2), 48.9 (C15), 30.1 (C7), 24.3 (C6), 23.9 (C16), 23.6 (C18), 22.6 (C17); ESI-MS, $[M+H]^+$: 271.12 (calculated), 271.15 (observed); HRMS (ESI+) $(M+H)^+$: 271.1288 (calculated for $C_{12}H_{19}N_2O_5$), 271.1288 (observed).

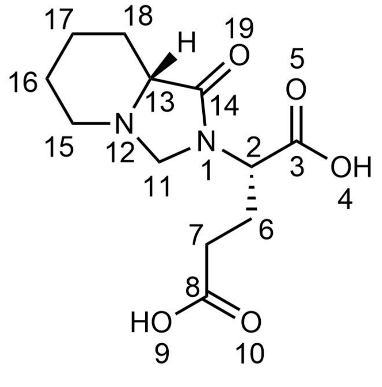

_L_-Proline-_L_-Glu-HCHO adduct (compound 2a)

^{1}H NMR (600 MHz, D_2O) δ_H = 5.27 (dd, *J*=8.9, 0.9, 1H, 8'), 4.84 – 4.83 (m, 4H, 1), 4.82 – 4.81 (m, 1H, 8''), 4.53 (dd, *J*=10.8, 4.8, 1H, 10), 3.96 – 3.84 (m, 1H, 3'), 3.56 – 3.45 (m, 1H, 3''), 2.47 – 2.41 (m, 3H, 5'', 15), 2.37 – 2.31 (m, 2H, 5', 14'), 2.20 – 2.13 (m, 2H, 4), 2.11 – 2.03 (m, 1H, 14''); ^{13}C NMR (151 MHz, D_2O) δ_C = 177.6 (C16), 174.5 (C11), 170.0 (C6), 66.0 (C1), 65.9 (C8), 57.9 (C3), 56.8 (C10), 31.6 (C15), 27.4 (C5), 24.1 (C4), 24.1 (C14); ESI-MS, $[M+H]^+$: 257.11 (calculated), 257.15 (observed); HRMS (ESI+) $(M+H)^+$: 257.1132 (calculated for $C_{11}H_{17}N_2O_5^+$), 257.1133 (observed).

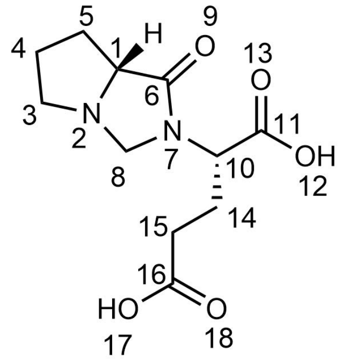

_L_-Azetidine-_L_-Glu-HCHO adduct - Aminal (compound 1a)

^{1}H NMR (400 MHz, DMSO) δ_H = 4.33 – 4.21 (m, 1H, 4), 4.09 – 3.91 (m, 1H, 15''), 3.76 (s, 1H, 13), 3.36 (q, *J*=7.1, 6.4, 1H, 17''), 3.31 (s, 1H), 3.14 – 3.03 (m, 1H, 17'), 2.32 – 2.12 (m, 3H, 7, 16''), 2.10 – 1.94 (m, 2H, 6', 16'), 1.92 – 1.78 (m, 1H, 6''); ^{13}C NMR (101 MHz, DMSO) δ_C = 173.7 (C8), 172.9 (C5), 163.0 (C1), 74.6 (C15), 63.9 (C13), 50.9 (C4), 50.7 (C17), 30.0 (C7), 26.7 (C6), 21.7 (C16); ESI-MS, $[M+H]^+$: 243.10 (calculated), 243.0 (observed); HRMS (ESI+) $(M+H)^+$: 243.0981 (calculated for $C_{10}H_{15}N_2O_5^+$), 243.0984 (observed).

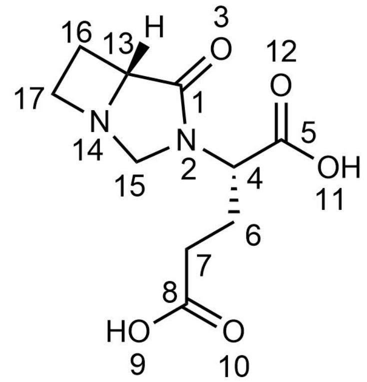

_L_-Azetidine-_L_-Glu-HCHO adduct – Hemiaminal (compound 1b)

^{1}H NMR (400 MHz, DMSO) δ_H = 4.31 – 4.18 (m, 1H, 4), 3.67 (q, *J*=7.4, 6.6, 1H, 13), 3.36 – 3.30 (m, 1H, 17''), 3.25 (s, 1H, 18), 3.06 (q, *J*=8.0, 1H, 17'), 2.34 – 2.23 (m, 2H, 7), 2.23 – 2.12 (m, 1H, 16''), 2.11 – 1.96 (m, 2H, 6', 16'), 1.93 – 1.78 (m, 1H, 6''); ^{13}C NMR (101 MHz, DMSO) δ_C = 173.8 (C8), 173.0 (C5), 171.6 (C1), 79.8 (C18), 63.9 (C13), 50.9 (C4), 50.4 (C17), 30.1 (C7), 26.8 (C6), 21.8 (C16); ESI-MS, $[M+H]^+$: 261.10 (calculated), 243.0 (observed; hemiaminal group not stable under LC/MS conditions); HRMS (ESI-) $(M-H)^-$: 259.0936 (calculated for $C_{10}H_{15}N_2O_6^-$), 259.0928 (observed).

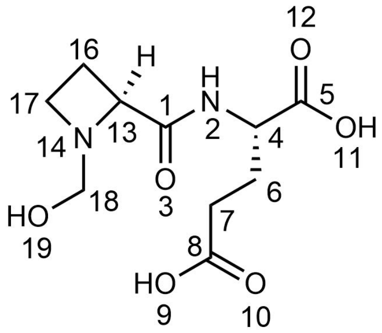

Reductive Methylation

The procedure for reductive methylation was modified from that of Metz et al.[6] The *L*-Pro-*L*-Glu dipeptide (82 µmol) was dissolved in H_2O, and HCHO (10 equivalents, 820 µmol) and $NaBH_3CN$ (3 equivalents, 246 µmol) were added. The mixture was basified to pH 9 with 1 M NaOH and stirred for 3 h at 40 °C. The mixture was then washed with Et_2O and $NaBH_3CN$ in the aqueous layer was quenched with NH_4Cl. The aqueous phase was purified by flash column chromatography with a solvent gradient of 0-50% (v/v) MeCN (0.5% formic acid) in H_2O (0.5% formic acid) over 15 column volumes in a 12 g Biotage SNAP KP-C18-HS column. The solvent was removed *in vacuo* to give a colourless solid, which necessitated further purification by HPLC. HPLC purification was conducted with H_2O and MeCN (with 0.1% CF_3CO_2H) using an elution gradient of 1-10% over 15 min, then 10-50% (v/v) over 8 min (Sunfire C18 5 µm 10 x 150 mm column). The product was concentrated to give a colourless solid. The yield was approximately 16 mg (76%, 62 µmol).

***N*-Methyl-*L*-Pro-*L*-Glu - Reductive Methylation**

^{1}H NMR (400 MHz, D_2O) δ_H = 4.50 (dd, *J*=9.0, 5.3, 1H, 4), 4.23 (dd, *J*=9.2, 7.6, 1H, 13), 3.86 – 3.72 (m, 1H, 16'), 3.31 – 3.19 (m, 1H, 16''), 2.96 (s, 3H, 18), 2.70 – 2.57 (m, 1H, 14''), 2.53 (t, *J*=7.2, 2H, 7), 2.32 – 2.20 (m, 2H, 6', 15'), 2.19 – 2.11 (m, 1H, 14'), 2.11 – 1.99 (m, 1H, 6'', 15''); ^{13}C NMR (101 MHz, D_2O) δ_C = 177.0 (C8), 174.5 (C5), 168.5 (C1), 68.6 (C13), 56.5 (C16), 52.6 (C4), 40.3 (C18), 30.1 (C7), 29.1 (C14), 25.6 (C6), 22.5 (C15); ESI-MS, $[M+H]^+$: 259.13 (calculated), 259.15 (observed); HRMS (ESI+) $(M+H)^+$: 259.1288 (calculated for $C_{11}H_{19}N_2O_5^+$), 259.1291 (observed).

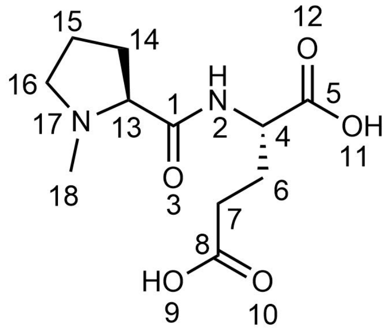

NH-PEPAK-NH_2 Pentapeptide

This peptide was prepared by solid phase synthesis as described above.

^{1}H NMR (600 MHz, D_2O) δ_H = 4.77 – 4.75 (m, 1H, 2), 4.48 – 4.43 (m, 2H, 11, 33), 4.36 – 4.27 (m, 2H, 18, 23), 3.90 – 3.83 (m, 1H, 12''), 3.77 – 3.70 (m, 1H, 12'), 3.51 – 3.38 (m, 2H, 35', 35''), 3.03 (q, *J*=6.9, 6.2, 2H, 28), 2.61 – 2.46 (m, 3H, 5, 36'), 2.40 – 2.32 (m, 1H, 14'), 2.24 – 2.16 (m, 1H, 37''), 2.13 – 2.03 (m, 5H, 4, 13, 36''), 2.01 – 1.92 (m, 2H, 14'', 37'), 1.91 – 1.84 (m, 1H, 25''), 1.83 – 1.77 (m, 1H, 25'), 1.76 – 1.69 (m, 2H, 27), 1.56 – 1.50 (m, 1H, 26''), 1.49 – 1.46 (m, 1H, 26'), 1.44 (d, *J*=7.2, 3H, 20); ^{13}C NMR (151 MHz, D_2O) δ_C = 177.3 (C6), 176.4 (C24), 175.0 (C19), 173.7 (C15), 171.2 (C34), 169.6 (C3), 60.4 (C33), 59.5 (C11), 53.2 (C23), 51.3 (C2), 49.6 (C18), 48.0 (C12), 46.5 (C35), 39.2 (C28), 30.4 (C25), 29.8 (C5), 29.7 (C36), 29.4 (C14), 26.2 (C27), 25.5 (C37), 24.7 (C4), 23.7 (C13), 22.0 (C26), 16.4 (C20); ESI-MS, $[M+H]^{2+}$: 270.66 (calculated), 270.75 (observed); HRMS (ESI+) $(M+H)^+$: 540.3140 (calculated for $C_{24}H_{42}N_7O_7^+$), 540.3139 (observed).

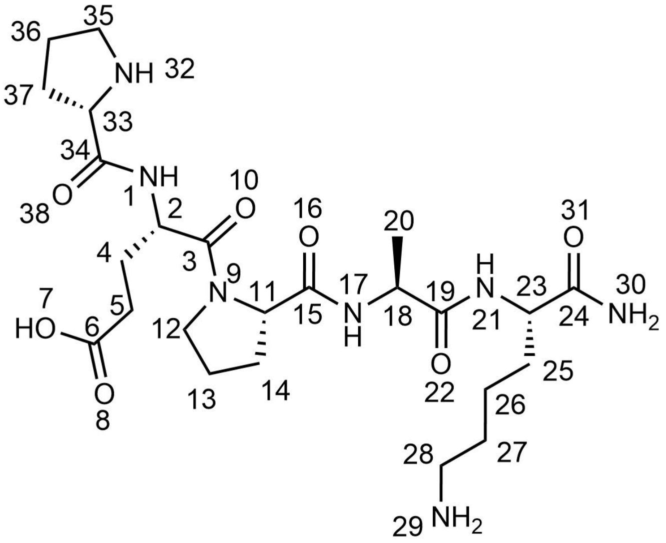

NH-PEPAK-NH_2 Pentapeptide HCHO adduct

300 µL of 22 mM (6.78 umol, 3.6 mg) pentapeptide was reacted with a 100-fold excess of HCHO (678 µmol, 678 µL of a 1 M stock solution) at pH 9 for 12 h. The mixture was then HPLC-purified using an ACE5 C18 column, 100 x 21.2 mm employing an 2%-40% (v/v) MeCN gradient in H_2O, 0.1% formic acid over 30 min. The product was concentrated to give a colourless solid. The yield was approximately 1.4 mg (2.55 µmol, 38%).

^{1}H NMR (600 MHz, D_2O) δ_H = 5.2 (d, *J*=8.95, 1H, 32'), 5.2 – 5.1 (m, 1H, 2), 4.9 (d, *J*=8.99, 1H, 32''), 4.7 (dd, *J*=9.30, 4.54, 1H, 34), 4.4 (dd, *J*=8.39, 6.04, 1H, 11), 4.3 – 4.3 (m, 3H, 18, 23), 3.9 – 3.7 (m, 3H, 12, 36'), 3.4 – 3.3 (m, 1H, 36''), 3.0 (q, *J*=7.73, 3H, 28), 2.5 – 2.4 (m, 2H, 5, 38''), 2.4 – 2.3 (m, 2H, 14', 38'), 2.2 – 2.2 (m, 1H, 4'), 2.1 – 2.0 (m, 4H, 4'', 13, 37), 2.0 – 1.9 (m, 1H, 14''), 1.9 – 1.8 (m, 1H, 25''), 1.8 – 1.8 (m, 1H, 25'), 1.7 – 1.7 (m, 2H, 27), 1.5 – 1.5 (m, 1H, 26), 1.4 (d, *J*=7.22, 4H, 20); ^{13}C NMR (151 MHz, D_2O) δ_C = 177.7 (C6), 176.5 (C19), 175.0 (C24), 170.7 (C35), 168.7 (C3), 168.1 (C15), 65.6 (C34), 65.6 (C32), 60.3 (C11), 57.5 (C36), 53.2 (C23), 52.8 (C2), 49.6 (C18), 48.4 (C12), 39.2 (C28), 30.8 (C5), 30.4 (C25), 29.5 (C14), 27.3 (C38), 26.2 (C27), 24.6 (C13), 24.3 (C37), 24.0 (C4), 22.1 (C26), 16.3 (C20); ESI-MS, $[M+H]^{2+}$: 276.66 (calculated), 276.80 (observed); HRMS (ESI+) $(M+H)^+$: 552.31402 (calculated for $C_{25}H_{42}N_7O_7^+$), 552.31393 (observed).

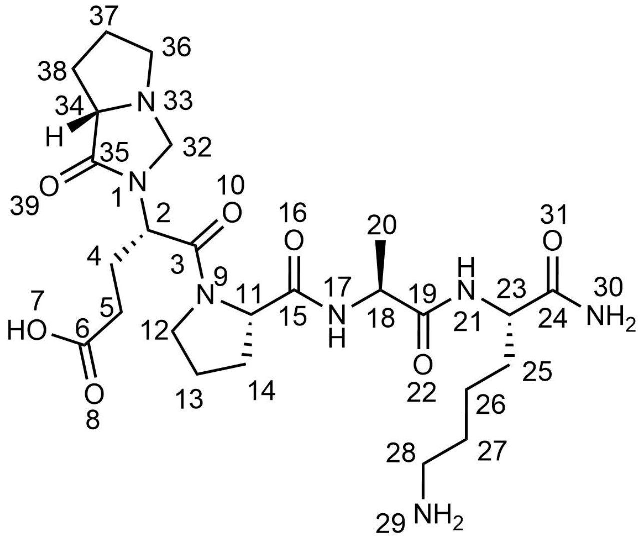

***In situ* NMR analysis of the reaction of the PEPAK pentapeptide with $H^{13}CHO$**

In an alternative approach to investigate whether the reaction with HCHO takes place on the N-terminus of H2B, 1 mM H2B pentapeptide (24 µL of 22 mM stock in H_2O) was mixed with 25 mM $H^{13}CHO$ (126 µL of 100 mM stock in H_2O) and D_2O (510 µL total reaction mixture volume after adjusting to pD 9). The mixture was left for 24 h at ambient temperature, then analysed by NMR, comparing the results with unreacted pentapeptide (**Figure S3a-c**).

NMR Time Course and Stability – Dipeptides

The dipeptide (23.3 µmol) was dissolved in D_2O; the pD was adjusted to 10 (corresponding to pH 9.6) using NaOD and DCl (total volume: 1 mL, 23.3 mM stock). HCHO (30.0 mg, 1.0 mmol) was suspended with D_2O in a sealed microwave vial and heated until a clear colourless solution was obtained; the pD was then adjusted with NaOD and DCl to 10 (total volume: 3 mL, 1/3 M stock). For time course measurements, the dipeptide stock solutions (100 µL, 2.33 µmol) at pD 10 and D_2O (400 µL) were mixed and recorded. HCHO (70 µL, 23.3 µmol, 10 equivalents) at pD 10 was added and progress was monitored for the indicated time. To investigate the stability of the NMR-observed HCHO adducts, a 40-fold excess with respect to starting material of the HCHO scavenger 1,3-cyclohexanedione (1,3-CHD, 93.2 µL from 1 M stock in D_2O) was added.

Additionally, stability studies of the HPLC-purified **2a** and the corresponding pentapeptide PEPAK-HCHO adduct were conducted. To achieve a 40-fold excess of HCHO scavengers (46.8 µmol) with respect to peptide starting material, the appropriate volumes of GSH (186.4 µL from 250 mM stock), cysteine (46.6 µL from 1 M stock) or 1,3-cyclohexanedione (46.6 µL from 1 M stock) were added to the peptide-HCHO adducts (50 µL, 1.17 µmol, 23.3 mM stock in D_2O) in 400 µL of D_2O, thoroughly mixed and recorded. To test for stability of the adduct in different pH conditions, HPLC purified **2a** (45 µL of 23.3 mM stock) was dissolved in 405 µL of 50 mM potassium phosphate buffer (D_2O) at pH 5.9, 7.4 and 8.5 (pD 6.3, 7.8 and 8.9), thoroughly mixed and recorded.

NMR Competition Experiments

For equimolar competition experiments (**Figure S21a+b**, **Figure S22**), D_2O (400 µL) was mixed with component 1 (50 µL, 1.17 µmol, 23.3 mM stock) and component 2 (50 µL, 1.17 µmol, 23.3 mM stock) and NMR spectra recorded. If indicated, equimolar amounts of HCHO were added (6 µL of a 0.2 M stock were used). To test whether **2a** is stable at higher temperatures, the mixture in the NMR tube was heated for 15 min at 75 °C (**Figure S17a, panel 5.**).

Reaction of 2 with other Carbonyl Compounds

To assess whether **2** reacts with carbonyl compounds other than HCHO, **2** (81 µmol) was mixed with up to 100 equivalents of the relevant carbonyl compound at pH 10 and reacted overnight at ambient temperature, 37 °C, and 75 °C. Progress of the reaction was tested by LC/MS; as only acetaldehyde showed reactivity, only the ProGlu-acetaldehyde adduct was HPLC purified. However, upon drying the sample, conversion to the starting material (**2**) was consistently observed (**Figure S19**).

MALDI MS

MALDI MS measurements were carried out using a MALDI Autoflex Speed, Bruker machine. For preparation of α-cyano-4-hydroxycinnamic acid (CHCA) matrix, CHCA was dissolved in MQ water/MeCN (1:1) 0.1% (v/v) CF_3CO_2H (1 mg CHCA per 100 µL mixture) and sonicated. The sample (1 µL) was then spotted with 1 µL of the CHCA solution onto a ground steel target (Bruker) and analysed. HCHO titration experiments were conducted at pH 5.6, 7.4, or 8.2 in 5 µL of potassium phosphate buffer (50 mM), peptide (2.5 µL from 10 µM stock in MQ water), 2.5 µM/25 µM/250 µM/2.5 mM/25 mM HCHO or urea compound (from equimolar to 10^4-fold excess; 2.5 µL from 10 µM/100 µM/1 mM/10 mM stock in MQ water). Reactions were performed with 10 µL final reaction volumes in a 384 well plate (Microplate, 384 well, PP, V-bottom, 781280, Greiner) at ambient temperature or, if indicated, at 37 °C. Plates were sealed before analysis (StarSeal Sealing Tape Polyolefin Film, E2796-9793, StarLab). Samples were taken after 1 min (directly after adding HCHO), 1 h, 2 h, 8 h and 24 h. For the reductive methylation reactions, 2.5 µL of 20 mM potassium phosphate buffer, pH 7.4, 2.5 µL of peptide (10 µM stock in MQ water), 2.5 µL of HCHO (100 µM/200 µM/1 mM stock in MQ water), and 2.5 µL of $NaCNBH_3$ (200 µM/400 µM/2 mM stock in buffer) were used.

MALDI Top-Down MS/MS [7]

For MALDI top-down MS/MS analyses (**Figure 1e+f, Figure S23c+d**), H2B peptide (2.5 µM final concentration) was reacted with a 10^3-fold excess HCHO for 12 h in MQ water (10 µL reaction volume) at ambient temperature, then spotted on a ground steel plate with CHCA. Control conditions without HCHO were: H2B 15mer (100 µM) in MQ water (10 µL reaction volume), then spotted on a ground steel plate with CHCA. Note MQ water was chosen because peptides fragmented better than with the previously used potassium phosphate buffer.

KDM4E Catalysed *in situ* Formation of HCHO – Reaction with H2B 15mer

KDM4E protein sequence:

MHHHHHHSSGVDLGTENLYFQSMKSVHSSPQNTSHTIMTFYPTMEEFADFNTYVAYMESQGAHQAGLAKVIPP
KEWKARQMYDDIEDILIATPLQQVTSGQGGVFTQYHKKKKAMRVGQYRRLANSKKYQTPPHQNFADLEQRYWK
SHPGNPPIYGADISGSLFEESTKQWNLGHLGTILDLLEQECGVVIEGVNTPYLYFGMWKTTFAWHTEDMDLYSINY
LHFGEPKTWYVVPPEHGQHLERLARELFPDISRGCEAFLRHKVALISPTVLKENGIPFNCMTQEAGEFMVTFPYGYH
AGFNHGFNCAEAINFATPRWIDYGKMASQCSCGESTVTFSMDPFVRIVQPESYELWKHRQDLAIVEHTE

Biosynthesis and Purification of KDM4E [8]

The N-terminal-His6 tagged KDM4E (M1-R336) encoding DNA was transformed into BL21(DE3) competent cells. Colonies were used to inoculate 50 mL of LB media containing 50 µg/mL kanamycin and 34 µg/mL chloramphenicol, which was placed in a 37 °C shaker overnight. The next day, 6x 10 mL of the starter culture was used to inoculate 6x 1 litre of TB media containing 50 µg/mL kanamycin in 2 litre baffled shaker flasks. When the OD_{600} was ~0.8, the temperature was reduced to 18 °C and when the OD_{600} was ~0.9, 0.5 mM isopropyl β-D-1-thiogalactopyranoside was added and incubation was continued overnight. The cell suspensions were centrifuged (2600 g, 10 min). The resultant pellets were resuspended in lysis buffer (50 mM HEPES pH 7.4, 500 mM NaCl, 20 mM imidazole, 0.5 mM tris(2-carboxyethyl)phosphine [TCEP], and 5% glycerol (v/v) in the presence of a protease inhibitor mix (Complete, EDTA-free Protease Inhibitor Cocktail, Roche Diagnostics Ltd.)) and lysed by three passages through a high-pressure cell breaker (Avestin Emulsiflex C5) at 4 °C. The lysate was cleared by centrifugation (60 min, 36,000 g, 4 °C) and loaded onto a Ni NTA gravity column.

After rinsing with lysis buffer, the His-tagged protein was eluted in lysis buffer containing 300 mM imidazole. The eluted protein was dialysed to remove imidazole, the N-terminal tag was cleaved using the TEV protease. The cleaved tag and the TEV protease were removed by another Ni NTA gravity column. Finally, the KDM4E protein was concentrated and subjected to gel filtration using an AKTA Xpress system combined with an S200 16/600 gel filtration column and GF buffer (50 mM HEPES pH 7.4, 150 mM NaCl, 5% glycerol and 0.5 mM TCEP). The purity was confirmed by SDS-PAGE and by mass spectrometry.

a) MALDI MS

Table S1: ***In situ*** **formation of HCHO.**

Component	Stock concentration	Volume [µL]	Final concentration
KDM4E	88 µM, 150 mM NaCl, 50 mM HEPES	2	8.8 µM
Sodium ascorbate	10 mM	2	1 mM
Ammonium ferrous sulphate	1 mM in 400 µM HCl	2	100 µM
H2B 1-15	8 mM	3	1200 µM
HEPES	100 mM, pH 7.4	8	40 mM
2OG or NOG control	8 mM	2	800 µM
$H3K9Me_3$ 1-21	10 mM	1	500 µM

The reaction shown in **Table S1** was conducted at ambient temperature and was monitored at 1 min (directly after starting the reaction), 1 h, 2h, 8 h and 24 h (only the 24 h time point is shown in **Figure 2a-d**) using CHCA matrix and MALDI MS as described above. Along with analysis by MALDI MS, the sample after 24 h reaction time was analysed by Orbitrap Elite LC-MS/MS (**Figure S5**).

b) LTQ Orbitrap Elite LC-MS/MS for Peptides

Peptide analyses were performed using a Thermo Scientific Dionex Ultimate 3000 chromatography system coupled directly to an Orbitrap Elite mass spectrometer (Thermo Scientific, San Jose, CA) with a HESI II electrospray ionisation source. A 5 µL partial loop injection was used for all analyses and the chromatographic separation was performed using a Chromolith FastGradient RP-18, 2.0 × 50 mm, 1.6 µm particle size column. The LC flow rate was 0.300 mL/min. The peptides were separated with a linear gradient of 5-95% (v/v) Buffer B (MeCN + 0.1% formic acid) /Buffer A (MilliQ water + 0.1% formic acid) during 10 minutes. Analysis was performed in positive ion mode using a scan range from 410-2000 and resolution set to 15,000. (AGC target 1e6, maximum injection time 250 ms) and subsequent HCD MS/MS spectra (AGC target 5e4, maximum injection time 100 ms) of the parent mass *m/z* 491.920 and 503.92 peaks were acquired in the FTMS. HCD fragmentation was performed at 35% of normalized collision energy. All spectra were manually checked and validated or disqualified. The analysis was performed with Xcalibur software.

Tissue Culture

Cells were from ATCC (HEK293T reference no. CRL-3216). The authentication from ATCC was relied upon. Cell lines were regularly tested to be mycoplasma negative (MycoAlert Mycoplasma Detection Kit ref. no LT07-218). Cell lines were cultured in Dulbecco's Modified Eagle Medium (DMEM, D6546, Sigma) supplemented with 10% (v/v) FBS (Gibco) and 1% (v/v, 2mM final concentration) GlutaMAX (Gibco) in a 37 °C incubator at 5% CO_2. For harvesting the cells, the medium was aspirated and cells were washed twice with phosphate buffered saline (PBS, Sigma, D8537). Upon aspirating PBS, radioimmunoprecipitation assay buffer (1x RIPA [Sigma, R0278], supplemented with protease inhibitor [cOmplete™, Mini, EDTA-free Protease Inhibitor Cocktail, Roche]), was used to scrape the cells. The cell suspension in the reaction tube was either frozen at -20 °C or incubated for 45 min on ice and vortexed several times during that time interval. After centrifuging (16,000 g, 15 min, 4 °C), the supernatant was used for analyses.

SDS-PAGE Analyses

Polyacrylamide gel electrophoresis was performed using NuPAGE 4-12% Bis-Tris Protein Gels (Life Tech) in 1x MOPS running buffer (20x NuPAGE MOPS SDS Running Buffer, NP0001). SDS-PAGE was run at 170 V for 40 min (Mini Gel Tank, Life Technologies). PageRuler Prestained Protein Ladder (Thermo Scientific, reference 26617) was used to compare protein sizes.

Western Blot Analyses

Proteins resolved by SDS-PAGE were transferred onto a nitrocellulose membrane (Amersham Protran Premium 0.2 NC 300 mm, GE Healthcare) using a current of 350 mA for 60-90 min or 85 mA overnight (Mini Protean Tetra Cell, Bio-Rad) in transfer buffer containing 10% (v/v) methanol (20x NuPAGE Transfer Buffer, Invitrogen). Membranes were blocked using 5% milk (w/v) in PBS 1‰ tween buffer (PBST; Tween-20, Sigma-Aldrich) at ambient temperature for at least 30 min. The membranes were incubated with the appropriate primary antibody (1:1000) in 5% milk (w/v) PBST at 4 °C overnight on a shaker. After three 10-min washes with PBST at ambient temperature, the blots were incubated with the appropriate secondary antibody (in 5% milk PBS-Tween in a 1:5,000 dilution) for 1 h at ambient temperature followed by three 10-min washes with PBST at ambient temperature and detection using GE Healthcare Amersham ECL Prime Western Blotting Detection Reagent (RPN2236) and Bio-Rad Universal Hood iii. Primary antibodies used: GAPDH Loading Control Monoclonal Antibody (GA1R) (Invitrogen, MA5-15738), H2B (ab52484), H2A (EPR17470), H3 (ab10799), leukotriene A4 hydrolase

(ab133512). Secondary antibodies used were anti-mouse IgG, HRP-linked Antibody (Cell Signaling, reference 7076), anti-Rabbit IgG (H+L) HRP Conjugate (Promega reference W4011).

RNA-Seq Analyses

HEK293T cells ($0.9x10^6$) were seeded into 6 cm dishes as biological triplicates. The next day, cells were treated with 150 µM HCHO (or MQ water control), followed by another cumulative treatment of 225 µM HCHO (or MQ water control) the day after that. Cells were harvested 4 h after the second treatment. For harvesting, cells were washed twice with ice-cold PBS (Sigma, D8537); RNA was isolated using the PureLink™ RNA Mini Kit (Invitrogen, 12183020) following the manufacturer's recommendations on "purification from plant and animal cells", "monolayer cells", "syringe homogenization", and "on-column DNase treatment" (PureLink DNase Set, Invitrogen, 12185010). Samples were sent for RNA-Seq PolyA to the Oxford Genomics Centre.

For transcript-level expression analysis, reads were mapped for each sample to the reference genome (ftp.ensembl.org/pub/release-77/gtf/homo_sapiens/Homo_sapiens.GRCh38.77.gtf.gz) using HISAT[9]; the resulting SAM files were sorted and converted to BAM files using SAMtools.[10] The resulting BAM files were merged with Stringtie[11] and transcript abundances were estimated and table counts created for Ballgown[12] using the differential expression analysis protocol described by Pertea et al.[13]

Biosynthesis of Recombinant 4-Oxalocrotonate Tautomerase (4-OT)

An open reading frame encoding for 4-OT from *Pseudomonas putida* was purchased from GenScript as codon-optimized sequence for *E. coli*, and provided in a plasmid cloning vector. The sequence of the purchased gene is given below and is flanked by a NdeI site at the 5' end and by a BamHI site at the 3' end.

5'CATATGCCGATTGCGCAGATCCACATCCTGGAAGGTCGTAGCGATGAACAGAAAGAAACCCTGATTCGTGA
AGTTAGCGAGGCGATTAGCCGCAGCCTGGATGCGCCGCTGACCAGCGTGCGTGTTATCATTACCGAGATGGC
GAAGGGTCATTTT GGTATCGGTGGTGAACTGGCGAGCAAAGTGCGTCGTTAAGGATCC-3'

DNA encoding for 4-OT was subcloned into a pET-22b(+) vector using NdeI and BamHI restriction sites and transformed into XL10 gold cells (NEB, C3040H). Clones were sequence verified (Eurofins); the expression plasmid was transformed into the host strain (BL21(DE3) competent *E. coli* (NEB, C2527H)). Several colonies were combined to generate a glycerol stock. 2TY (+50 µg/mL ampicillin) was inoculated with cells from a glycerol stock overnight. The next day, 4x 600 mL (+50 µg/mL ampicillin) were inoculated with 1:100 (v/v) overnight culture and left at 37 °C, 180 rpm overnight (no IPTG added). The cell harvesting and purification conditions are modified from those of Lukesch et al.[14] The next day (24 h after reinoculation), the cells were centrifuged (4250 g, 30 min, 4 °C) and washed with 10 mM NaH_2PO_4, pH 8 (buffer A). The pellet (19.74 g from 2.4 L culture) was then frozen directly at -80 °C for storage. 5 g of the thawed pellet was resuspended in 25 mL of buffer A and sonicated (10 min, 9.9 s on, 9.9 s off, 38% amplitude). After centrifugation (48384 g, 30 min, 4 °C), the supernatant was made up to 1.6 M in $(NH_4)_2SO_4$ by adding the solid salt and gradually dissolving it overnight at 4 °C on a tube roller. After centrifugation (48384 g, 30 min, 4 °C) and filtration (0.45 µm), the cleared lysate was desalted using a HiPrep 26/10 column (GE Healthcare, Vienna, Austria) on an Äkta pure FPLC system (GE Healthcare), which was conditioned with buffer A. The lysates were then loaded as 2x 15 mL with a flow of 2 mL/min. Buffer A was used to elute the proteins (flowrate of 2 mL/min, monitoring λ_{max} 280 nm), collecting fractions containing purified 4-OT (**Figure S37a**). Next, the protein eluate (without concentration) was loaded onto a 11 mL DEAE Fast Flow ion exchange column (GE Healthcare; self-packed in a XK 16 column from GE Healthcare) previously equilibrated with Buffer A. After loading at 2 mL/min, the column was left for 15 min to allow protein binding to

the column. Non-binding proteins were removed by washing the column with buffer A, as indicated by a stable A280 trace. A gradient elution for 7 column volumes from buffer A to buffer B (10 mM NaH_2PO_4, 90 mM Na_2SO_4, pH 8) with a flow rate of 2 mL/min was employed. Fractions containing purified 4-OT were collected in 5 mL tubes using a fraction collector (at A205 because 4-OT does not contain aromatic amino acids), which was confirmed by tris-tricine-SDS-PAGE (**Figure S37b+c, Table S2**); the mass determined by solid phase extraction coupled to mass spectrometry (SPE-MS) was 6811.51 Da (calculated: 6810.84 Da). UV-absorption at 205 nm (Nanodrop) and BCA assay (Thermo Fisher, reference 23225) were used to determine the yield of 4-OT-containing fractions, which were subsequently aliquoted, rapidly frozen and stored at -80 °C. The yield was 5 mL of 5.4 mg/mL (as determined by a BCA assay, Thermo Fisher, ref. 23225) from 5 g of starting pellet.

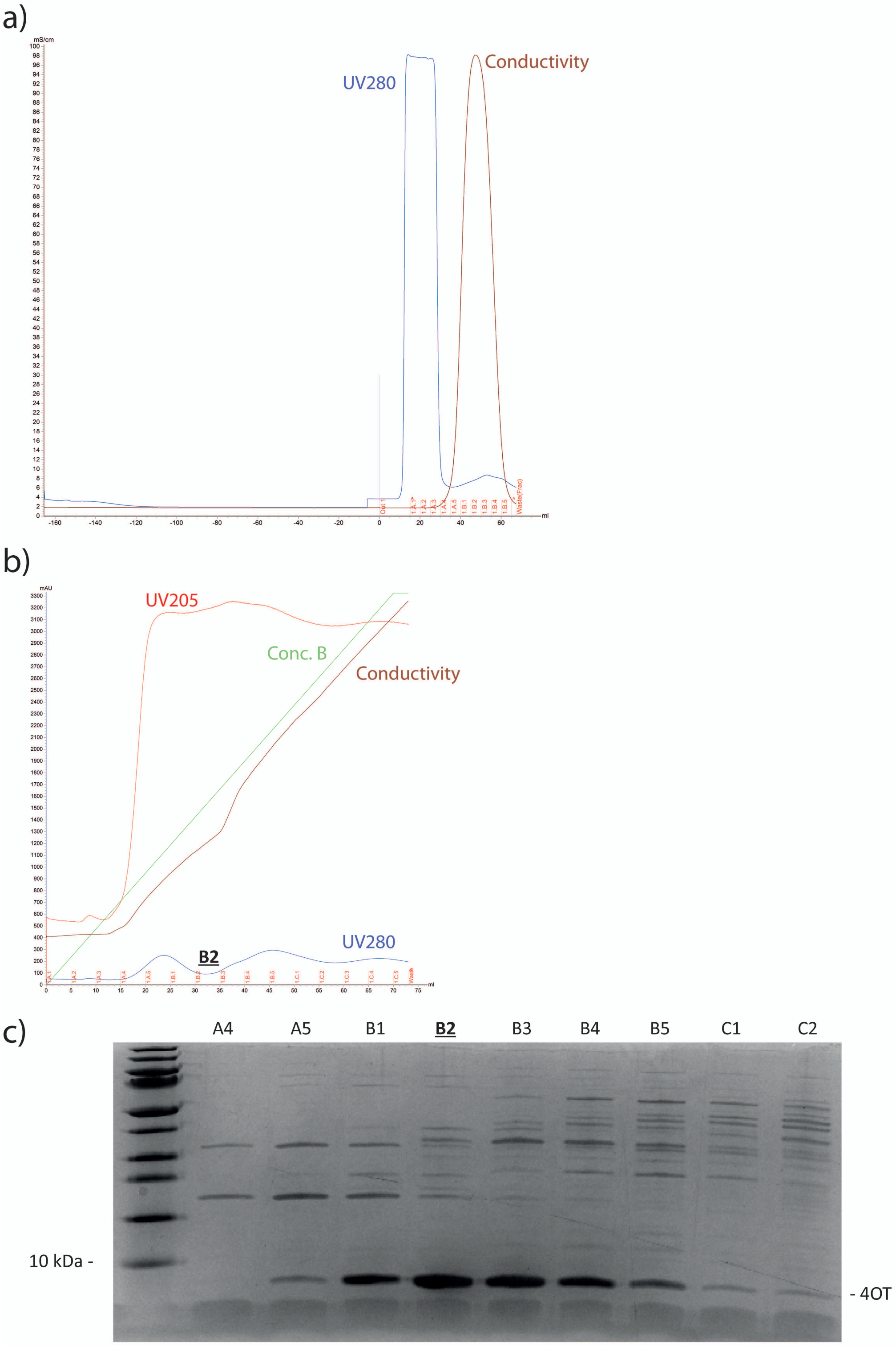

Figure S37 | Chromatograms and fractions obtained after desalting and ion exchange chromatography. a) Chromatogram for desalting column. **b)** Chromatogram for anion exchange column. **c)** Tris-tricine gel (see **Table S2**) showing fractions containing 4-OT. The bold/underlined fraction B2 was used for further studies.

Table S2: Tris-tricine SDS-PAGE gel and buffers.

Component	Volume
**Separating Gel (10%)**	
H_2O	1.75 mL
3 M tris-HCl pH 8.45/10 mM SDS	2.5 mL
30% Bis-acrylamide 29:1	2.5 mL
Glycerol	0.75 mL
30% Ammonium persulfate (APS)	7 µL
Tetramethylethylenediamine (TEMED)	7 µL
**Stacking Gel (4%)**	
H_2O	1.95 mL
3 M tris-HCl pH 8.45/10 mM SDS	0.775 mL
30% Bis-acrylamide 29:1	0.4 mL
30% APS	7 µL
TEMED	7 µL
10x Anode Buffer: 2 M tris, pH 8.8	
10x Catode Buffer: 1 M tris/1 M tricine, pH 8.3, 1% (w/v) SDS	

4-OT NMR Assays

The 4-OT-catalysed reaction of acetaldehyde to *trans*-β-nitrostyrene was followed by ^{1}H NMR (700 MHz) (**Table S3**), performed in two independent duplicates.

Table S3: 4-OT NMR assay in 10% DMSO-D_6.

Component	Stock	Volume [µL]	Final Conc.
Potassium phosphate	20 mM, pH 7.8	275	
Acetaldehyde	75 mM in buffer	200	20 mM
Trans-β-nitrostyrene	3 mM in DMSO-D_6	75	0.3 mM
4-OT	198.25 µM	200	52.87 µM
		750 µL total vol.	10% DMSO-D_6

For the NMR assay shown in **Table S3**, 50 µL of 4-OT (793 µM stock) was preincubated at 37 °C for the indicated times with 150 µL of potassium phosphate buffer, 20 mM, pH 7.8 (control condition) or 150 µL of 52.87 mM HCHO in potassium phosphate buffer, resulting in 200 µL of 198.25 µM 4-OT (with a 200-fold molar excess of HCHO relative to 4-OT). All components apart from 4-OT (buffer, acetaldehyde, *trans*-β-nitrostyrene) were mixed thoroughly in an NMR tube and a ^{1}H (700 MHz) spectrum was recorded. Upon addition of 4-OT, progress of the reaction was followed (298 K).

4-OT Absorbance Assays

The reaction of acetaldehyde with *trans*-β-nitrostyrene was monitored by absorbance measurements at 320 nm (**Table S4**, **Table S5**, **Table S6**). Each condition was performed in technical triplicate and in two independent duplicates. The kinetic parameters for both substrates *trans*-β-nitrostyrene (**Table S4**) and acetaldehyde (**Table S5**) of 4-OT were determined by monitoring the 4-OT-catalysed reaction of acetaldehyde with *trans*-β-nitrostyrene over time; the conditions for acetaldehyde were derived from those reported by Zandvoort et al. [15] (**Figure S27a-d**). The different substrate stocks were prepared as 4-fold stock solutions. Experiments were performed in independent duplicates using Greiner UV-star plates (ref. 655801) on a Clariostar^{Plus} plate reader (BMG Labtech) at ambient temperature. The slopes of the initial reaction rate were fitted with the Michaelis-Menten equation (Et = 10 µM) using non-linear regression (GraphPad Prism 5) to obtain the K_M and k_{cat} values.

Table S4: Kinetic parameters for *trans*-β-nitrostyrene.

Component	Stock	Volume [µL]	Final Conc.
Trans-β-nitrostyrene	4-fold in 40% DMSO/60% buffer	50	15.625-500 µM
Potassium phosphate	20 mM, pH 7.4	50	
Acetaldehyde	200 mM in buffer	50	50 mM
4-OT	40 µM in buffer	50	10 µM
		200 µL total vol.	10% DMSO

Table S5: Kinetic parameters for acetaldehyde.

Component	Stock	Volume [µL]	Final Conc.
Trans-β-nitrostyrene	1.5 mM in 40% DMSO/60% buffer	50	375 µM
Potassium phosphate	20 mM, pH 7.4	50	
Acetaldehyde	4-fold in buffer	50	5-375 mM
4-OT	40 µM in buffer	50	10 µM
		200 µL total vol.	10% DMSO

Table S6: 4-OT absorbance assay in 10% (v/v) DMSO.

Component	Stock	Volume [µL]	Final Conc.
Potassium phosphate	20 mM, pH 7.8 or 7.4	0, 50 or 100	
4-OT	40 µM in buffer	50	10 µM
Acetaldehyde	75 mM or indicated conc. of CH_3CHO in buffer	50	18.75 mM
Trans-β-nitrostyrene	0.8 mM in 40% DMSO/60% buffer	50	0.2 mM
		200 µL total vol.	10% DMSO

For the absorbance assay results shown in **Table S6**, 50 µL of 4-OT (40 µM stock, diluted in potassium phosphate buffer, 20 mM, pH 7.8 or pH 7.4) was preincubated at 37 °C for the indicated times with 50 µL of potassium phosphate buffer (control condition) or 50 µL of 8 mM HCHO stock (prepared in buffer, resulting in a 200-fold molar excess of HCHO relative to 4-OT) in a 96-well plate (Corning Clear Flat Bottom, reference 3370). At the end of the indicated incubation times, 0.8 µL of the pre-incubated enzyme was spotted with 0.8 µL of CHCA matrix on a ground steel target (Bruker) and analysed by MALDI MS (**Figure S29a**). This was followed by the addition of acetaldehyde, then *trans*-β-nitrostyrene (in that sequence) into the 96-well plate, and after thorough mixing by pipetting up and down five times. The absorbance spectra were recorded on a Clariostar^{Plus} or Pherastar (BMG Labtech) plate reader at ambient temperature (**Figure S28b+c**). For experiments involving higher acetaldehyde concentrations (**Figure S28d**), the acetaldehyde stocks were adjusted accordingly, while keeping the added volume of acetaldehyde (50 µL) solution constant. For control experiments without 4-OT, 100 µL of potassium phosphate was added to 50 µL of acetaldehyde and 50 µL *trans*-β-nitrostyrene.

Circular Dichroism (CD) Measurements

CD analyses used a Chirascan CD spectrometer (Applied Photophysics) equipped with a Peltier temperature-controlled cell holder. The spectra were obtained ranging from 260-185 nm in 0.5 nm intervals in triplicate at 23 °C. Upon subtracting background signal for buffer only, the spectra were smoothed and averaged using the Savitzky–Golay filter (window size 4). Upon normalisation of data to the protein concentration, the mean residue ellipticity (MRE) was calculated with the following formula: MRE = θ/(10 x l x N x C) deg cm^2 $dmol^{-1}$; θ: degree of ellipticity, l: path length (0.1 cm), N: number of amino acids, C: Concentration (mol/L). For CD measurements, 100 µL of 4-OT (in 20 mM potassium phosphate buffer, pH 7.8, 40 µM) were incubated with 100 µL of 8 mM HCHO (prepared in buffer) for 48 h at 37 °C (control: 100 µL buffer), resulting in 20 µM of 4-OT (0.136 mg/mL).

Whole-cell BL21(DE3) Assays

The whole-cell assays were modified from those reported by Narancic et al. [16] In brief, 2TY (+50 µg/mL ampicillin) was inoculated with BL21(DE3) cells (NEB, C2527H) containing the pET22b_4-OT or a pET22b_EV (empty vector) construct from a glycerol stock overnight. The next morning, an overnight culture was inoculated with 1:100 (v/v) starter culture and left at 37 °C, 180 rpm overnight (no IPTG added). The next morning, samples for SDS-PAGE analyses were withdrawn, supplemented with 2x Laemmli buffer and boiled for 5 min at 95 °C (**Figure S32a**). Cells were harvested (5000 g, 10 min, ambient temperature) and the cell pellets were resuspended in 20 mM potassium phosphate buffer,

pH 7.4 to 5 g CDW/l (CDW= cell dry weight). The cell suspension (60 mL) was added to 250 mL Schott Duran glass bottles. HCHO was added to the indicated final concentration from a 100 mM stock in MQ water, and corresponding volume of MQ water only was added as control. After adding acetaldehyde to 20 mM final concentration (2 M stock in MQ water) and *trans*-β-nitrostyrene to 2 mM final concentration (200 mM stock in EtOH), 800 µL were withdrawn from each condition (time point 0). The assay was conducted at 28 °C with shaking at 150 rpm, each condition in duplicate. Withdrawn samples were centrifuged (13000 g, 4 °C, 5 min) and 50 µL of supernatant was then mixed with 150 µL of potassium phosphate buffer (20 mM, pH 7.4). The absorbance at 320 nm was measured immediately using Pherastar (BMG Labtech) in a 96-well plate (Flat bottom, Costar, reference 3370) in 5 technical replicates.

At the end of the whole-cell assay, duplicate pET22b_4-OT reaction mixtures were pooled (100 mL, 2 mM *trans*-β-nitrostyrene at the start of the assay, 200 µmol) and centrifuged (5000 g, 10 min, ambient temperature). The supernatant was then extracted and purified following the protocol of Narancic et al.[16] In brief, the supernatant was extracted with 4x 40 mL ethyl acetate. The combined organic extracts were dried (Na_2SO_4), filtered and concentrated *in vacuo*. The crude product was purified using 40% (v/v) EtOAc in cyclohexane over 24 column volumes (Biotage Sfär Silica D, 5 g) to yield a yellowish oil. Yield: approximately 9.2 mg, 24%, 47.7 µmol.

4-Nitro-3-phenylbutanal

^{1}H NMR (400 MHz, $CDCl_3$) δ = 9.71 (t, J=1.2 Hz, 1H, 11), 7.36 – 7.22 (m, 5H, 4, 5, 6, 7, 8), 4.65 (qd, J=12.5, 7.4 Hz, 2H, 9), 4.08 (p, J=7.3, 1H, 2), 3.03 – 2.88 (m, 2H, 1); ^{13}C NMR (101 MHz, $CDCl_3$) δ = 198.9 (C10), 138.3 (C3), 129.4 (C5, C7), 128.3 (C6), 127.5 (C4, C8), 79.6 (C1), 46.6 (C9), 38.1 (C2). ESI-MS, $[M+H]^-$: 192.07 (calculated), 192.2 (observed).

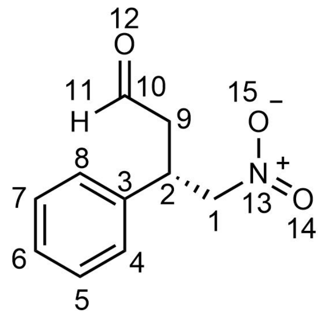

Bacterial Growth Analyses

Multiple colonies from a freshly streaked ampicillin plate with BL21(DE3) cells (NEB, C2527H) containing the pET22b-4-OT construct were resuspended in sterile PBS (Sigma, D8537) to an OD_{600} of 0.1, which was then diluted 1/10 in PBS. The resulting cell suspension (20 µL) were then added to 180 µL of 2TY (without antibiotic) containing the respective amounts of HCHO or an equivalent volume of sterile MQ water in a sterile 96-well plate (Flat bottom, Costar, reference 3595), resulting in the final HCHO concentrations indicated. The assay was conducted using a Pherastar machine (BMG Labtech) at 37 °C (absorbance 600 nm), with shaking before each cycle (double orbital movement) at 300 rpm for 30 s (**Figure S32b**). The plate was then covered with a Star Seal advanced Polyolefin film (E2796-9795) to reduce evaporation.

Immunoprecipitation

Immunoprecipitations were performed using Dynabeads™ Protein G for Immunoprecipitation (Invitrogen), adapted from the manufacturer's recommendations. In brief, the Dynabeads were vortexed in the vial for 30 s to resuspend the magnetic beads. The bead suspension (50 µL) was transferred to a 1.5 mL Eppendorf tube, which was placed on a magnet and the supernatant removed. The tube was removed from the magnet and the antibody for the target protein (7.5 µg), diluted in 200 µL of PBS (0.02% Tween 20, v/v) was added to the magnetic beads and incubated with rotation (10 min, ambient temperature). The tube was placed on the magnet and the supernatant removed. The tube was removed from the magnet and the beads were washed with 200 µL of PBS (0.02% Tween 20) by gentle pipetting. The tube was placed on the magnet and the supernatant removed. The RIPA lysed sample (2 mg) containing the antigen diluted with 200 µL of PBS (0.02% Tween 20) was added to the bead-antibody complex which was resuspended by gentle pipetting. This mixture was incubated with rotation for 2 h at ambient temperature, which enabled the antigen to bind to the bead-antibody complex. The tube was then placed on the magnet and the supernatant was transferred to a clean tube for analysing binding efficiency of the antigen. The bead-antibody-antigen complex was washed 3 times using 200 µL of PBS (without Tween 20); the supernatant was removed with aid of the magnet after each wash. The bead-antibody-antigen complex was resuspended using 100 µL of PBS (without Tween 20) and transferred to a new tube to prevent co-elution of proteins attached to the tube. The tube was placed on the magnet to remove the supernatant. 50 mM glycine pH 2.8 (20 µL) and 10 µL of 2x NuPAGE™ LDS Sample buffer containing dithiothreitol (DTT, dissolved in 100 µL LDS Sample buffer 4x buffer to 0.04 M, which was then diluted 1:1 with MQ water to result in 2x sample buffer) were used to resuspend the bead-antibody-antigen complex. The antibody-antigen complex was released from the magnetic beads by heating the sample for 10 min at 70 °C. The tube was placed on a magnet and the supernatant was used for downstream analyses, namely western blot and tryptic digestions.

In-gel Trypsin Digestion

Proteins resolved by SDS-PAGE were excised with a scalpel, cut into 1-2 mm pieces and placed in a 1.5 mL tube. Wash solution (200 µL; 50% MeOH, 5% acetic acid in MQ water, v/v) was added to the gel pieces and incubated overnight at ambient temperature while shaking. The wash solution was removed, followed by addition of 200 µL of wash solution and rinsing for 2-3 h at ambient temperature. The wash solution was removed upon centrifugation. MeCN (200 µL) was added to dehydrate the gel pieces for 5 min at ambient temperature, followed by centrifugation and removal of MeCN. The dehydration step was repeated using again 200 µL of MeCN (5 min, ambient temperature, removal of MeCN). Dithiothreitol (DTT; 30 µL of 10 mM DTT in 100 mM NH_4HCO_3) were used to reduce the sample (30 min, ambient temperature), followed by removal of the solution. Iodoacetamide (IAA; 30 µL of 50 mM IAA in MQ water) were used to alkylate the sample (30 min, ambient temperature), followed by removal of the solution. MeCN (200 µL, 5 min, ambient temperature) was used to dehydrate the gel pieces, resulting in a white colour of the gel pieces. Upon removal of MeCN, the sample was rehydrated (200 µL of 100 mM NH_4HCO_3, 10 min, ambient temperature), followed by removal of the solution. MeCN (200 µL, 5 min, ambient temperature) was used to dehydrate the gel pieces, followed by removal of the solution. The lyophilised trypsin pellet (20 µg, Pierce Trypsin Protease, MS Grade) was reconstituted using 1 mL of ice-cold 50 mM NH_4HCO_3, resulting in a 20 ng/µL stock solution (10x stock). Upon dilution of the 10x trypsin stock using 50 mM NH_4HCO_3 to 1x, 30 µL of the 1x trypsin stock were added to the sample and incubated (10 min on ice, occasional gentle mixing). Upon centrifugation, excess trypsin solution was removed. NH_4HCO_3 (5 µL of a 50 mM solution) was added to the sample to carry out the trypsin digestion (37 °C overnight).

NH_4HCO_3 (50 µL of a 50 mM solution) was added to the sample and incubated (10 min, ambient temperature, occasional vortex mixing). The supernatant was collected in a fresh 1.5 mL collection tube. Extraction buffer 1 (50 µL of 50% MeCN, 5% formic acid in MQ water, v/v) was added to the tube containing the gel pieces and incubated (10 min, ambient temperature, occasional vortex mixing), followed by addition of the supernatant to the collection tube. Extraction buffer 2 (50 µL of 85% MeCN, 5% formic acid in MQ water, v/v) was added to the tube containing the gel pieces and incubated (10 min, ambient temperature, occasional vortex mixing), followed by addition of the supernatant to the collection tube. The sample in the collection tube was dried (Eppendorf Concentrator Plus, >2 h, 30 °C, 1400 rpm with rotor F-45-48-11, V-AQ mode) and resuspended in 20 µL of buffer A (98% MQ water, 2% MeCN, 0.1% formic acid, v/v), followed by analysis using LUMOS LC-MS/MS.

In-solution Trypsin Digestion

The procedure for trypsin digestion was modified from that of Liu et al. [17] If recombinant H2B (human histone H2B, M2505S, NEB) was used, 15 µg of H2B was treated with a 100-fold excess of HCHO in MQ water for 24 h at ambient temperature. If the protein was immunoprecipitated from HEK293T cells, the whole target antigen elution was used after confirming the presence of the antigen by western blot analysis. First, the sample was reduced with dithiothreitol (DTT; 2 µL of 85 mM DTT in 50 mM ammonium bicarbonate) for 40 min at 56 °C. The samples were then alkylated with iodoacetamide (IAA; 7 µL of 55 mM iodoacetamide) for 30 min in the dark at ambient temperature. To remove excess IAA, 3 µL of 85 mM DTT in 50 mM ammonium bicarbonate were added and the mixture was incubated for 10 min in the dark at ambient temperature. A subsequent acetone precipitation step was only performed if proteins had been immunoprecipitated; 6 volumes of ice-cold acetone were used to precipitate the sample, whilst vortexing several times, followed by storage overnight at -20 °C. The sample was then centrifuged (15000 g, 10 min, 4 °C), the supernatant was removed, the pellet was dried (5 min) and resolubilised with 27 µL of 50 mM ammonium bicarbonate. Digestion was performed using Pierce Trypsin Protease, MS Grade. The lyophilised trypsin pellet (20 µg) was reconstituted using 20 µL of 50 mM acetic acid and 60 µL of 50 mM ammonium bicarbonate, resulting in a 0.25 µg/µL stock solution. The trypsin stock solution (3 µL) was added to the sample and incubated overnight at 37 °C. To promote digestion in folded parts of the protein, another digestion step was performed in 80% (v/v) aqueous MeCN; thus, 1 µL of trypsin stock solution and 124 µL of MecN were added to the sample, followed by incubation at 37 °C for 3 hours. Digestion was stopped by adding 5% (v/v) aqueous formic acid and the sample dried using vacuum centrifugation (Eppendorf Concentrator Plus, >2 h, 30 °C, 1400 rpm with rotor F-45-48-11, V-AQ mode). For immunoprecipitated samples, the pellet was resolubilised in 20 µL 0.1% formic acid, 98% MQ water, 2% (v/v) aqueous MeCN and analysed using an LTQ Orbitrap Elite nanoLC-MS/MS machine. For recombinant H2B, the sample peptides were resolubilised in 20 µL of MQ water containing 0.1% (v/v) aqueous formic acid. Samples were purified using ZipTip (OMIX C18 pipette tips, 10 µL tip, 2-10 µL elution volume). The ZipTip was equilibrated using 10 µL of buffer B (60% MeCN, 40% MQ water, 0.1% (v/v) aqueous formic acid, which was aspirated and dispensed twice; to finish the equilibration, 10 µL of buffer A (98% MQ water, 2% MeCN, 0.1% formic acid, v/v) was used, which was aspirated and dispensed twice. To bind the peptides, 10 µL of sample was aspirated and dispensed within the sample tube 10 times. After dispensing for a final time, 10 µL of buffer A were used to wash the peptides bound to the ZipTip, followed by dispensing into waste; this washing step was repeated 4 times. Peptides were eluted into a new tube using 20 µL of buffer B. Samples were then dried using vacuum centrifugation (Eppendorf Concentrator Plus, >2 h, 30 °C, 1400 rpm with rotor F-45-48-11, V-AQ mode) and resuspended in 20 µL of 0.1% (v/v) aqueous formic acid in MQ water for LTQ Orbitrap Elite LC-MS/MS analyses.

LTQ Orbitrap Elite nanoLC-MS/MS for trypsin digest peptides

Peptides resulting from trypsin digestion were analysed using a NanoAcquity-UPLC system (Waters) connected to an Orbitrap Elite mass spectrometer (Thermo Fischer Scientific) possessing an EASY-Spray nano-electrospray ion source (nESI) (Thermo Fischer Scientific). The peptides were initially bound on a packed guard column (75 µm i.d. x 20 mm, Acclaim Pepmap100 C18, 3µm, 120 Å) using solvent A (0.1% formic acid in water, v/v) at a pressure of 140 bar. The peptides were separated using an EASY-spray Acclaim PepMap® analytical column (75 µm i.d. × 15 mm, RSLC C18, 3 µm, 100 Å) using a 68 min linear gradient ranging from 3 to 97% of solvent B (0.1% formic acid in MeCN, v/v). The flow was set to 300 nL/min and the column was heated to 40 °C. The nESI source was operated at a needle voltage of 1600 V, and the ion transfer tube temperature was set to 275 °C. The separated peptides were electrosprayed directly into the spectrometer operating in a data-dependent mode using a CID based method. Full scan MS spectra (scan range 350-1500 *m/z*, resolution 120000, AGC target 1e6, maximum injection time 250 ms) and subsequent CID MS/MS spectra (AGC target 5e4, maximum injection time 100 ms) of the 10 most intense peaks were acquired in the Ion Trap. CID fragmentation was performed at 35% of normalized collision energy and the signal intensity threshold was kept at 500 counts. The CID method used performs beam-type CID fragmentation of the peptides.

Analyses of mass spectra employed Peaks 8.5. The raw MS files were searched against the respective protein sequence. Trypsin with a maximum number of 3 missed cleavages and one unspecific end was selected as the protease in Peaks 8.5. Carbamidomethylation (cysteine) was set as a fixed modification, oxidation (methionine), deamination (asparagine, glutamine) and +12 Da on N-terminal proline were set as variable modifications. Precursor mass tolerance was set as 15 ppm. Fragment mass tolerances for CID was set to 0.8 Da. All spectra were manually validated. For all peptides present at -10lgP> 20, spectra were manually checked and validated or disqualified.

LUMOS LC-MS/MS

Dried peptides were reconstituted in 5% DMSO and 5% formic acid (v/v) and were analysed by LC-MS/MS using an Ultimate 3000 UHPLC machine (ThermoFisher Scientific) connected to an Orbitrap Fusion Lumos Tribrid machine (ThermoFisher Scientific). In brief, peptides were loaded onto a PepMapC18 column (300 µm x 5mm, 5 µm particle size, Thermo Fischer) and separated on a 50 cm-long EasySpray column (ES803, Thermo Fischer) with a gradient of 2-35% MeCN in 5% DMSO, 0.1% formic acid (v/v) at 250 nL/min flow rate over 60 min. Eluted peptides were then analysed using an Orbitrap Fusion Lumos Tribrid platform (instrument control software v3.3). Data were acquired in data-dependent mode, with the advance peak detection (APD) enabled. Survey scans were acquired in the Orbitrap at 120 k resolution over a *m/z* range of 400 -1500, AGC target of 4e5 and S-lens RF of 30. Precursor ions were isolated in the Quad (1.6 isolation window), fragmented in the HCD cell with 30% CE), and analysed in the Orbitrap at 30K resolution with an AGC target of 5e4 and 54 ms maximum injection time and a 7 s dynamic exclusion list.

Supplementary References

[1] R. J. Hopkinson, P. S. Barlow, C. J. Schofield, T. D. Claridge, *Org. Biomol. Chem.* **2010**, *8*, 4915-4920.

[2] S. V. Lehmann, U. Hoeck, J. Breinholdt, C. E. Olsen, B. Kreilgaard, *Contact Derm.* **2006**, *54*, 50-58.

[3] T. R. Malla, A. Tumber, T. John, L. Brewitz, C. Strain-Damerell, C. D. Owen, P. Lukacik, H. H. Chan, P. Maheswaran, E. Salah, *ChemComm* **2021**, *57*, 1430-1433.

[4] S. K. Bharti, R. Roy, *Trends Anal. Chem.* **2012**, *35*, 5-26.

[5] V. Poláčková, P. Čmelová, R. Górová, R. Šebesta, *Monatsh. Chem.* **2018**, *149*, 729-736.

[6] B. Metz, G. F. Kersten, P. Hoogerhout, H. F. Brugghe, H. A. Timmermans, A. De Jong, H. Meiring, J. ten Hove, W. E. Hennink, D. J. Crommelin, *J. Biol. Chem.* **2004**, *279*, 6235-6243.

[7] R. Soares, C. Franco, E. Pires, M. Ventosa, R. Palhinhas, K. Koci, A. M. de Almeida, A. V. Coelho, *J. Proteom.* **2012**, *75*, 4190-4206.

[8] N. R. Rose, S. S. Ng, J. Mecinovic, B. M. Liénard, S. H. Bello, Z. Sun, M. A. McDonough, U. Oppermann, C. J. Schofield, *J. Med. Chem.* **2008**, *51*, 7053-7056.

[9] D. Kim, B. Langmead, S. L. Salzberg, *Nat. Methods* **2015**, *12*, 357-360.

[10] H. Li, B. Handsaker, A. Wysoker, T. Fennell, J. Ruan, N. Homer, G. Marth, G. Abecasis, R. Durbin, *Bioinformatics* **2009**, *25*, 2078-2079.

[11] M. Pertea, G. M. Pertea, C. M. Antonescu, T.-C. Chang, J. T. Mendell, S. L. Salzberg, *Nat. Biotechnol.* **2015**, *33*, 290-295.

[12] A. C. Frazee, G. Pertea, A. E. Jaffe, B. Langmead, S. L. Salzberg, J. T. Leek, *Nat. Biotechnol.* **2015**, *33*, 243-246.

[13] M. Pertea, D. Kim, G. M. Pertea, J. T. Leek, S. L. Salzberg, *Nat. Protoc.* **2016**, *11*, 1650-1667.

[14] M. S. Lukesch, T. Pavkov-Keller, K. Gruber, K. Zangger, B. Wiltschi, *Sci. Rep.* **2019**, *9*, 1-9.

[15] E. Zandvoort, E. M. Geertsema, B. J. Baas, W. J. Quax, G. J. Poelarends, *Angew. Chem. Int. Ed.* **2012**, *124*, 1266-1269.

[16] T. Narancic, J. Radivojevic, P. Jovanovic, D. Francuski, M. Bigovic, V. Maslak, V. Savic, B. Vasiljevic, K. E. O'Connor, J. Nikodinovic-Runic, *Bioresour. Technol.* **2013**, *142*, 462-468.

[17] T. Liu, M. I. Abboud, R. Chowdhury, A. Tumber, A. P. Hardy, K. Lippl, C. T. Lohans, E. Pires, J. Wickens, M. A. McDonough, *J. Biol. Chem.* **2020**, *295*, 16545-16561.
